# CCL20 Released by Drug–Tolerant Persisters Impairs Immunotherapy in *EGFR–*Mutant Lung Adenocarcinoma

**DOI:** 10.1002/mco2.70888

**Published:** 2026-08-03

**Authors:** Hoi‐Hin Kwok, Jiashuang Yang, Nerissa Chui‐Mei Lee, Junyang Deng, David Chi‐Leung Lam

**Affiliations:** ^1^ Department of Medicine Li Ka Shing Faculty of Medicine University of Hong Kong Hong Kong SAR China

**Keywords:** CCL20, drug‐tolerant persisters, EGFR, immunotherapy, lung adenocarcinoma

## Abstract

*EGFR*‐mutant lung adenocarcinoma (LUAD) is typically associated with an immunosuppressive tumor immune microenvironment (TIME) and poor responses to PD‐1 blockade. However, the contribution of drug‐tolerant persister cells (DTPs) to immunotherapy resistance remains unclear. We hypothesized that DTPs‐derived chemokine (C‐C motif) ligand 20 (CCL20) promotes immune evasion and impairs PD‐1‐based immunotherapy in *EGFR*‐mutant. To test this, humanized NSG mice engrafted with *EGFR*‐mutant H1975 cells and human peripheral blood mononuclear cells (*n* = 6 per group) were administered with anti‐PD‐1 (200 µg) and/or anti‐CCL20 (20 µg) every other day for a total of four doses. The combination reduced mean tumor volume by 65% compared with control treatment (*p* < 0.01). Single‐cell RNA sequencing showed that cotreatment selectively suppressed *CCL20* expression in DTP clusters and activated interferon‐α and ‐γ signaling (ISG15, CMPK2). Multiplex immunofluorescence revealed combination treatment increased infiltration of M1‐like macrophages, plasmacytoid dendritic cells, and memory B cells, alongside spatial segregation of CD4^+^ regulatory T cells (*p* < 0.05). These findings identify DTPs‐derived CCL20 as a mediator of immunosuppressive TIME and support combined CCL20/PD‐1 blockade as a potential therapeutic strategy for *EGFR*‐mutant lung adenocarcinoma.

## Graphical abstract

Schematic representation of tumor immune microenvironment (TIME) modulation by combined anti‐PD‐1 and anti‐CCL20 therapies. Left: In the “cold” tumor TIME, high CCL20 secretion by drug‐tolerant persister (DTP) cancer cell subpopulations promotes regulatory T cell (Treg) enrichment and clusters in close proximity to tumor cells, resulting in immune exclusion through low cytotoxic T cell (CD8^+^) infiltration, downregulated interferon signaling, and upregulated NF‐κB‐driven survival pathways. Right: Dual blockade of PD‐1 and CCL20 leads to suppression of CCL20 expression, contraction of DTP clusters, increased interferon‐α/γ signaling in tumor and immune cells, increased infiltration of cytotoxic lymphocytes and antigen‐presenting cells, and spatial segregation of Tregs, culminating in a remodeled, immunogenic (“hot”) TIME. Illustrated cell types in the tumor microenvironment are indicated in the legend: cancer cells, DTPs, dying DTPs, Tregs, CD4^+^ T cells, CD8^+^ T cells, M1‐macrophages, plasmacytoid dendritic cells, and memory B cells.

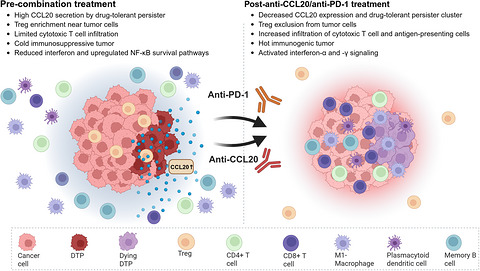

## 1 Introduction

Lung cancer remains a leading global cause of cancer‐related mortality. Lung adenocarcinoma (LUAD), the predominant histological subtype of non‐small‐cell lung cancer (NSCLC), accounts for a significant proportion of cases. Treatment options for advanced‐stage LUAD include systemic therapies, such as chemotherapy, targeted therapy, or immunotherapy, which are often combined with radiotherapy for enhanced efficacy. For patients with advanced‐stage LUAD harboring actionable mutations such as *EGFR*, *ALK*, and *KRAS*, upfront targeted therapy is the standard of care. However, even with highly effective first‐line EGFR‐targeted therapy such as osimertinib, disease progression is an inevitable clinical challenge. Following this event, subsequent therapeutic strategies are crucial and often contribute to a significant proportion of patients surviving beyond the first year [1]. Among these, platinum‐based chemotherapy is usually a second‐line regimen following progression on target therapies. But, in metastatic *EGFR‐*mutant LUAD, immune checkpoint inhibitors (ICIs) are not preferred as first‐line treatment because response rates are generally lower than in EGFR wild‐type NSCLC [2, 3].

To understand the unfavorable response to ICI in *EGFR‐*mutant LUAD, and to explore their potential in other settings, it is crucial to examine the tumor immune microenvironment (TIME). Infiltration of immune cells into the TIME is critical for ICIs efficacy [4, 5]. The TIME is a complex and dynamic network of diverse cell types, cytokines, chemokines, and other signaling molecules that surround and infiltrate the tumor. Recent single‐cell transcriptomic and proteomic studies have shown that LUAD is frequently associated with increased frequencies of regulatory T cells (Tregs) and tumor‐associated macrophages (TAMs) [6], which contribute to an immunosuppressive TIME and reduced ICI responsiveness [7]. Various mechanisms and therapeutic strategies are under investigation to modulate TIME in order to improve ICIs responsiveness in LUAD patients [8].

Cancer cell plasticity is a central driver of resistance in both targeted therapy and immunotherapy [9], particularly through the emergence of drug‐tolerant persisters (DTPs) [10]. DTPs are subclones of cancer cells with diverse genomic variations arising from clonal selection. These cells survived anti‐cancer treatment through nongenetic adaptive mechanisms and gradually regain proliferative capacity after drug withdrawal, leading to drug tolerance and disease relapse [11]. It has been suggested that distinct drug treatments may give rise to different DTPs [12]. Nevertheless, although our understanding of DTP biology has advanced, translating this knowledge into effective therapies has lagged, hindering the selection of optimal regimens.

Instead of focusing solely on driver mutations, an emerging concept is to therapeutically target the non‑genetic determinants of tumor persistence and evolution. Novel treatment strategies, including metabolic rewiring disruptors (e.g., metformin), epigenetic and transcriptional inhibitors (e.g., mTOR or PRDM9 inhibitors), and microenvironment inhibitors (anti‐CD24 and anti‐VEGF), are being explored as adjuncts to, or in rational combinations with, conventional TKIs and chemotherapy [13, 14, 15, 16]. By interfering with central metabolic pathways, chromatin remodeling programs, and stromal or immune‐derived survival cues, these approaches aim to weaken the driving forces of tumor cell plasticity and limit the emergence of potential DTPs, which collectively induce disease recurrence [17]. A more complete understanding of how these pathways interact in space and time during treatment will be essential for designing combination regimens that durably suppress or eradicate DTP populations.

However, the role of DTPs in the modulation of TIME remains largely unknown [18]. Accumulating evidence suggests that, beyond their intrinsic survival advantages, DTPs may adopt distinct secretory phenotypes and stress‐response programs that are capable of reshaping local immune landscapes. However, the interaction between DTPs and various immune cells through paracrine or juxtracrine signaling may facilitate cancer growth [19]. For example, DTP‐derived cytokines, chemokines, and extracellular vesicles can recruit immunosuppressive cell subsets, alter antigen‐presenting cell function, and blunt effector T‐cell activity, thereby creating a niche permissive for tumor regrowth. Our previous single‐cell transcriptomic analysis showed that DTPs in LUAD release chemokine (C‐C motif) ligand 20 (CCL20). CCL20 is a key ligand for the chemokine receptor CCR6 and has been implicated in the recruitment of Treg, Th17 cells, and other CCR6‐expressing immune populations, which may modulate the TIME and contribute to chemoresistance [20]. In this study, we hypothesized that inhibiting CCL20 released by DTPs could remodel the TIME and enhance ICI responsiveness on *EGFR‐*mutant LUAD. By dissecting the cellular sources and downstream targets of CCL20 within the LUAD TIME, we aim to provide a rationale for combinatorial strategies that pair EGFR‐targeted therapy and ICIs with agents that specifically disrupt DTP–immune cell crosstalk.

## 2 Results

### 2.1 Presence of *CCL20*‐Expressing DTPs Is Associated With ICI Responsiveness in *EGFR‐*Mutant Lung Adenocarcinoma

Compared with normal lung tissue in the Genotype‐Tissue Expression (GTEx) database, *EGFR‐*mutant LUAD samples from the TCGA‐LUAD cohort showed significantly higher *CCL20* expression (Figure S1A). Elevated *CCL20* level was also associated with poorer overall survival in *EGFR‐*mutant LUAD patients (Figure S1B).

**FIGURE 1:**
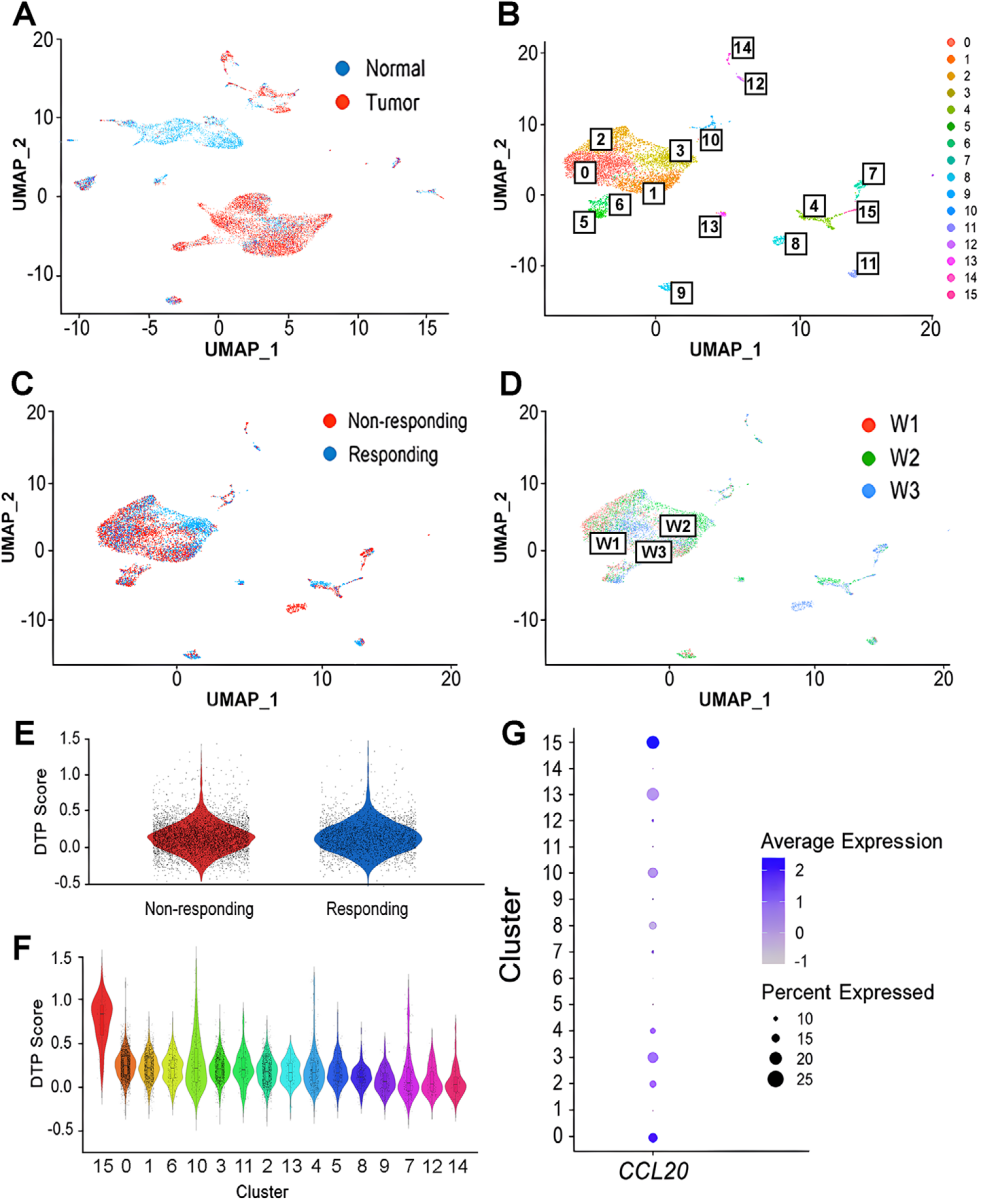
Identification of *CCL20‐*expressing drug‐tolerant persister (DTP) subpopulations in nonresponding nodules after neoadjuvant pembrolizumab from the dataset GSE146100. (A) UMAP projection of total cells from GSE146100, colored by inferred cell type (normal: blue; tumor: red) using copyKAT‐based copy number variation analysis. (B) UMAP projection of tumor cells only, colored by cluster identity. (C) UMAP projection of tumor cells, colored by response status (nonresponding nodules: red; responding nodule: blue). (D) UMAP projection of tumor cells, colored by nodule origin (nonresponding nodules W1 (red) and W3 (green); responding nodule W2 (blue)). (E) Violin plots comparing DTP scores between nonresponding and responding nodules. (F) Violin plots of DTP scores across tumor cluster. (G) Dot plot of *CCL20* expression across tumor clusters, with dot size indicating percent of cells expressing *CCL20* and color representing average expression level (scaled).

To investigate the presence of drug‐tolerant persister (DTP) subpopulations in lung adenocarcinoma and their potential association with immunotherapy response, we re‐analyzed the single‐cell RNA sequencing dataset GSE146100, derived from a patient with multiple primary lung nodules treated with three cycles of neoadjuvant pembrolizumab. Tumor cells were separated from normal cells using copyKAT‐based copy number inference (Figure 1A), and tumor subpopulations were identified through UMAP‐based clustering (Figure 1B). The dataset comprised samples from one responding nodule (W2) and two nonresponding nodules (W1 and W3; Figure 1C,D). Although aggregate DTP scores did not differ significantly between responding and nonresponding nodules (Figure 1E), cluster 15 displayed the highest DTP score (Figure 1F) and the strongest *CCL20* expression (Figure 1G).

**FIGURE 2:**
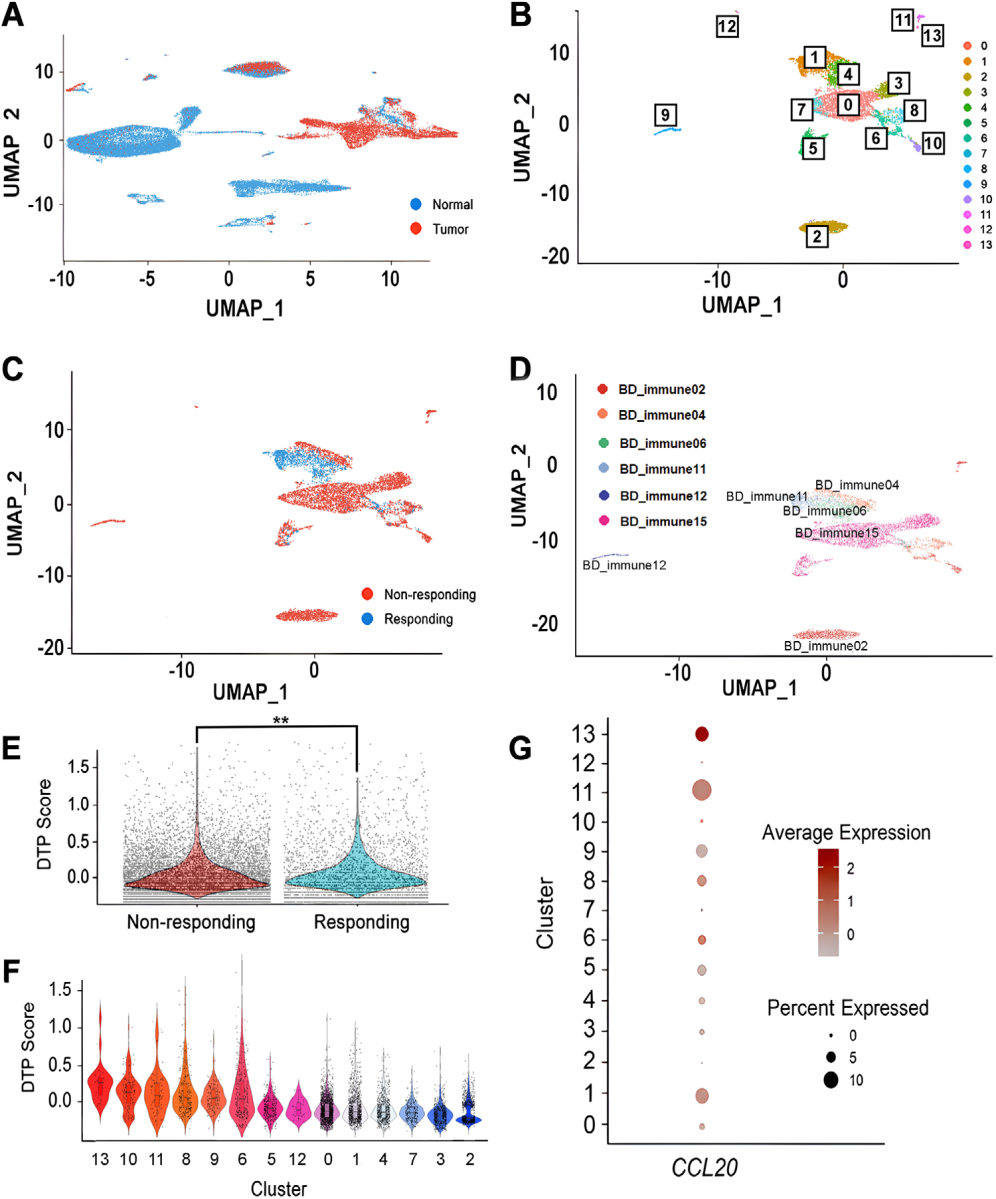
Validation of *CCL20*‐expressing drug‐tolerant persister (DTP) subpopulations enrichment in post‐neoadjuvant immunotherapy lung adenocarcinoma from the dataset GSE207422. (A) UMAP projection of total cells from GSE207422, colored by inferred cell type (normal: blue; tumor: red) using copyKAT‐based copy number variation analysis. (B) UMAP projection of tumor cells only, colored by cluster identity. (C) UMAP projection of tumor cells, colored by response status (nonresponding samples: red; responding samples: blue). (D) UMAP projection of tumor cells, colored by responder samples (BD_immune06: green; BD_immune11), and nonresponder samples (BD_immune02: red; BD_immune04: yellow;: blue; BD_immune12: cyan; BD_immune15: magenta). (E) Violin plots comparing DTP scores between nonresponding and responding samples. DTP scores were modestly higher in nonresponding samples (Wilcoxon rank‐sum test, *p < 0.05). (F) Violin plots of DTP scores across tumor clusters. (G) Dot plot of *CCL20* expression across tumor clusters, with dot size indicating percent of cells expressing CCL20 and color representing average expression level (scaled).

To further validate the association between CCL20‐expressing DTP subpopulations and immunotherapy responsiveness in EGFR‐mutant LUAD, we re‐analyzed the single‐cell RNA sequencing dataset GSE207422, comprising post‐treatment tumor samples from six lung adenocarcinoma patients who received neoadjuvant anti‐PD‐1 or anti‐PD‐L1 therapy. Tumor cells were separated from nonmalignant cells using copyKAT‐based copy number variation inference (Figure 2A), and tumor subpopulations were identified through UMAP‐based clustering (Figure 2B). The dataset included samples from two responders (BD_immune06 and BD_immune11) and four nonresponders (BD_immune02, BD_immune04, BD_immune12, and BD_immune15; Figure 2C,D). Aggregate DTP scores were modestly elevated in nonresponding samples compared with responding samples (Figure 2E). Consistent with findings from GSE146100, the tumor subpopulation with the highest DTP score (cluster 13) also exhibited the highest *CCL20* expression among all tumor clusters (Figure 2F,G). Gene set enrichment analysis (GSEA) confirmed that both putative DTP clusters (cluster 15 from GSE146100 and cluster 13 from GSE207422) displayed metabolically specialized and stress‐adapted phenotypes (Figure S2).

### 2.2 Combination Of Anti‐PD‐1 and Anti‐CCL20 Enhances Therapeutic Effects in *EGFR‐Mutant* Lung Adenocarcinoma

**FIGURE 3:**
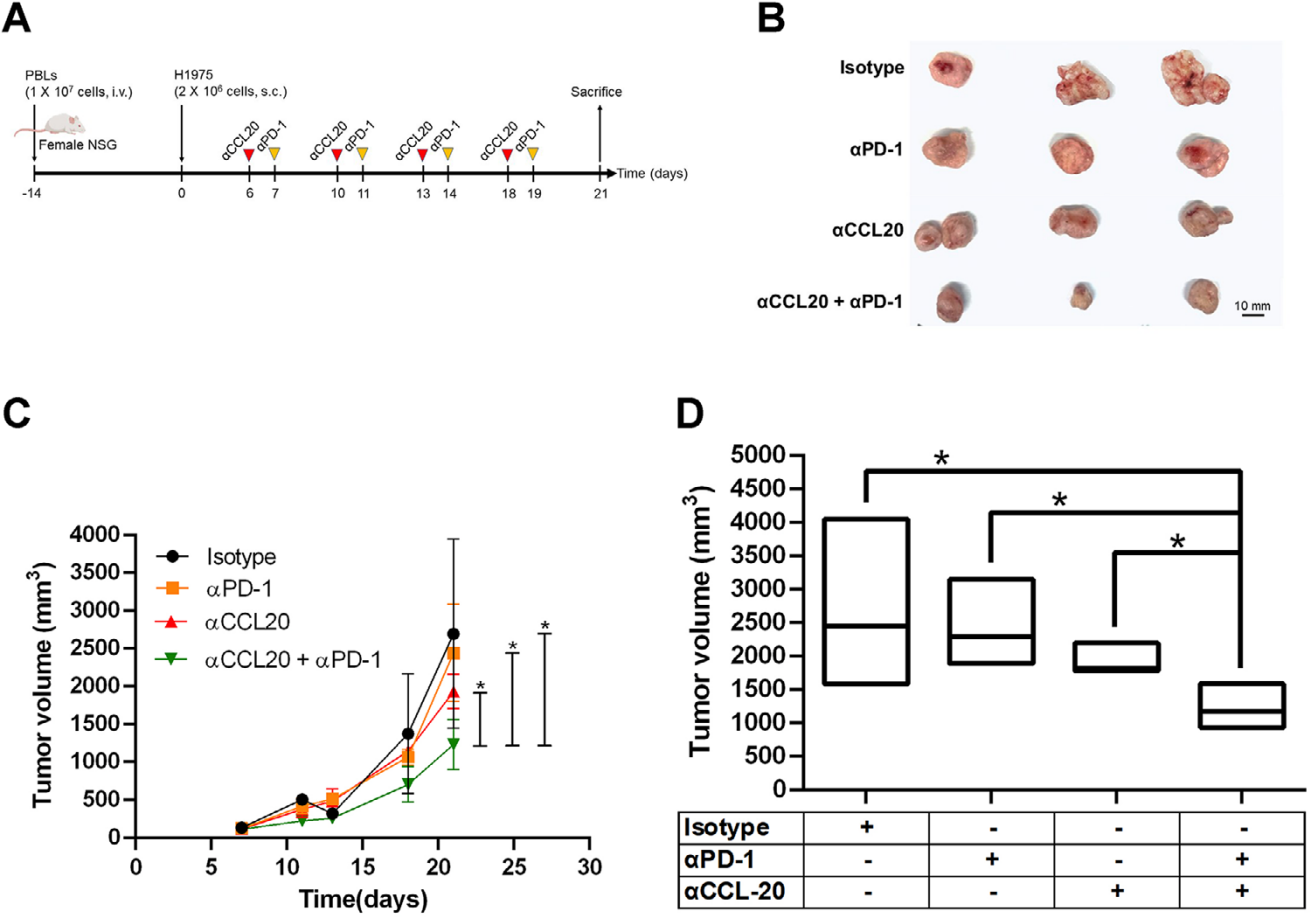
Combination of anti‐PD‐1 and anti‐CCL20 therapies enhances therapeutic efficacy in *EGFR*‐mutant lung adenocarcinoma mouse model. (A) Schematic timeline of the in vivo experiment using humanized NSG mice. PBMCs (1 × 10^7^ cells, i.v.) were injected on day −14, followed by subcutaneous injection of NCI‐H1975 cells (2 × 10^6^ cells) on day 0. Treatment with anti‐PD‐1 (200 µg/mouse, i.p.) or anti‐CCL20 (20 µg/mouse, i.p.) began when tumors reached ∼200 mm^3^ (day 7), administered every other day for four cycles, with mice sacrificed on day 21. (B) Representative images of resected tumors from each treatment group at day 21 (scale bar = 10 mm). (C) Tumor growth curves showing mean tumor volume (mm^3^) over time for isotype control (*n* = 6), anti‐PD‐1 (*n* = 6), anti‐CCL20 (*n* = 6), and combination (anti‐CCL20 + anti‐PD‐1, *n* = 6) treatment groups. Data are presented as mean ± SEM; *p < 0.05 (one‐way ANOVA followed by Tukey's post hoc test). (D) Box plot comparing final tumor volumes (mm^3^) across treatment groups, with statistical significance indicated by asterisks (*p < 0.05, one‐way ANOVA followed by Tukey's post hoc test). The table below summarizes the treatment conditions.

To determine the role CCL20 in *EGFR‐mutant* LUAD, humanized mice were treated with four cycles of alternate day treatment of anti‐CCL20 and anti‐PD‐1 neutralizing antibody, then the mice were sacrificed and tumor was harvested (Figure 3A). Neither anti‐PD‐1 nor anti‐CCL20 monotherapy significantly reduced tumor growth compared with the isotype control. In contrast, the combination of anti‐CCL20 and anti‐PD‐1 markedly suppressed tumor progression, reducing mean tumor volume by 65% at day 21 (*p* < 0.05; Figure 3B,D).

**FIGURE 4:**
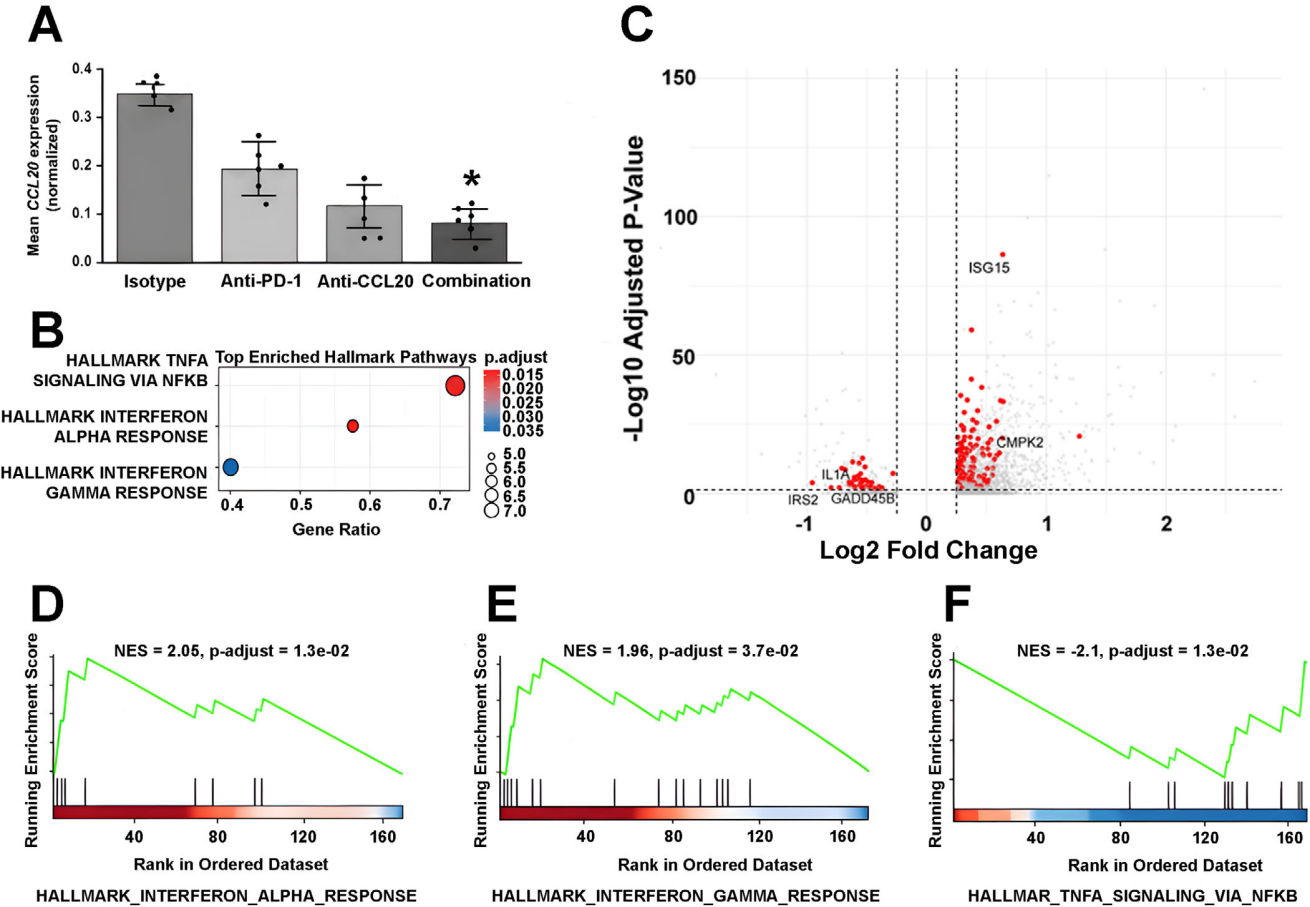
Combination of anti‐PD‐1 and anti‐CCL20 therapies modulates *CCL20* expression and transcriptional profiles in *EGFR*‐mutant lung adenocarcinoma. (A) Bar chart showing *CCL20* expression levels across treatment groups (isotype control, anti‐PD‐1, anti‐CCL20, and combination) in xenograft tumors, showing significant reduction in *CCL20* expression with combination therapy (*p < 0.05, one‐way ANOVA followed by Tukey's post hoc test) (*n* = 6). (B) Dot plot of top enriched gene set enrichment analysis (GSEA). (C) Volcano plot of differentially expressed genes in combination versus isotype control (red: significantly upregulated; *p* < 0.05, adjusted). Gene set enrichment plots for the (D) interferon‐α response, (E) interferon‐ɣ response, and (F) TNF‐α signaling.

Single‐cell RNA sequencing of harvested xenografts (22,982 cells after quality filtering) showed that combination treatment produced the strongest reduction in CCL20 expression across tumor cells (Figure 4A). Differential gene expression (DGE) analysis revealed unique upregulation of interferon‐α and ‐γ response genes (e.g., ISG15 and CMPK2) and downregulation of NF‐κB pathway components (IRS2, IL1A, and GADD45B) in the combination arm (Figure 4B–F).

### 2.3 Combination Of Anti‐PD‐1 and Anti‐CCL20 Perturbed DTPs Transcriptional Profile and Disrupt Tumor Clonal Interactions

**FIGURE 5:**
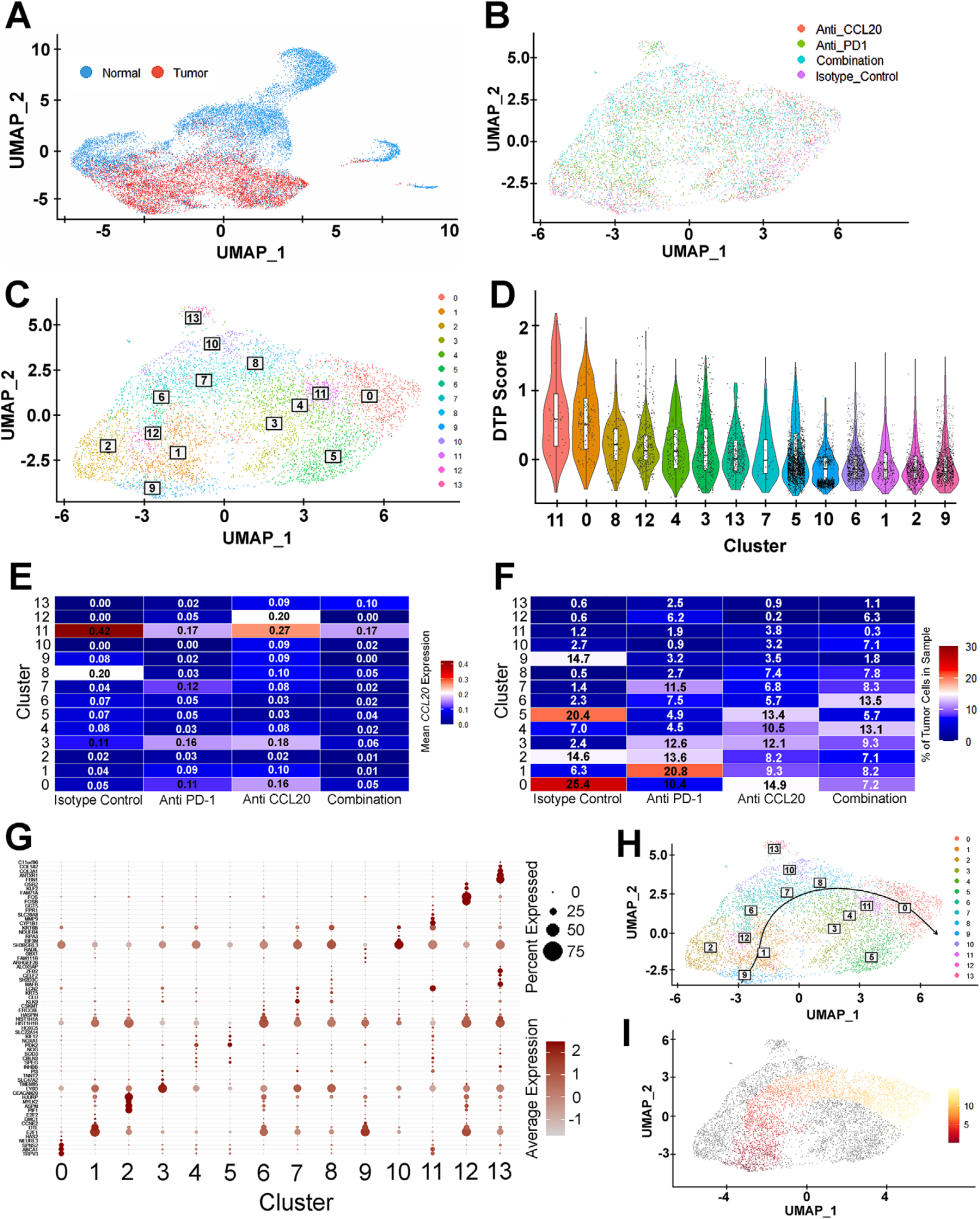
Single‐cell transcriptomics of xenograft tumors demonstrates combination of anti‐PD‐1 and anti‐CCL20 therapies perturbs *CCL20* expression in DTPs. (A) UMAP projection of total cells colored by inferred cell type (normal: blue; tumor: red). (B) UMAP projection of tumor cells colored by treatment group. (C) UMAP projection of tumor cells colored by cluster identity (14 clusters). (D) Violin plots of DTP scores across tumor clusters. (E) Heatmap of mean CCL20 expression per cluster across treatment groups. (F) Heatmap of the percentage of tumor cells per cluster across treatment groups. (G) Dot plot of top DEGs across clusters (dot size = percentage of expressing cells; color = scaled average expression). Developmental trajectory of tumor subpopulations is shown by clusters (H) or pseudotime (I).

Single‐cell RNA sequencing of xenograft tumors revealed treatment‐specific remodeling of the tumor cell compartment. After distinguished tumor cells from normal cells (Figure 5A), tumor cells from the four experimental groups were separated by UMAP clustering (Figure 5B). Subclustering identified 14 tumor subpopulations (Figure 5C), of which cluster 11 and 0 exhibited the highest DTP score (Figure 5D). Further comparison showed that combination treatment suppressed CCL20 expressions in both cluster 11 and 0 (Figure 5E). The percentage of cluster 11 and 0 also greatly inhibited under combination treatment compared to isotype control, anti‐PD‐1 alone, or anti‐CCL20 alone (Figure 5F), echoing the results with our prior analyses of human datasets. DEG analysis revealed that tumor cluster 11 overexpressed pro‐tumorigenic factor LCN2 and MMP9. Cluster 0 expressed high level of TRPV3, ABCA1, and SPNS2, which are related to metastasis (Figure 5G). Trajectory and pseudotime analyses indicated that cluster 11 represents an early, progenitor‐like DTP state that can give rise to the more proliferative cluster 0 (Figure 5H,I). Further GSEA pathway analysis showed activation of inflammatory (pathways including inflammatory response, IL6‐JAK‐STAT3, and TNFA signaling‐NFKB) and stem‐like features (pathways including KRAS‐up, epithelial mesenchymal transition, IL6‐JAK‐STAT3) in cluster 11, suggesting it represents DTPs, capable of self‐renewal and giving rise to progeny like cluster 0. As the tumor progresses, cells transition to cluster 0's proliferative state (IL2/STAT5 signaling), adapting to hypoxia and sustained growth (KRAS‐up and TNFA signaling via NFKB) (Figure S3).

### 2.4 CCL20 And PD‐1 Coinhibition Transform Tumor Immune Landscape

**FIGURE 6:**
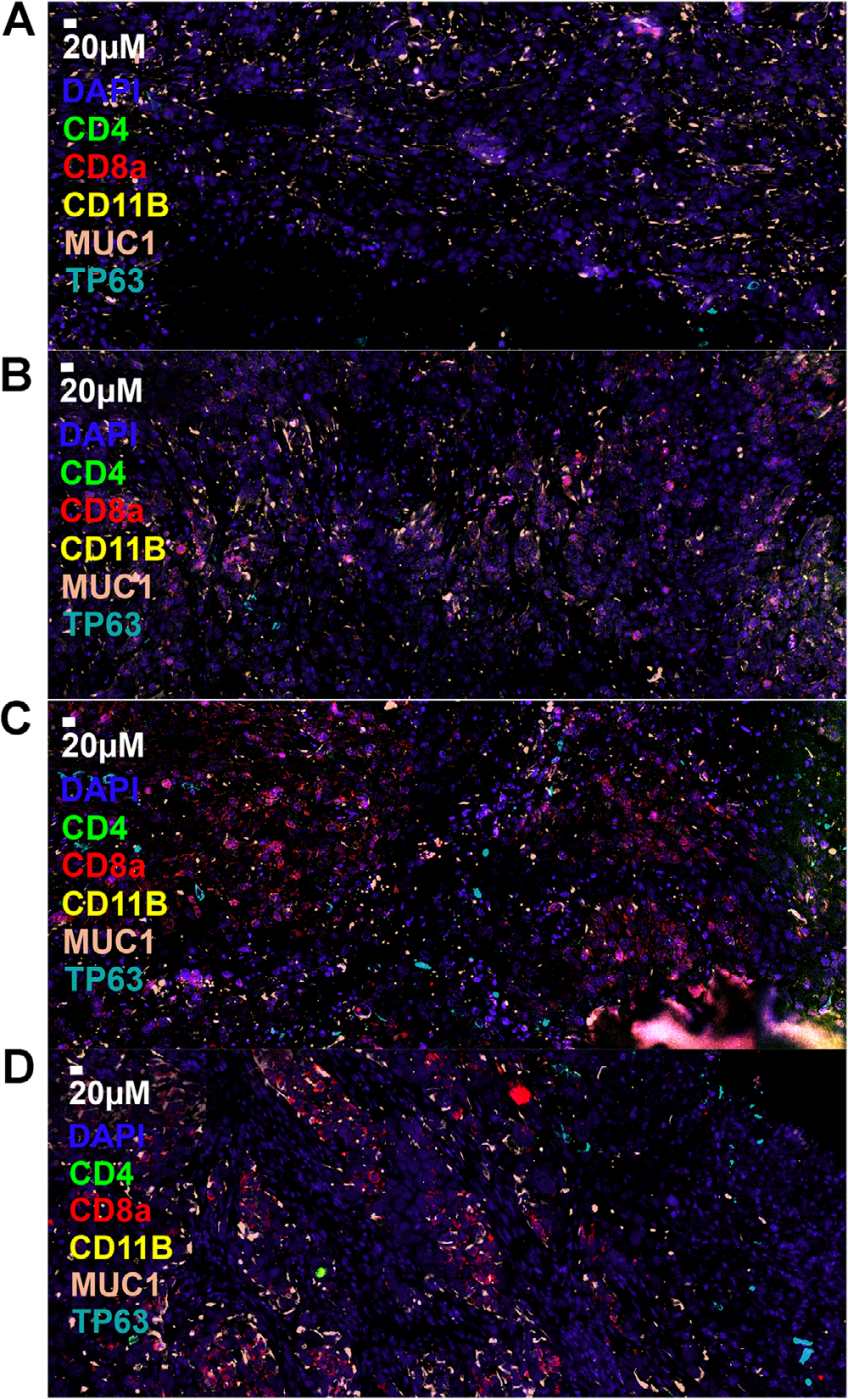
Representative images of multiplex immunofluorescence microscopy on the xenograft. Representative images are from (A) isotype control, (B) anti‐PD‐1, (C) anti‐CCL20, and (D) combination groups. Selected signals of immune markers (CD4 (Green), CD8a (Red), and CD11b (Yellow)) and tumor markers (MUC1 (Pink) and TP63 (Cyan)) are shown. Scale bars, 20 µm.

**FIGURE 7:**
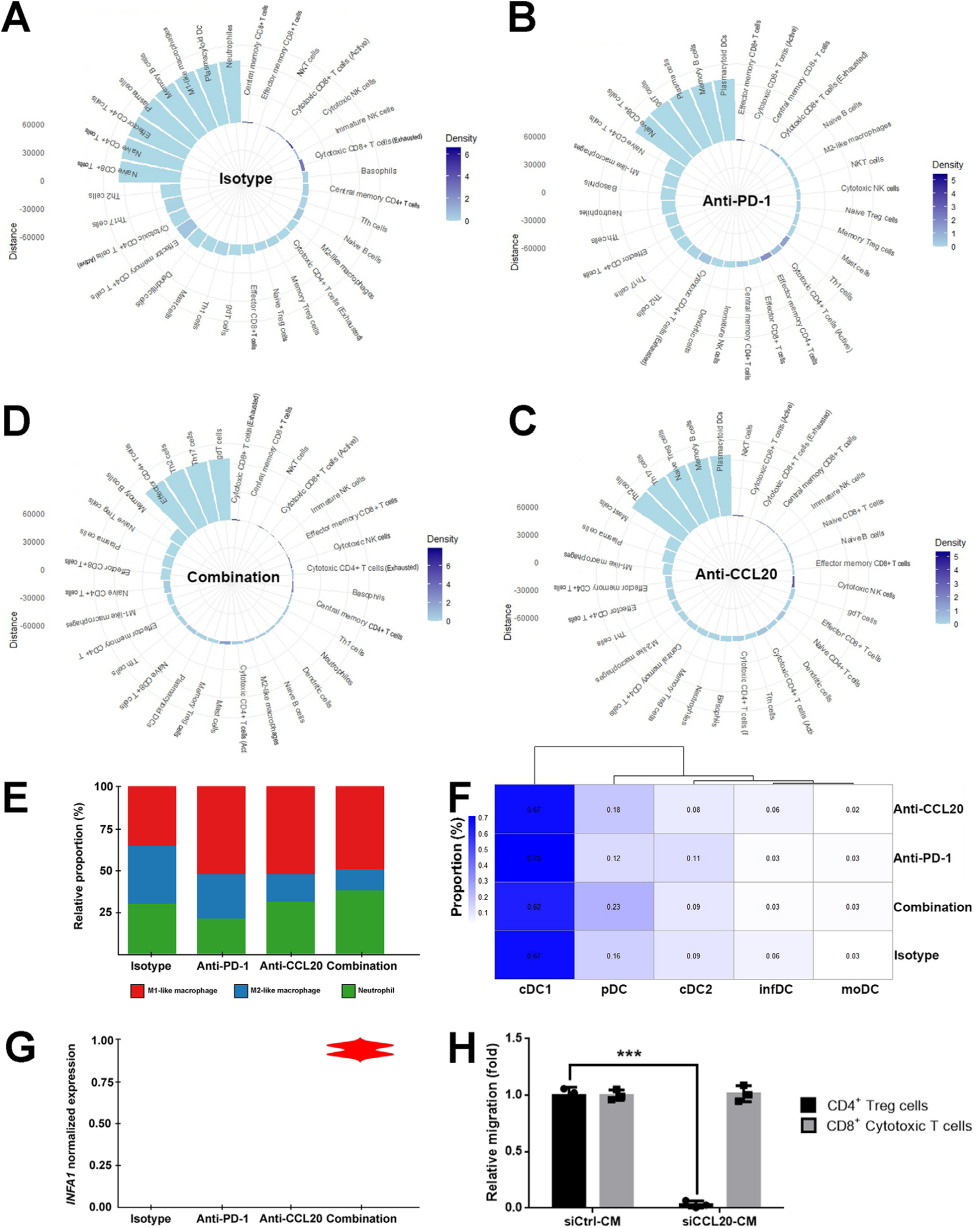
CCL20 and PD‐1 co‐inhibition reduces infiltration of CD4^+^ Treg cells in *EGFR‐*mutant lung adenocarcinoma. Spatial‐density distribution plots from multiplex immunofluorescence microscopy showing the density and distance of various immune cell types in xenograft tumors from (A) isotype control, (B) anti‐PD‐1, (C) anti‐CCL20, and (D) combination groups. (E) Stacked bar plot showing the relative density (proportions %) of M1‐like (red), M2‐like (blue) macrophages, and neutrophils (green) across treatment groups. (F) Heatmap of dendritic cell phenotype density (proportion %) across treatment groups. (G) Bar graph of *IFNA1* expression in M1‐like macrophages across treatment groups. (H) Chemotactic migration of CD4^+^ Treg and CD8^+^ cytotoxic T cells in response to conditioned media from control or CCL20‐silenced H1975 cells, and migrated cells were quantified by flow cytometry after incubation for 24 h. Relative migration was normalized to the siCtrl‐conditioned medium (siCtrl‐CM) group and is presented as fold change. Data are shown as mean ± SD of three independent experiments (*p < 0.05, one‐way ANOVA followed by Tukey's post hoc test) (*n* = 3).

To elucidate the detailed immune contexture within the tumor, we conducted deep multiplex immunofluorescence microscopy on the resected xenograft (Figure 6) and performed quantitative analyses (Figure 7A–D). Pairwise spatial analysis indicated that the combination treatment significantly induced spatial aggregation between cytotoxic CD4^+^ T cells and naïve Treg (Table 1) when compared with isotype treatment group.

**TABLE 1:** Pairwise spatial analysis revealed key changes in immune cells interactions.

Sample	Cell type A	Cell type B	ASI	Delta_a	*p* value
Combination	Cytotoxic CD4^+^ T cells	Naive Treg cells	1.039	−1,278,445	0.002
Anti‐PD‐1	Effector CD4^+^ T cells	Immature NK cells	3.203	201,836,850	0.018
Combination	Naive Treg cells	Neutrophils	0.687	−13,063,234	0.058
Combination	Immature NK cells	Naive Treg cells	0.417	−38,536,150	0.060
Anti‐PD‐1	Dendritic cells	NKT cells	3.149	52,780,019	0.097

Significant interactions derived among the four xenograft samples were highlighted. ASI (Association Index) quantifies the level of spatial association between two cell types. Delta_a value represents the average normalized deviation of the observed spatial pattern from complete spatial randomness (CSR) across all analyzed distances.

When compared with the other treatment groups, the combination treatment caused Th17 and Th1 cells to retreat from the tumor (Table S1). It also led to increased densities of M1‐like macrophages and neutrophils, but a decreased density of M2‐like macrophages (Figure 7E). In addition, the density of plasmacytoid DCs (Figure 7F) was also increased compared to the other treatment groups. Higher IFNA1 expression in M1‐like macrophages suggest a more inflammatory tumor microenvironment (Figure 7G). In vitro migration assays confirmed that, incubation of conditioned medium from H1975 cancer cells with CCL20 knockdown, fully abolished CD4^+^ Treg but not CD8^+^ cytotoxic T cells recruitment (Figure 7H), further supporting that CCL20 primarily drives Treg‐mediated immunosuppression. These findings suggest that combined anti‐CCL20 and anti‐PD‐1 treatment exerts a synergistic effect in modulating the tumor immune landscape. This shift renders the tumor more immunogenic, facilitating enhanced interactions between effector T cells and tumor cells, and surpasses the effects of either anti‐PD‐1 or anti‐CCL20 treatment alone.

## 3 Discussion

*EGFR‐*mutant lung adenocarcinoma (LUAD), the most common subtype of non‐small‐cell lung cancer (NSCLC), particularly among Asian populations, poses significant therapeutic challenges due to the inevitable development of resistance to EGFR tyrosine kinase inhibitors (TKIs) such as osimertinib. While immune checkpoint inhibitors (ICIs) have revolutionized treatment paradigms for lung cancer, their efficacy in *EGFR‐*mutant LUAD is limited, as evidenced by different clinical trials showing lower response rates to ICI in this subgroup compared to *EGFR* wild‐type NSCLC. This diminished responsiveness is attributed to an immunosuppressive tumor immune microenvironment (TIME), characterized by low immune cell infiltration and a “cold” immune phenotype. In this study, we demonstrated that DTPs in *EGFR‐*mutant LUAD released CCL20, a chemokine that fostered immune privilege and contributed to ICI resistance. By inhibiting CCL20 in combination with anti‐PD‐1 therapy, we observed a synergistic anti‐tumor effect, suggesting a potential novel therapeutic strategy to enhance ICI efficacy in this patient population.

CCL20, a chemokine ligand for CCR6, is known to recruit Tregs and Th17 cells, both of which contribute to an immunosuppressive TIME in various cancers, including colorectal cancer (CRC), where elevated *CCL20* levels are similarly linked to poor survival, chemoresistance [21], and tumor stemness [22]. In *EGFR‐mutant* LUAD, previous studies have shown that CCL20 expression was regulated by transcription factor *RUNX3* and induced by the convergence of TGF‐β and IL‐1β signaling pathways [23], which may be activated in tumor cells as part of their adaptive response to therapy [24]. Our data from spatial analysis provide novel insight into how DTPs may exploit CCL20 to create an immune‐privileged niche, thereby shielding themselves from immune surveillance and therapeutic pressure by innate immune cells such as neutrophils and macrophages. This mechanism aligns with the observed heterogeneity of the TIME in LUAD, possibly determined by DTP occurrence.

To investigate the therapeutic potential of targeting CCL20, we employed a humanized NSG mouse model engrafted with H1975 cells, an *EGFR‐*mutant LUAD cell line harboring *L858R* and *T790M* mutations. While anti‐PD‐1 monotherapy showed no significant effect on tumor growth compared to the isotype control, and anti‐CCL20 alone yielded only a modest, nonsignificant reduction, this may reflect compensatory CCL20 production by other tumor subpopulations (Figure 5E). While the combination of anti‐CCL20 and anti‐PD‐1 markedly suppressed tumor growth, this synergy suggests that CCL20 inhibition disrupts the immunosuppressive signals driven by DTPs, reshaping the TIME and thereby sensitizing the tumor to PD‐1 blockade. Single‐cell transcriptomic analysis of the tumor subpopulation indicates that while PD‐1 or CCL20 blockade alone can affect the tumor subpopulation composition (Figure 5F), only the combination treatment can reduce the proliferative DTP‐derived progeny (cluster 0). This reduction may be key to remodulating the dynamics of immune cells and facilitating more active immune surveillance.

Using deep multiplex immunofluorescence microscopy, we were able to compare the spatial TIME of xenograft comprehensively under different treatment. By remolding the TIME dynamic, combination treatment induced exit of Th2, Th17, and γδT cells, and recruitment of memory B cells and plasmacytoid DCs. Indeed, with the development of single‐cell transcriptomic, mounting evidence suggested the multifaceted functions of each immune cell type. Their contribution in TIME was largely dependent on the local cell–cell interactions and metabolic regulation [25].

The limited efficacy of ICIs in *EGFR‐*mutant LUAD has spurred investigation into combination therapies that modulate TIME or target resistance mechanisms such as DTPs. Our findings positioned CCL20 inhibition as a promising adjunct to PD‐1 blockade, aligning with broader efforts to combine ICIs with chemotherapy, targeted therapies, or other immunomodulators. By addressing the immunosuppressive role of DTP‐derived CCL20, this strategy “heats up” or activates the typically cold TIME of *EGFR‐*mutant LUAD, enhancing immune cell infiltration and ICI responsiveness. Our previous study also demonstrated that DTPs in *ALK*‐rearranged LUAD release CCL20^20^, suggesting that CCL20 secretion from DTPs may contribute to the poor response to ICIs observed clinically in oncogene‐driven LUAD. Given that DTPs are the primary source of CCL20, strategies to directly eliminate these cells could further enhance outcomes, though their plasticity and adaptability present significant challenges.

Our in vitro and in vivo data converge on a unified mechanism in which CCL20 functions predominantly as a Treg‐recruiting chemokine in *EGFR*‐mutant LUAD. Selective knockdown of CCL20 in H1975 cells abolished Treg recruitment without impairing cytotoxic CD8^+^ T‐cell migration, directly demonstrating that the immunosuppressive effect of CCL20 is Treg‐dependent rather than a generalized chemotactic signal. This finding aligns with the observed spatial reorganization in the xenograft model, where combination therapy first reduced naïve Treg infiltration and proximity to tumor cells, followed by secondary increases in neutrophil and other effector cell recruitment. The latter likely reflects de‐repression of interferon‐driven inflammatory programs once Treg‐mediated suppression is relieved. Collectively, these results position CCL20 as a key upstream regulator of the immunosuppressive tumor immune microenvironment in *EGFR*‐mutant LUAD. By targeting this axis, dual CCL20/PD‐1 blockade not only disrupts the maintenance of drug‐tolerant persister states but also triggers a cascade of immune remodeling that anti‐PD‐1 therapy alone fails to achieve. This hierarchical model explains the superior efficacy of the combination therapy by demonstrating how it inhibits DTP‐derived CCL20, thereby preventing Treg recruitment and alleviating broad immunosuppression. Consequently, these findings establish CCL20 as both a promising therapeutic target and a valuable biomarker for identifying appropriate patient populations in future clinical trials.

Different combinations of ICIs and other therapies are currently under investigation for *EGFR*‐mutant LUAD [26]. Both top‐down (clinical data‐driven, trial‑driven) and bottom‐up (mechanism‑driven, lab‑driven) approaches are being employed to explore novel therapeutic strategies for *EGFR‐*mutant LUAD. The effects of anti‐PD‐1 or anti‐PD‐L1 are often overlooked because they do more than just block the interactions between PD‐1 on T cells and PD‐L1 on tumor cells. They also inhibit the interactions between PD‐1 and PD‐L1 on other types of immune cells [27]. Spatial analysis from this study revealed the dynamic interactions of various immune cells under anti‐PD‐1 treatment; further study on the spatial dynamics of TIME at single‐cell level in oncogene‐driven LUAD would facilitate the personalized use of ICI combinations in future [28, 29].

Although our study provides strong preclinical evidence that CCL20 released by DTP cells drives immunotherapy resistance in EGFR‐mutant LUAD, several limitations warrant caution. First, relying solely on the H1975 line may limit generalizability to other EGFR mutation subtypes and co‐alterations common in patients, which in turn can affect translational relevance. Second, the humanized NSG‐PBL model, while valuable for human immune responses, lacks a fully reconstituted lymphoid architecture and certain innate compartments, and uses healthy donor PBMCs that may not capture cancer‐associated T‐cell exhaustion; these factors could distort the apparent magnitude of CCL20/PD‐1 co‐blockade effects. Third, our single‐Cell RNA Sequencing (scRNA‐seq) analyses derive from relatively small cohorts and specific contexts, so larger, multi‐center datasets in osimertinib‐ and/or PD‐1–treated EGFR‐mutant patients are needed to confirm prevalence and prognostic value of CCL20+ DTP subpopulations. Fourth, although we show causality of the CCL20‐CCR6 axis in vitro, we did not test CCR6 depletion or blockade in vivo to delineate whether benefits arise mainly from Tregs or other CCR6+ cells. Finally, the study focuses on short‐term tumor growth (21 days) without assessment of metastasis, durable resistance, or immune memory. Collectively, these limitations temper generalizability but do not negate the mechanistic insight that CCL20 mediates DTP‐driven immunosuppression and supports testing CCL20 targeting with PD‐1 blockade in diverse preclinical models and early‐phase trials.

CCL20 and PD‐1 co‐inhibition remodels the TIME from an immunosuppressive to an immunogenic state, overcoming the barriers to ICI efficacy in *EGFR‐*mutant LUAD. These preclinical findings provide a mechanistic basis on role of DTPs in ICI therapy, pursuing CCL20‐targeted combination therapies, and highlight CCL20 as a potential biomarker for ICI responsiveness, paving the way for further clinical trials targeting CCL20 in *EGFR‐*mutant LUAD.

## 4 Methods

### 4.1 Human Cell Line for Tumor Engraftment

Human lung adenocarcinoma cells NCI‐H1975 (H1975) (derived from a female nonsmoker, harboring *EGFR* L858R and T790M double mutations) (Research Resource Identifiers (RRID: CVCL_1511)) were kindly provided by John Minna's laboratory (University of Texas Southwestern University, Dallas, TX, USA). Cells were cultured in RPMI 1640 medium (Thermo Fisher Scientific, Waltham, MA, USA) supplemented with fetal bovine serum (10%, Thermo Fisher Scientific) and penicillin/streptomycin (1%, Thermo Fisher Scientific) at 37°C in 5% CO_2_ incubator. Identity of the H1975 cell line was authenticated by short tandem repeat (STR) DNA profiling assay (Figure S4) (Pangenia Lifesciences Ltd.). Cells were examined for mycoplasma contamination every 6 months.

### 4.2 Preparation of Human Peripheral Blood Mononuclear Cells (PBMCs)

Fresh whole blood from healthy donor was collected by the Hong Kong Red Cross. PBMCs were isolated using Lymphoprep density gradient medium (Stemcell Technology, Vancouver, BC, Canada) according to manufacturer's protocol. The collected PBMCs were aliquoted and stored in liquid nitrogen until experiment. All study participants provided informed consent for sample collection.

### 4.3 Mice

Female NOD.Cg‐Prkdc^scid^Il2rg^1Wjl^/SzJ (NSG) mice (6–8 weeks) were maintained under pathogen‐free conditions according to SPF guideline (room temperature, 40%–60% humidity) at the Centre for Comparative Medicine Research (The University of Hong Kong). The sample size for the in vivo experiments was determined using G*Power software (v3.1.9.7) for one‐way ANOVA followed by Tukey's post hoc test. We aimed to detect a minimum difference in tumor volume of 100 mm^3^ between groups, assuming a standard deviation of 20 mm^3^, with a significance level (*α*) of 0.05 and a statistical power of 0.8. This calculation indicated that a minimum of three animals per group would be sufficient to detect such a difference. However, to account for potential biological variability and possible attrition, we included at least six mice per group in each experiment, in accordance with ARRIVE guidelines and standard practice for preclinical studies.

### 4.4 In Vivo Studies

The Hu‐PBL‐NSG mouse model was established according to previous published method [30]. PBMCs (1 × 10^7^ cells in 100 µL of PBS) were injected in tail vein of mice. After 2 weeks, H1975 cells (2 × 10^6^ cells in 100 µL of PBS and 100 µL of growth factor–reduced basement extract (Trevigen, Gaithersburg, MD, USA)) were injected subcutaneously in the flank regions of mice. When the xenograft reached approximately 200 mm^3^ (around day 7), mice were treated with anti‐PD‐1 (#SIM0010, BioXCell, Lebanon, NH, USA, 200 µg/mouse, i.p.) or anti‐CCL20 (#MAB360, R&D Systems, Minneapolis, MN, 20 µg/mouse, i.p.) [21] diluted in InVivoPure pH7.0 dilution buffer (BioXCell) at every other day for four cycles. RecombiMAb human IgG4 (S228P) isotype control (BioXCell) was used as a negative control. Mice were sacrificed at day 21. Xenografts were harvested, fixed with formalin (10%, Sigma‐Aldrich, St. Louis, MO, USA), and embedded in paraffin for histological analysis, or snap‐frozen for single‐cell transcriptomic analysis.

### 4.5 Multiplex Immunofluorescence Microscopy by Macsima Imaging System

Formalin‐fixed paraffin‐embedded (FFPE) xenograft samples mounted on microscopic slides were deparaffined and rehydrated according to standard procedures. Antigen retrieval was conducted in pH 9 antigen retrieval buffer (#00‐4956‐58, Thermo Fisher Scientific) and transferred to a microwave oven for 8 min at full power, followed by 20 min at the lowest power. The slides were allowed to cool down and stored in MACSima running buffer until staining. The slides were mounted on a MACSwell Four imaging frame (#130‐124‐675, Miltenyi Biotech, Bergisch Gladbach, Germany) and then stained with DAPI staining solution (1:5) 10 min at room temperature in the dark. The slides were then washed three times with MACSima running buffer. Image acquisition of 42 cell surface markers were then captured by the MACSima System (Miltenyi Biotech). The cell surface markers and antibodies used are listed in Table S2. The images were analyzed by MACSima IQ view analysis software (Miltenyi Biotech). The expression of each marker was then gated, and cellular phenotype was determined by a hierarchy of multiple markers (Table S3). Density and distance of each cell type were then measured. Spatial point pattern analysis was performed using the Spatiopath method [31] to quantify pairwise interactions between cell types. For each sample, cell coordinates were used to create a multitype point pattern within a defined observation window. The cross‐type Ripley's K function assessed spatial associations between cell type pairs, applying isotropic edge correction. The Spatial Association Index (ASI) was calculated to indicate clustering (ASI > 1), random distribution (ASI ≈ 1), or repulsion (ASI < 1). The mean deviation of observed from theoretical K, Delta_a, indicated clustering (positive) or repulsion (negative). Statistical significance was tested using Monte Carlo simulation iterations to create 95% confidence envelopes. False discovery rate (FDR) correction was applied to *p*‐values for multiple comparisons within each sample. All the pairwise comparisons are listed in Table S2.

### 4.6 Single‐Cell RNA Sequencing

Xenografts from humanized mice were harvested and snap‐frozen. The samples were dispatched in a blinded manner. To prepare a single cell suspension, tissue was lysed to release the nucleus by adding lysis buffer (Sigma‐Aldrich) and pestling. The pellets including released nucleus and undigested tissue were resuspended and the isolated nuclei were filtered through a 20‐µm cell strainer (Greiner Bio‐OneGmbH, Frickenhausen, Germany). An aliquot of nuclei was stained with DAPI, and concentration and viability were determined on a Cellometer Auto 2000 (Nexcelom Bioscience, Lawrence, MA, USA). The qualified single nuclei were processed for single nuclei RNA‐seq. Nuclei were fixed using the demonstrated protocol (CG000478) from 10X Genomics (Pleasanton, CA, USA). After fixation, the nuclei count and quality assessments were intermittently evaluated using a LUNA dual fluorescence cell counter (Logos Biosystems, South Korea). After probe hybridization, uniquely barcoded samples were counted and then pooled in equal nuclei numbers following manufacturer's protocol (10X Genomics, Chromium fixed RNA profiling multiplexed samples pooling workbook Rev B, CG000565). Pooled nuclei were washed, and 20,000 cells from each sample were targeted to be partitioned into GEMs on a Chip Q, GEM barcoding, GEM recovery and pre‐amplification, and library construction. Sequencing libraries were generated following the user guide (CG000527). Libraries were sequenced on an Illumina NovaSeqX with paired‐end dual‐indexing to a depth of approximately 300 million reads per sample with 2 × 150 read length. Sequencing libraries were demultiplexed with bcl2fastq conversion software (Illumina Inc., San Diego, CA, USA).

### 4.7 Analysis of scRNA‐Seq Data

FASTQ files were processed with Cell Ranger v7.1.0 (10x Genomics) and using GRCh38‐2020‐A as reference. Raw reads were aligned to the human genome (hg38), and cells were detected using Cell Ranger count (v7.1.0). Cells with fewer than 200 detected genes, more than 8,000 detected genes, or a mitochondrial gene contamination rate exceeding 10% were filtered out as low‐quality cells. Moreover, to remove ambient RNA contamination from droplet, we applied the SoupX R package (v1.6.1) with the default settings, and DoubletFinder R package (v2.0.3) with the default settings was used to remove candidate doublets. The quality control and normalization procedures of the scRNA‐seq data were conducted with Seurat R package (v4.3.0).

From the filtered cells, the gene expression matrices were normalized to the total UMI counts per cell and transformed to the natural log scale. FindVariableFeatures function was used to obtain the top 2000 highly variable genes (HVGs) from the corrected expression matrix, and then centered and scaled them after regressing cell cycle (S and G2M scores were calculated by the CellCycleScoring function in Seurat). Principle component analysis (PCA) was performed on the HVGs using RunPCA function. Dimensionality reduction and k‐nearest neighbor graphs (k = 20) of the cells were performed with the FindNeighbors function based on the Euclidean distance in the 30‐dimensional PC space; the main cell cluster was identified using the Louvain‐Jaccard graph‐based method. For classifying all filtered cells, the clustering resolution was set to 1.16 with the function FindClusters in Seurat. For visualization, RunUMAP in Seurat was used to reduce high‐dimension into two‐dimension (2D). FindAllMarkers function with the default parameters was used to identify the genes specifically expressed in each cluster. Malignant and nonmalignant cells were separated using the R package CopyKat (v1.1.0) [32]. To calculate the sample composition based on cell type, the number of cells for each cell type from each sample was counted. The counts were then divided by the total number of cells for each sample and scaled to 100% for each cell type.

Normalized enrichment scores for selected numbers of GSEA were performed by R package clusterProfiler (v4.2.2) using MSigDB Jallmark with a hyper‐geometrical statistical test with a threshold of 0.05, and the Benjamini–Hochberg method was used to estimate the FDR. The background in data was all the genes listed in the database of org.Hs.eg.db.

### 4.8 Dataset Analysis

Publicly available single‐cell RNA sequencing data from GSE146100 [33] and GSE207422 [34] were reanalyzed. GSE146100 comprises scRNA‐seq data obtained from a single patient with three early‐stage multiple primary lung cancer (MPLC) nodules. After three cycles of neoadjuvant pembrolizumab, there was remarkable tumor shrinkage in one solid nodule (W2), but no response was observed in another two subsolid nodules (W1 and W3). For GSE207422, post‐treatments tumor samples from six lung adenocarcinoma patients who received neoadjuvant immunotherapy (anti‐PD‐1 or anti‐PD‐L1) were included (Responder: BD_immune06, BD_immune11; Nonresponders: BD_immune02, BD_immune04, BD_immune12, BD_immune15). The data of malignant cells in the dataset was selected and further clustered, Uniform Manifold Approximation and Projection (UMAP) reduction, and visualization of clusters was then performed. To calculate the DTP score, the expressions of five core DTP marker genes (*CXCL8, ICAM1*, *IL6*, and *LCN2*) [20, 35, 36, 37] and four additional DTP marker genes (*CSF2, CXCL1, HNRNPAB*, and *TNFAIP3*) [38, 39, 40] were selected. The weighting of the score was based on a 2:1 ratio, with core genes given twice the weight of the additional genes.

### 4.9 In Vitro Cell Migration Assay

CD4^+^ and CD8^+^ T cells were isolated from PBMCs of three healthy donors using the EasySep Human CD4^+^ (cat. #17952) or CD8^+^ (cat. #17952) T Cell Isolation Kit (STEMCELL Technologies, Vancouver, Canada) according to the manufacturer's protocol. Isolated T cells were cultured in R10 medium supplemented with IL‐2 (100 IU/mL, cat. #200‐02, Thermo Fisher Scientific) and ImmunoCult Human CD3/CD28 T Cell Activator (cat. #10971, STEMCELL Technologies) for 3 days. H1975 cells at 80% confluence were transiently transfected with CCL20‐specific small interfering RNA (siRNA) (hs.Ri.CCL20.13.1, TriFECTa DsiRNA kit, 50 nM) or negative control siRNA (Integrated DNA Technologies, Coralville, IA, USA) using Lipofectamine RNAiMAX (Thermo Fisher Scientific) in serum‐free medium for 24 h. The conditioned medium was then transferred to the lower compartment of a well containing a Transwell insert. CD4^+^ or CD8^+^ T cells (1 × 10^5^ cells per insert) were seeded into the upper insert. After 24 h, migrated cells in the lower compartment were harvested and stained with PE‐anti‐CD4 (cat. #344604, BioLegend, San Diego, CA, USA) or APC‐anti‐CD8 (cat. #344722, BioLegend) for 30 min in the dark at 4°C. Cells were then washed and fixed with Fix/Perm buffer from the Transcription Factor Buffer Set (cat. #562574, BD Pharmingen, San Diego, CA, USA) at 4°C for 50 min. After washing, cells were stained with PE‐anti‐FoxP3 (cat. #320108, BioLegend) or PE‐anti‐CD107a (cat. #555801, BD Pharmingen) for 50 min in the dark at 4°C. Finally, cells were washed and analyzed by flow cytometry on a CytoFLEX flow cytometer, and data were analyzed using CytExpert software (Beckman Coulter Life Sciences, Brea, CA, USA).

### 4.10 Statistical Analysis

Multiple group comparisons were performed with one‐way ANOVA followed by Tukey's post hoc analyses. GraphPad Prism version 6.01 (GraphPad Software, Boston, MA, USA) was used for all statistical analyses. A *p*‐value of less than 0.05 was considered statistically significant.

## Author Contributions

Conceptualization: H.H.K., J.Y., and D.C.L.L. Data curation: N.C.M.L. and J.D. Formal analysis: H.H.K. and J.Y. Funding acquisition: D.C.L.L. Investigation: H.H.K. and J.Y. Methodology: H.H.K., J.Y., and D.C.L.L. Project administration: H.H.K. and D.C.L.L. Resources: D.C.L.L. Software: H.H.K. and J.D. Supervision: D.C.L.L. Writing – original draft preparation: H.H.K., J.Y., and D.C.L.L. Writing – review editing: All authors. All authors have read and approved the final manuscript.

## Conflicts of Interest

The authors declare no conflicts of interest.

## Funding

This work was supported by The Lee and The Ho Families Respiratory Research Fund.

## Ethics Statement

The study was conducted in accordance with the Declaration of Helsinki and its subsequent amendments. Animal experiments in this study were approved by the Committee on the Use of Live Animals in Teaching and Research (CULATR) at The University of Hong Kong and were conducted in compliance with the Animals (Control of Experiments) Ordinance of Hong Kong (CULATR‐22‐041). Collection and use of human samples were approved by the Ethics Committee of The University of Hong Kong and by the Hong Kong West Cluster Institutional Review Board of the Hong Kong Hospital Authority (IRB Reference Number UW 16–104). Informed consent was obtained from all participants prior to sample collection.

## Supporting information



Supporting Information: mco270888‐supp‐0001‐SuppMat.docx

## Data Availability

Raw data of single‐cell transcriptomic analysis have been deposited in Gene Expression Omnibus under the accession number GSE293914.
