# Supplementary material for: CCL20 Released by Drug–Tolerant Persisters Impairs Immunotherapy in EGFR–Mutant Lung Adenocarcinoma
Source: MedComm (2020). 2026 Aug 3;7(8):e70888. doi: 10.1002/mco2.70888 (PMC13430502; doi:10.1002/mco2.70888)
Supplement: Supplementary file 1 — Supporting Information: mco270888‐supp‐0001‐SuppMat.docx [file MCO2-7-e70888-s001.docx]

**CCL20 Released by Drug-Tolerant Persisters Impairs Immunotherapy in *EGFR-*mutant Lung Adenocarcinoma**

Running title: CCL20 from DTPs impairs immunotherapy

Hoi-Hin Kwok^1^, Jiashuang Yang^1^, Nerissa Chui-Mei Lee^1^, Junyang Deng^1^, David Chi-Leung Lam^1,*^

^1^Department of Medicine, Li Ka Shing Faculty of Medicine, University of Hong Kong, Hong Kong SAR, China

^*^Correspondence:

David Chi-Leung Lam, Department of Medicine, Li Ka Shing Faculty of Medicine, University of Hong Kong, Hong Kong SAR, China; Email: [dcllam@hku.hk](mailto:dcllam@hku.hk); Tel.: +852-2255-6208; Fax: +852-2872-5828


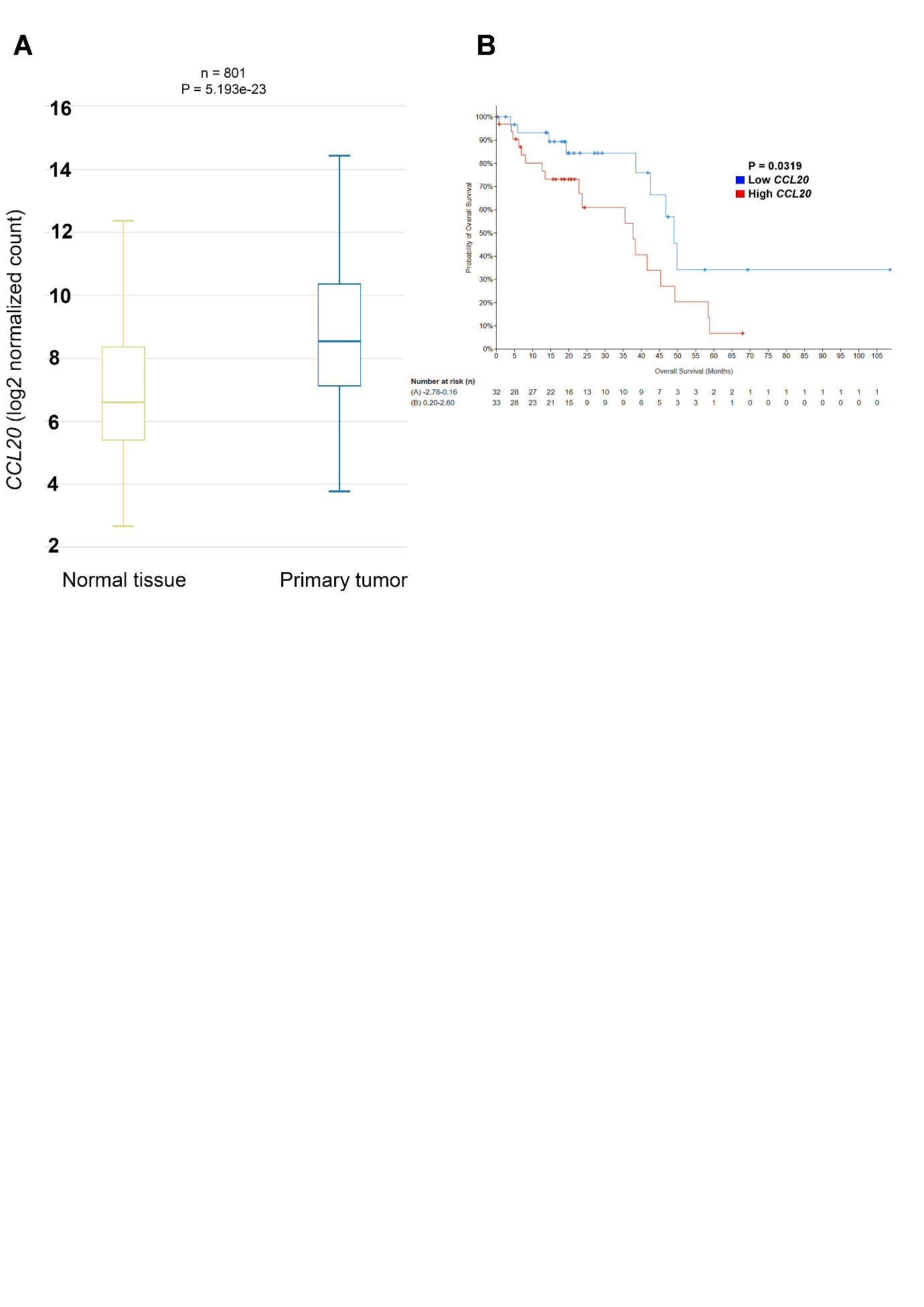


**Figure S1. CCL20 expression is upregulated in primary lung adenocarcinoma and associated with poor overall survival.**

(A) Box plot of CCL20 mRNA expression (log2 normalized count) in normal lung tissue versus primary tumors from the TCGA-LUAD cohort (n = 801; Wilcoxon rank-sum test, P = 5.193e–23). (B) Kaplan–Meier overall survival curves stratified by high vs low CCL20 expression (median cutoff; log-rank test, P = 0.0319).


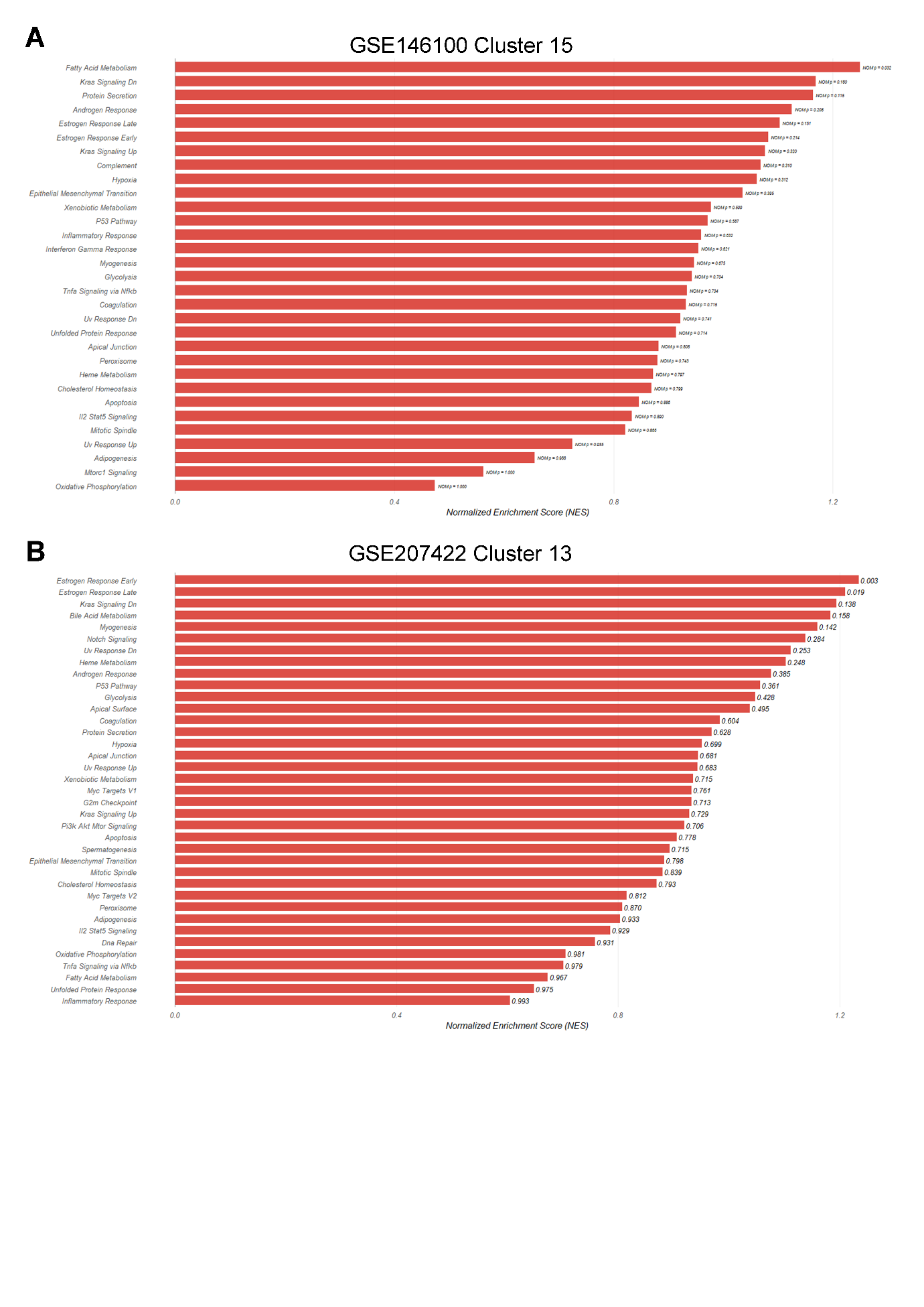


**Figure S2.** **Gene set enrichment analysis of high-DTP score clusters in public datasets.**

(A) Normalized enrichment scores for hallmark pathways in cluster 15 from GSE146100. (B) Normalized enrichment scores for hallmark pathways in cluster 13 from GSE207422.


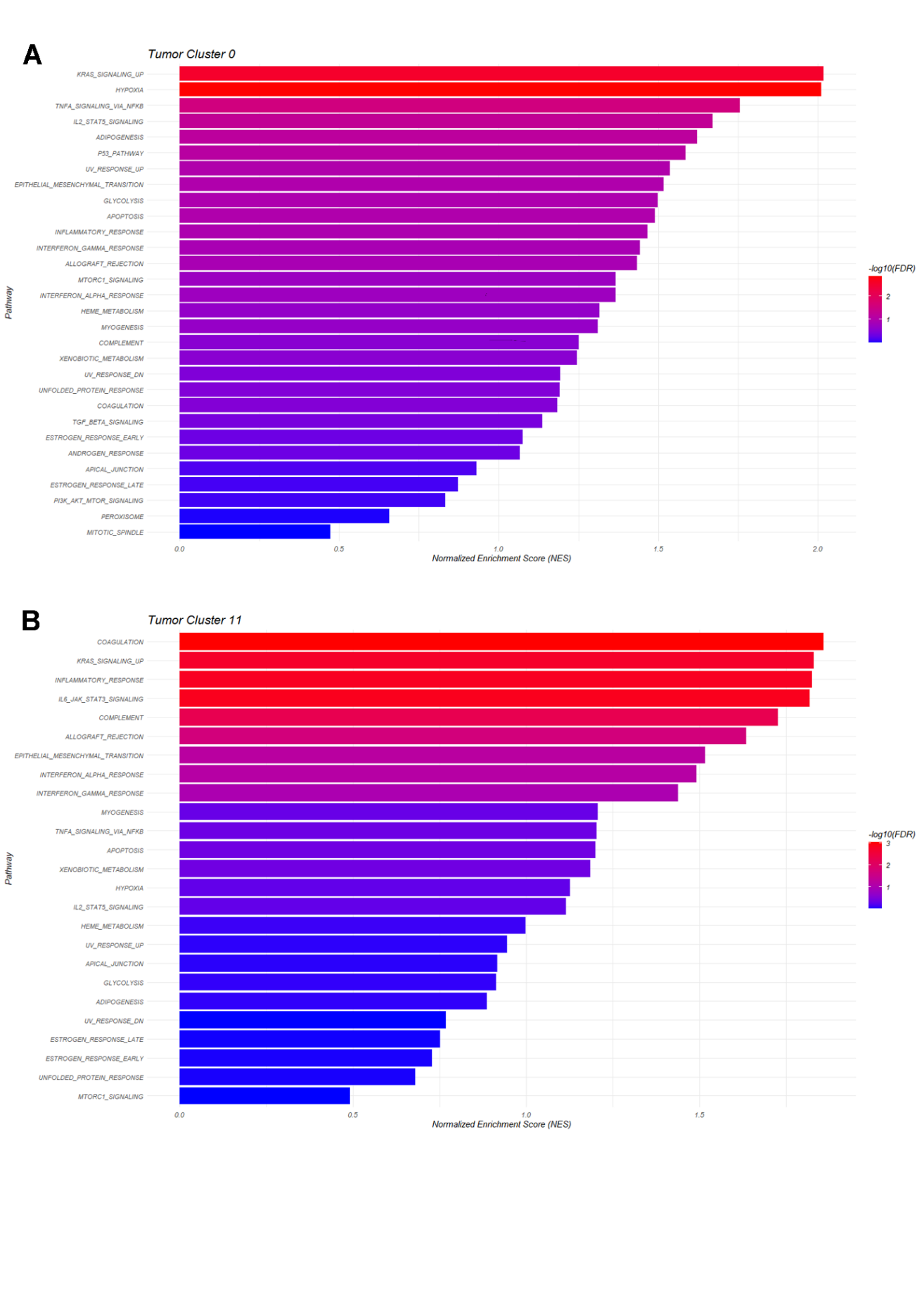


**Figure S3.** **Gene set enrichment analysis of DTP clusters in the xenograft model.**

(A) Normalized enrichment scores for hallmark pathways in cluster 0. (B) Normalized enrichment scores for hallmark pathways in cluster 11.


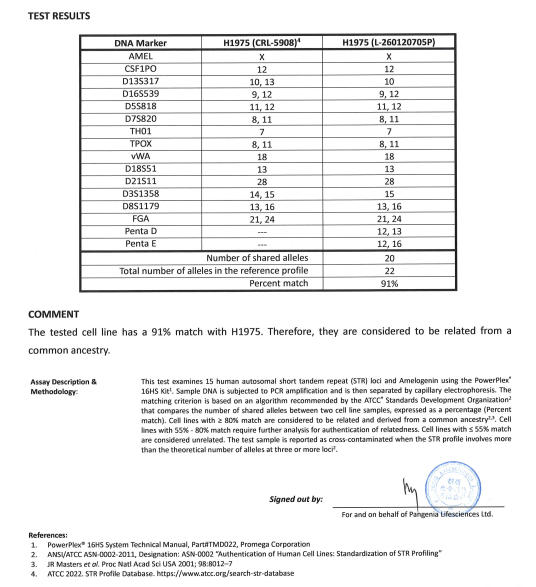


**Figure S4. Authentication result of the cell line H1975 by short tandem repeat profiling.**

| **Sample** | **Cell_Type_A** | **Cell_Type_B** | **ASI** | **Delta_a** | **P_value** |
| --- | --- | --- | --- | --- | --- |
| 1 | Basophils | Total tumor cells | 1.45 | 10793453 | 0.998 |
| 1 | CD4+ T cells | Total tumor cells | 1.73 | 18303378 | 0.998 |
| 1 | Central memory CD4+ T cells | Total tumor cells | 1.84 | 21272902 | 0.998 |
| 1 | Central memory CD8+ T cells | Total tumor cells | 1.30 | 4807976 | 0.998 |
| 1 | Cyctotoxic NK cells | Total tumor cells | 1.21 | 4499710 | 0.998 |
| 1 | Cytotoxic CD4+ T cells | Total tumor cells | 1.32 | 8832767 | 0.986 |
| 1 | Cytotoxic CD8+ T cells | Total tumor cells | 1.21 | 3190438 | 0.998 |
| 1 | Effector memory CD4+ T cells | Total tumor cells | 1.69 | 17321670 | 0.998 |
| 1 | Effector memory CD8+ T cells | Total tumor cells | 1.44 | 8704382 | 0.998 |
| 1 | Exhausted CD4+ T cells | Total tumor cells | 1.58 | 16519334 | 0.971 |
| 1 | Exhausted CD8+ T cells | Total tumor cells | 1.24 | 3955268 | 0.998 |
| 1 | Immature NK cells | Total tumor cells | 0.87 | -1492407 | 0.983 |
| 1 | M2-like macrophages | Total tumor cells | 0.70 | -6644640 | 0.856 |
| 1 | Macrophages | Total tumor cells | 1.55 | 13153454 | 0.998 |
| 1 | Memory Treg cells | Total tumor cells | 1.21 | 4019048 | 0.998 |
| 1 | Myeloid cells | Total tumor cells | 1.40 | 9898180 | 0.998 |
| 1 | Naive B cells | Total tumor cells | 0.83 | -2658266 | 0.986 |
| 1 | Naive Treg cells | Total tumor cells | 1.36 | 7733704 | 0.998 |
| 1 | NKT cells | Total tumor cells | 1.27 | 6639143 | 0.998 |
| 1 | Tfh cells | Total tumor cells | 1.74 | 19144509 | 0.998 |
| 1 | Th1 cells | Total tumor cells | 1.61 | 16051944 | 0.998 |
| 1 | Th17 cells | Total tumor cells | 1.97 | 23544746 | 0.998 |
| 2 | CD4+ T cells | Total tumor cells | 1.63 | 4058536 | 0.998 |
| 2 | CD8+ T cells | Total tumor cells | 1.23 | 1760188 | 0.998 |
| 2 | Central memory CD4+ T cells | Total tumor cells | 1.62 | 4870117 | 0.998 |
| 2 | Central memory CD8+ T cells | Total tumor cells | 1.15 | 1600024 | 0.998 |
| 2 | Cyctotoxic NK cells | Total tumor cells | 1.62 | 4113211 | 0.998 |
| 2 | Cytotoxic CD4+ T cells | Total tumor cells | 1.60 | 4030956 | 0.998 |
| 2 | Cytotoxic CD8+ T cells | Total tumor cells | 1.11 | 1147092 | 0.998 |
| 2 | Dendritic cells | Total tumor cells | 1.87 | 5279125 | 0.914 |
| 2 | Effector CD4+ T cells | Total tumor cells | 1.63 | 4277701 | 0.965 |
| 2 | Effector CD8+ T cells | Total tumor cells | 1.30 | 2400521 | 0.986 |
| 2 | Effector memory CD4+ T cells | Total tumor cells | 1.63 | 3925214 | 0.998 |
| 2 | Effector memory CD8+ T cells | Total tumor cells | 1.24 | 1789896 | 0.998 |
| 2 | Exhausted CD4+ T cells | Total tumor cells | 1.64 | 4662123 | 0.998 |
| 2 | Exhausted CD8+ T cells | Total tumor cells | 1.70 | 4924043 | 0.998 |
| 2 | Immature NK cells | Total tumor cells | 0.72 | -1573630 | 0.751 |
| 2 | M2-like macrophages | Total tumor cells | 1.49 | 3352773 | 0.998 |
| 2 | Macrophages | Total tumor cells | 1.41 | 2526980 | 0.998 |
| 2 | Mast Cells | Total tumor cells | 1.81 | 4613949 | 0.998 |
| 2 | Memory Treg cells | Total tumor cells | 1.58 | 4064173 | 0.998 |
| 2 | Myeloid cells | Total tumor cells | 1.33 | 2048441 | 0.998 |
| 2 | Naive B cells | Total tumor cells | 1.19 | 1314450 | 0.920 |
| 2 | Naive Treg cells | Total tumor cells | 1.56 | 4035800 | 0.998 |
| 2 | NKT cells | Total tumor cells | 1.46 | 2779020 | 0.955 |
| 2 | Tfh cells | Total tumor cells | 1.65 | 5444169 | 0.973 |
| 2 | Th1 cells | Total tumor cells | 1.68 | 4413169 | 0.998 |
| 2 | Th17 cells | Total tumor cells | 1.72 | 3864281 | 0.992 |
| 2 | Th2 cells | Total tumor cells | 1.46 | 2957342 | 0.998 |
| 3 | Basophils | Total tumor cells | 1.24 | 2040298 | 0.998 |
| 3 | CD4+ T cells | Total tumor cells | 1.17 | 1404378 | 0.998 |
| 3 | CD8+ T cells | Total tumor cells | 1.36 | 2804818 | 0.998 |
| 3 | Central memory CD4+ T cells | Total tumor cells | 1.22 | 1620489 | 0.998 |
| 3 | Central memory CD8+ T cells | Total tumor cells | 1.48 | 3595771 | 0.998 |
| 3 | Cyctotoxic NK cells | Total tumor cells | 1.25 | 2010974 | 0.998 |
| 3 | Cytotoxic CD4+ T cells | Total tumor cells | 1.17 | 1419590 | 0.998 |
| 3 | Cytotoxic CD8+ T cells | Total tumor cells | 1.37 | 2866279 | 0.998 |
| 3 | Effector CD4+ T cells | Total tumor cells | 1.22 | 1620489 | 0.998 |
| 3 | Effector CD8+ T cells | Total tumor cells | 1.10 | 1118517 | 0.998 |
| 3 | Effector memory CD4+ T cells | Total tumor cells | 1.18 | 1351475 | 0.998 |
| 3 | Effector memory CD8+ T cells | Total tumor cells | 1.20 | 1853774 | 0.998 |
| 3 | Exhausted CD4+ T cells | Total tumor cells | 1.11 | 1073338 | 0.998 |
| 3 | Exhausted CD8+ T cells | Total tumor cells | 1.36 | 2824096 | 0.998 |
| 3 | gdT cells | Total tumor cells | 2.18 | 9427667 | 0.998 |
| 3 | Immature NK cells | Total tumor cells | 1.18 | 1022544 | 0.998 |
| 3 | M2-like macrophages | Total tumor cells | 1.31 | 2641615 | 0.998 |
| 3 | Macrophages | Total tumor cells | 1.31 | 2641615 | 0.998 |
| 3 | Memory Treg cells | Total tumor cells | 1.62 | 5092986 | 0.998 |
| 3 | Myeloid cells | Total tumor cells | 1.43 | 3485519 | 0.998 |
| 3 | Naive B cells | Total tumor cells | 1.27 | 2331169 | 0.998 |
| 3 | Naive CD4+ T cells | Total tumor cells | 1.15 | 1369283 | 0.998 |
| 3 | Naive CD8+ T cells | Total tumor cells | 1.18 | 1605547 | 0.998 |
| 3 | Neutrophils | Total tumor cells | 1.14 | 1173663 | 0.998 |
| 3 | NKT cells | Total tumor cells | 1.43 | 3263972 | 0.998 |
| 3 | Tfh cells | Total tumor cells | 1.04 | 590082 | 0.998 |
| 3 | Th1 cells | Total tumor cells | 1.21 | 1467233 | 0.998 |
| 4 | Basophils | Total tumor cells | 1.02 | 1003789 | 0.998 |
| 4 | CD4+ T cells | Total tumor cells | 1.40 | 4530765 | 0.998 |
| 4 | CD8+ T cells | Total tumor cells | 1.68 | 5624281 | 0.998 |
| 4 | Central memory CD4+ T cells | Total tumor cells | 1.44 | 4721841 | 0.998 |
| 4 | Central memory CD8+ T cells | Total tumor cells | 1.71 | 6004366 | 0.998 |
| 4 | Cyctotoxic NK cells | Total tumor cells | 1.18 | 1910279 | 0.998 |
| 4 | Cyctotoxic NK cells_2 | Total tumor cells | 1.18 | 1910279 | 0.998 |
| 4 | Cytotoxic CD4+ T cells | Total tumor cells | 1.14 | 1960807 | 0.998 |
| 4 | Cytotoxic CD8+ T cells | Total tumor cells | 1.27 | 2889543 | 0.998 |
| 4 | Dendritic cells | Total tumor cells | 1.16 | 2611229 | 0.998 |
| 4 | Effector CD8+ T cells | Total tumor cells | 1.20 | 2125174 | 0.998 |
| 4 | Effector memory CD4+ T cells | Total tumor cells | 1.55 | 4489925 | 0.998 |
| 4 | Effector memory CD8+ T cells | Total tumor cells | 1.69 | 5349451 | 0.998 |
| 4 | Exhausted CD4+ T cells | Total tumor cells | 1.40 | 4159690 | 0.998 |
| 4 | Exhausted CD8+ T cells | Total tumor cells | 1.70 | 5686132 | 0.998 |
| 4 | Immature NK cells | Total tumor cells | 1.14 | 1490079 | 0.998 |
| 4 | M1-like macrophages | Total tumor cells | 1.57 | 5379513 | 0.998 |
| 4 | M2-like macrophages | Total tumor cells | 1.25 | 3440870 | 0.998 |
| 4 | Macrophages | Total tumor cells | 1.07 | 1597833 | 0.998 |
| 4 | Mast Cells | Total tumor cells | 1.19 | 2462033 | 0.998 |
| 4 | Memory Treg cells | Total tumor cells | 1.62 | 6750280 | 0.998 |
| 4 | Myeloid cells | Total tumor cells | 1.06 | 1357176 | 0.998 |
| 4 | Naive B cells | Total tumor cells | 1.38 | 3494015 | 0.998 |
| 4 | Naive CD4+ T cells | Total tumor cells | 1.34 | 4315151 | 0.998 |
| 4 | Naive CD8+ T cells | Total tumor cells | 1.32 | 3853620 | 0.998 |
| 4 | Naive Treg cells | Total tumor cells | 1.22 | 5518691 | 0.979 |
| 4 | Neutrophils | Total tumor cells | 0.93 | 226610 | 0.963 |
| 4 | NKT cells | Total tumor cells | 1.48 | 4431971 | 0.998 |
| 4 | Plasmacytoid DCs | Total tumor cells | 1.27 | 3673371 | 0.998 |
| 4 | Tfh cells | Total tumor cells | 1.60 | 7556294 | 0.998 |
| 2 | CD4+ T cells | Th2 cells | 5.94 | 339936404 | 0.998 |
| 2 | CD8+ T cells | Th2 cells | 1.85 | 48879983 | 0.994 |
| 2 | Central memory CD4+ T cells | Th2 cells | 5.42 | 204568848 | 0.998 |
| 2 | Central memory CD8+ T cells | Th2 cells | 2.63 | 60356716 | 0.932 |
| 2 | Cyctotoxic NK cells | Th2 cells | 4.47 | 232573550 | 0.992 |
| 2 | Cytotoxic CD4+ T cells | Th2 cells | 5.85 | 332896154 | 0.998 |
| 2 | Cytotoxic CD8+ T cells | Th2 cells | 1.85 | 46809591 | 0.992 |
| 2 | Dendritic cells | Th2 cells | 5.47 | 141512790 | 0.511 |
| 2 | Effector CD4+ T cells | Th2 cells | 8.14 | 293077602 | 0.655 |
| 2 | Effector CD8+ T cells | Th2 cells | 4.28 | 195869468 | 0.905 |
| 2 | Effector memory CD4+ T cells | Th2 cells | 6.02 | 362522972 | 0.998 |
| 2 | Effector memory CD8+ T cells | Th2 cells | 1.72 | 46918478 | 0.996 |
| 2 | Exhausted CD4+ T cells | Th2 cells | 6.32 | 284454169 | 0.998 |
| 2 | Exhausted CD8+ T cells | Th2 cells | 3.98 | 126309395 | 0.990 |
| 2 | Immature NK cells | Th2 cells | 2.53 | 48029207 | 0.524 |
| 2 | M2-like macrophages | Th2 cells | 4.65 | 231576685 | 0.963 |
| 2 | Macrophages | Th2 cells | 3.33 | 145456593 | 0.994 |
| 2 | Mast Cells | Th2 cells | 2.00 | 48333507 | 0.914 |
| 2 | Memory Treg cells | Th2 cells | 1.36 | -15899823 | 0.920 |
| 2 | Myeloid cells | Th2 cells | 3.05 | 121567630 | 0.994 |
| 2 | Naive B cells | Th2 cells | 3.47 | 134320317 | 0.984 |
| 2 | Naive Treg cells | Th2 cells | 0.96 | -27508661 | 0.918 |
| 2 | NKT cells | Th2 cells | 4.78 | 184547456 | 0.735 |
| 2 | Tfh cells | Th2 cells | 4.74 | 59358262 | 0.310 |
| 2 | Th1 cells | Th2 cells | 5.95 | 313615645 | 0.992 |
| 2 | Th17 cells | Th2 cells | 7.42 | 379919739 | 0.998 |
| 1 | Basophils | Th17 cells | 6.19 | 445300268 | 0.994 |
| 1 | CD4+ T cells | Th17 cells | 7.84 | 698819375 | 0.998 |
| 1 | Central memory CD4+ T cells | Th17 cells | 7.39 | 756824866 | 0.998 |
| 1 | Central memory CD8+ T cells | Th17 cells | 0.17 | -30718395 | 0.981 |
| 1 | Cyctotoxic NK cells | Th17 cells | 1.60 | -1211493 | 0.979 |
| 1 | Cytotoxic CD4+ T cells | Th17 cells | 8.95 | 489771411 | 0.949 |
| 1 | Cytotoxic CD8+ T cells | Th17 cells | 0.00 | -50421325 | 0.981 |
| 1 | Effector memory CD4+ T cells | Th17 cells | 8.00 | 680365478 | 0.998 |
| 1 | Effector memory CD8+ T cells | Th17 cells | 0.29 | -17021519 | 0.975 |
| 1 | Exhausted CD4+ T cells | Th17 cells | 0.00 | -50421325 | 0.483 |
| 1 | Exhausted CD8+ T cells | Th17 cells | 0.00 | -50421325 | 0.992 |
| 1 | Immature NK cells | Th17 cells | 0.33 | -29106922 | 0.961 |
| 1 | M2-like macrophages | Th17 cells | 5.50 | 161363261 | 0.721 |
| 1 | Macrophages | Th17 cells | 7.26 | 562567017 | 0.990 |
| 1 | Memory Treg cells | Th17 cells | 0.00 | -50421325 | 0.953 |
| 1 | Myeloid cells | Th17 cells | 5.68 | 418618196 | 0.992 |
| 1 | Naive B cells | Th17 cells | 0.00 | -50421325 | 0.975 |
| 1 | Naive Treg cells | Th17 cells | 0.00 | -50421325 | 0.906 |
| 1 | NKT cells | Th17 cells | 1.35 | 58391628 | 0.998 |
| 1 | Tfh cells | Th17 cells | 6.38 | 648158049 | 0.998 |
| 1 | Th1 cells | Th17 cells | 6.79 | 594014072 | 0.998 |
| 2 | CD4+ T cells | Th17 cells | 5.89 | 369223920 | 0.998 |
| 2 | CD8+ T cells | Th17 cells | 1.38 | 25139403 | 0.940 |
| 2 | Central memory CD4+ T cells | Th17 cells | 6.77 | 200786722 | 0.938 |
| 2 | Central memory CD8+ T cells | Th17 cells | 2.33 | 30405516 | 0.866 |
| 2 | Cyctotoxic NK cells | Th17 cells | 4.08 | 277818882 | 0.961 |
| 2 | Cytotoxic CD4+ T cells | Th17 cells | 6.17 | 345104752 | 0.990 |
| 2 | Cytotoxic CD8+ T cells | Th17 cells | 1.38 | 21193821 | 0.918 |
| 2 | Dendritic cells | Th17 cells | 6.85 | 307351118 | 0.643 |
| 2 | Effector CD4+ T cells | Th17 cells | 10.95 | 681129055 | 0.530 |
| 2 | Effector CD8+ T cells | Th17 cells | 4.43 | 231990604 | 0.524 |
| 2 | Effector memory CD4+ T cells | Th17 cells | 5.74 | 395931007 | 0.998 |
| 2 | Effector memory CD8+ T cells | Th17 cells | 1.21 | 24209771 | 0.967 |
| 2 | Exhausted CD4+ T cells | Th17 cells | 6.94 | 366243666 | 0.981 |
| 2 | Exhausted CD8+ T cells | Th17 cells | 3.87 | 138103541 | 0.961 |
| 2 | Immature NK cells | Th17 cells | 2.26 | 47096136 | 0.000 |
| 2 | M2-like macrophages | Th17 cells | 4.42 | 265620154 | 0.883 |
| 2 | Macrophages | Th17 cells | 2.96 | 123975732 | 0.983 |
| 2 | Mast Cells | Th17 cells | 1.75 | 82982928 | 0.858 |
| 2 | Memory Treg cells | Th17 cells | 0.69 | -15053775 | 0.448 |
| 2 | Myeloid cells | Th17 cells | 2.71 | 103772805 | 0.990 |
| 2 | Naive B cells | Th17 cells | 3.04 | 144424219 | 0.910 |
| 2 | Naive Treg cells | Th17 cells | 0.32 | -37061727 | 0.834 |
| 2 | NKT cells | Th17 cells | 5.74 | 268172221 | 0.472 |
| 2 | Tfh cells | Th17 cells | 8.41 | 130528114 | 0.222 |
| 2 | Th1 cells | Th17 cells | 6.09 | 380087821 | 0.990 |
| 1 | Basophils | Th1 cells | 5.53 | 514578212 | 0.994 |
| 1 | CD4+ T cells | Th1 cells | 8.42 | 795033352 | 0.998 |
| 1 | Central memory CD4+ T cells | Th1 cells | 9.13 | 838381720 | 0.998 |
| 1 | Central memory CD8+ T cells | Th1 cells | 0.27 | -26625133 | 0.891 |
| 1 | Cyctotoxic NK cells | Th1 cells | 1.56 | 7797076 | 0.969 |
| 1 | Cytotoxic CD4+ T cells | Th1 cells | 7.56 | 655425175 | 0.971 |
| 1 | Cytotoxic CD8+ T cells | Th1 cells | 0.02 | -49118985 | 0.975 |
| 1 | Effector memory CD4+ T cells | Th1 cells | 8.19 | 781224192 | 0.998 |
| 1 | Effector memory CD8+ T cells | Th1 cells | 0.37 | -16534709 | 0.928 |
| 1 | Exhausted CD4+ T cells | Th1 cells | 0.08 | -39089223 | 0.287 |
| 1 | Exhausted CD8+ T cells | Th1 cells | 0.07 | -45872905 | 0.984 |
| 1 | Immature NK cells | Th1 cells | 0.36 | -30426353 | 0.959 |
| 1 | M2-like macrophages | Th1 cells | 4.32 | 221340938 | 0.513 |
| 1 | Macrophages | Th1 cells | 6.35 | 631882752 | 0.990 |
| 1 | Memory Treg cells | Th1 cells | 0.06 | -46082873 | 0.867 |
| 1 | Myeloid cells | Th1 cells | 5.15 | 477312233 | 0.992 |
| 1 | Naive B cells | Th1 cells | 0.00 | -50421325 | 0.967 |
| 1 | Naive Treg cells | Th1 cells | 0.06 | -44490790 | 0.791 |
| 1 | NKT cells | Th1 cells | 1.47 | 73368656 | 0.998 |
| 1 | Tfh cells | Th1 cells | 7.29 | 710995983 | 0.998 |
| 2 | CD4+ T cells | Th1 cells | 5.97 | 361521979 | 0.998 |
| 2 | CD8+ T cells | Th1 cells | 1.75 | 45795490 | 0.998 |
| 2 | Central memory CD4+ T cells | Th1 cells | 6.36 | 334651398 | 0.998 |
| 2 | Central memory CD8+ T cells | Th1 cells | 2.46 | 87135652 | 0.998 |
| 2 | Cyctotoxic NK cells | Th1 cells | 5.19 | 292102784 | 0.994 |
| 2 | Cytotoxic CD4+ T cells | Th1 cells | 6.04 | 357685965 | 0.998 |
| 2 | Cytotoxic CD8+ T cells | Th1 cells | 1.66 | 40279638 | 0.998 |
| 2 | Dendritic cells | Th1 cells | 7.37 | 415713750 | 0.986 |
| 2 | Effector CD4+ T cells | Th1 cells | 8.69 | 300922361 | 0.846 |
| 2 | Effector CD8+ T cells | Th1 cells | 3.95 | 117006325 | 0.735 |
| 2 | Effector memory CD4+ T cells | Th1 cells | 5.90 | 365642638 | 0.998 |
| 2 | Effector memory CD8+ T cells | Th1 cells | 1.62 | 38705708 | 0.998 |
| 2 | Exhausted CD4+ T cells | Th1 cells | 6.88 | 390191964 | 0.998 |
| 2 | Exhausted CD8+ T cells | Th1 cells | 4.56 | 207490676 | 0.998 |
| 2 | Immature NK cells | Th1 cells | 1.90 | 13589097 | 0.754 |
| 2 | M2-like macrophages | Th1 cells | 4.85 | 278490602 | 0.994 |
| 2 | Macrophages | Th1 cells | 3.78 | 171951249 | 0.996 |
| 2 | Mast Cells | Th1 cells | 4.34 | 189400056 | 0.994 |
| 2 | Memory Treg cells | Th1 cells | 2.24 | 41942251 | 0.998 |
| 2 | Myeloid cells | Th1 cells | 3.43 | 148157301 | 0.996 |
| 2 | Naive B cells | Th1 cells | 3.31 | 126868423 | 0.979 |
| 2 | Naive Treg cells | Th1 cells | 1.81 | 20753160 | 0.998 |
| 2 | NKT cells | Th1 cells | 4.95 | 159848399 | 0.875 |
| 2 | Tfh cells | Th1 cells | 7.43 | 359132343 | 0.994 |
| 3 | Basophils | Th1 cells | 2.67 | 179122659 | 0.986 |
| 3 | CD4+ T cells | Th1 cells | 3.51 | 272183357 | 0.998 |
| 3 | CD8+ T cells | Th1 cells | 2.11 | 45498575 | 0.996 |
| 3 | Central memory CD4+ T cells | Th1 cells | 3.41 | 288380846 | 0.998 |
| 3 | Central memory CD8+ T cells | Th1 cells | 1.82 | 17465536 | 0.458 |
| 3 | Cyctotoxic NK cells | Th1 cells | 3.03 | 102025088 | 0.988 |
| 3 | Cytotoxic CD4+ T cells | Th1 cells | 3.56 | 273603539 | 0.998 |
| 3 | Cytotoxic CD8+ T cells | Th1 cells | 2.14 | 45759384 | 0.984 |
| 3 | Effector CD4+ T cells | Th1 cells | 3.41 | 288380846 | 0.998 |
| 3 | Effector CD8+ T cells | Th1 cells | 1.88 | 73467050 | 0.743 |
| 3 | Effector memory CD4+ T cells | Th1 cells | 3.17 | 282026165 | 0.998 |
| 3 | Effector memory CD8+ T cells | Th1 cells | 2.02 | 42813118 | 0.632 |
| 3 | Exhausted CD4+ T cells | Th1 cells | 3.36 | 220968071 | 0.998 |
| 3 | Exhausted CD8+ T cells | Th1 cells | 2.39 | 56753985 | 0.998 |
| 3 | gdT cells | Th1 cells | 0.22 | -40019203 | 0.903 |
| 3 | Immature NK cells | Th1 cells | 1.86 | 44814618 | 0.871 |
| 3 | M2-like macrophages | Th1 cells | 1.67 | 59890298 | 0.936 |
| 3 | Macrophages | Th1 cells | 1.67 | 59890298 | 0.936 |
| 3 | Memory Treg cells | Th1 cells | 1.97 | 89073287 | 0.988 |
| 3 | Myeloid cells | Th1 cells | 2.44 | 129696054 | 0.984 |
| 3 | Naive B cells | Th1 cells | 1.40 | 288617 | 0.891 |
| 3 | Naive CD4+ T cells | Th1 cells | 3.65 | 270921538 | 0.998 |
| 3 | Naive CD8+ T cells | Th1 cells | 2.76 | 99867862 | 0.994 |
| 3 | Neutrophils | Th1 cells | 3.12 | 245371254 | 0.986 |
| 3 | NKT cells | Th1 cells | 2.01 | 50983384 | 0.998 |
| 3 | Tfh cells | Th1 cells | 3.54 | 197267663 | 0.998 |
| 1 | Basophils | Tfh cells | 5.38 | 468948332 | 0.988 |
| 1 | CD4+ T cells | Tfh cells | 7.54 | 726319547 | 0.998 |
| 1 | Central memory CD4+ T cells | Tfh cells | 7.71 | 777713381 | 0.998 |
| 1 | Central memory CD8+ T cells | Tfh cells | 0.39 | -17792046 | 0.955 |
| 1 | Cyctotoxic NK cells | Tfh cells | 1.89 | 28152525 | 0.842 |
| 1 | Cytotoxic CD4+ T cells | Tfh cells | 7.89 | 580106598 | 0.961 |
| 1 | Cytotoxic CD8+ T cells | Tfh cells | 0.11 | -44672722 | 0.977 |
| 1 | Effector memory CD4+ T cells | Tfh cells | 7.49 | 709822267 | 0.998 |
| 1 | Effector memory CD8+ T cells | Tfh cells | 0.35 | -15723209 | 0.965 |
| 1 | Exhausted CD4+ T cells | Tfh cells | 0.35 | 1211413 | 0.409 |
| 1 | Exhausted CD8+ T cells | Tfh cells | 0.32 | -30681948 | 0.912 |
| 1 | Immature NK cells | Tfh cells | 0.34 | -29728889 | 0.895 |
| 1 | M2-like macrophages | Tfh cells | 4.39 | 187855466 | 0.499 |
| 1 | Macrophages | Tfh cells | 6.25 | 579390071 | 0.986 |
| 1 | Memory Treg cells | Tfh cells | 0.30 | -31187915 | 0.885 |
| 1 | Myeloid cells | Tfh cells | 4.99 | 436570000 | 0.988 |
| 1 | Naive B cells | Tfh cells | 0.00 | -50421325 | 0.971 |
| 1 | Naive Treg cells | Tfh cells | 0.26 | -23913480 | 0.776 |
| 1 | NKT cells | Tfh cells | 1.48 | 76649437 | 0.998 |
| 2 | CD4+ T cells | Tfh cells | 6.35 | 243194029 | 0.998 |
| 2 | CD8+ T cells | Tfh cells | 2.30 | 52094701 | 0.975 |
| 2 | Central memory CD4+ T cells | Tfh cells | 4.66 | 450666541 | 0.998 |
| 2 | Central memory CD8+ T cells | Tfh cells | 2.28 | 140695653 | 0.988 |
| 2 | Cyctotoxic NK cells | Tfh cells | 7.56 | 283360180 | 0.591 |
| 2 | Cytotoxic CD4+ T cells | Tfh cells | 6.12 | 253135594 | 0.998 |
| 2 | Cytotoxic CD8+ T cells | Tfh cells | 1.85 | 40248289 | 0.969 |
| 2 | Dendritic cells | Tfh cells | 7.30 | 702870210 | 0.856 |
| 2 | Effector CD8+ T cells | Tfh cells | 0.00 | -50496251 | 0.000 |
| 2 | Effector memory CD4+ T cells | Tfh cells | 6.63 | 208984629 | 0.723 |
| 2 | Effector memory CD8+ T cells | Tfh cells | 2.30 | 36917531 | 0.799 |
| 2 | Exhausted CD4+ T cells | Tfh cells | 7.31 | 318346207 | 0.998 |
| 2 | Exhausted CD8+ T cells | Tfh cells | 6.13 | 268767451 | 0.992 |
| 2 | Immature NK cells | Tfh cells | 0.00 | -50496251 | 0.000 |
| 2 | M2-like macrophages | Tfh cells | 6.01 | 248145053 | 0.686 |
| 2 | Macrophages | Tfh cells | 4.30 | 203907768 | 0.977 |
| 2 | Mast Cells | Tfh cells | 7.60 | 525959788 | 0.637 |
| 2 | Memory Treg cells | Tfh cells | 4.55 | 194735021 | 0.571 |
| 2 | Myeloid cells | Tfh cells | 3.90 | 180037835 | 0.977 |
| 2 | Naive B cells | Tfh cells | 1.77 | 37390963 | 0.000 |
| 2 | Naive Treg cells | Tfh cells | 4.05 | 177698684 | 0.536 |
| 2 | NKT cells | Tfh cells | 1.71 | 29254470 | 0.000 |
| 3 | Basophils | Tfh cells | 3.02 | 156515049 | 0.986 |
| 3 | CD4+ T cells | Tfh cells | 3.57 | 223077620 | 0.998 |
| 3 | CD8+ T cells | Tfh cells | 2.68 | 75545730 | 0.998 |
| 3 | Central memory CD4+ T cells | Tfh cells | 3.60 | 213021364 | 0.998 |
| 3 | Central memory CD8+ T cells | Tfh cells | 2.79 | 61258673 | 0.770 |
| 3 | Cyctotoxic NK cells | Tfh cells | 3.01 | 91022279 | 0.990 |
| 3 | Cytotoxic CD4+ T cells | Tfh cells | 3.61 | 226583883 | 0.998 |
| 3 | Cytotoxic CD8+ T cells | Tfh cells | 2.75 | 78294995 | 0.998 |
| 3 | Effector CD4+ T cells | Tfh cells | 3.60 | 213021364 | 0.998 |
| 3 | Effector CD8+ T cells | Tfh cells | 1.67 | 49290990 | 0.852 |
| 3 | Effector memory CD4+ T cells | Tfh cells | 3.73 | 209907963 | 0.983 |
| 3 | Effector memory CD8+ T cells | Tfh cells | 1.54 | 23604881 | 0.503 |
| 3 | Exhausted CD4+ T cells | Tfh cells | 2.85 | 187397975 | 0.998 |
| 3 | Exhausted CD8+ T cells | Tfh cells | 2.94 | 88065091 | 0.998 |
| 3 | gdT cells | Tfh cells | 0.22 | -39824259 | 0.875 |
| 3 | Immature NK cells | Tfh cells | 1.96 | 45904457 | 0.971 |
| 3 | M2-like macrophages | Tfh cells | 2.35 | 79957366 | 0.986 |
| 3 | Macrophages | Tfh cells | 2.35 | 79957366 | 0.986 |
| 3 | Memory Treg cells | Tfh cells | 1.54 | 70312411 | 0.998 |
| 3 | Myeloid cells | Tfh cells | 3.59 | 191384769 | 0.986 |
| 3 | Naive B cells | Tfh cells | 2.75 | 26481637 | 0.912 |
| 3 | Naive CD4+ T cells | Tfh cells | 3.56 | 230513199 | 0.998 |
| 3 | Naive CD8+ T cells | Tfh cells | 2.81 | 117518297 | 0.998 |
| 3 | Neutrophils | Tfh cells | 3.26 | 190252580 | 0.988 |
| 3 | NKT cells | Tfh cells | 2.19 | 63190012 | 0.998 |
| 4 | Basophils | Tfh cells | 2.84 | 113583191 | 0.994 |
| 4 | CD4+ T cells | Tfh cells | 1.84 | 96362339 | 0.998 |
| 4 | CD8+ T cells | Tfh cells | 0.41 | -20847727 | 0.984 |
| 4 | Central memory CD4+ T cells | Tfh cells | 2.33 | 141128660 | 0.998 |
| 4 | Central memory CD8+ T cells | Tfh cells | 0.54 | -9948270 | 0.971 |
| 4 | Cyctotoxic NK cells | Tfh cells | 1.78 | 27197986 | 0.988 |
| 4 | Cyctotoxic NK cells_2 | Tfh cells | 1.78 | 27197986 | 0.988 |
| 4 | Cytotoxic CD4+ T cells | Tfh cells | 1.10 | 21094980 | 0.998 |
| 4 | Cytotoxic CD8+ T cells | Tfh cells | 0.43 | -24447703 | 0.922 |
| 4 | Dendritic cells | Tfh cells | 4.01 | 167702053 | 0.992 |
| 4 | Effector CD8+ T cells | Tfh cells | 0.25 | -29549332 | 0.558 |
| 4 | Effector memory CD4+ T cells | Tfh cells | 0.03 | -47185281 | 0.864 |
| 4 | Effector memory CD8+ T cells | Tfh cells | 0.08 | -46081191 | 0.983 |
| 4 | Exhausted CD4+ T cells | Tfh cells | 1.63 | 82290626 | 0.998 |
| 4 | Exhausted CD8+ T cells | Tfh cells | 0.31 | -29125526 | 0.986 |
| 4 | Immature NK cells | Tfh cells | 1.30 | 26565585 | 0.994 |
| 4 | M1-like macrophages | Tfh cells | 2.47 | 206698991 | 0.994 |
| 4 | M2-like macrophages | Tfh cells | 2.98 | 215602152 | 0.994 |
| 4 | Macrophages | Tfh cells | 1.86 | 107882106 | 0.994 |
| 4 | Mast Cells | Tfh cells | 4.86 | 247424084 | 0.994 |
| 4 | Memory Treg cells | Tfh cells | 2.62 | 230719564 | 0.998 |
| 4 | Myeloid cells | Tfh cells | 3.03 | 126364797 | 0.994 |
| 4 | Naive B cells | Tfh cells | 1.23 | 24492556 | 0.992 |
| 4 | Naive CD4+ T cells | Tfh cells | 1.51 | 62621964 | 0.998 |
| 4 | Naive CD8+ T cells | Tfh cells | 1.25 | 40624056 | 0.998 |
| 4 | Naive Treg cells | Tfh cells | 2.84 | 233171203 | 0.998 |
| 4 | Neutrophils | Tfh cells | 0.58 | -18878672 | 0.914 |
| 4 | NKT cells | Tfh cells | 0.72 | 3366155 | 0.957 |
| 4 | Plasmacytoid DCs | Tfh cells | 5.09 | 223509272 | 0.992 |
| 4 | Basophils | Plasmacytoid DCs | 3.15 | 104563691 | 0.998 |
| 4 | CD4+ T cells | Plasmacytoid DCs | 2.33 | 73760695 | 0.994 |
| 4 | CD8+ T cells | Plasmacytoid DCs | 0.48 | -25805950 | 0.975 |
| 4 | Central memory CD4+ T cells | Plasmacytoid DCs | 2.61 | 86960013 | 0.994 |
| 4 | Central memory CD8+ T cells | Plasmacytoid DCs | 0.61 | -19133601 | 0.975 |
| 4 | Cyctotoxic NK cells | Plasmacytoid DCs | 1.89 | 14833604 | 0.957 |
| 4 | Cyctotoxic NK cells_2 | Plasmacytoid DCs | 1.89 | 14833605 | 0.957 |
| 4 | Cytotoxic CD4+ T cells | Plasmacytoid DCs | 1.26 | 23241129 | 0.992 |
| 4 | Cytotoxic CD8+ T cells | Plasmacytoid DCs | 0.53 | -23248614 | 0.963 |
| 4 | Dendritic cells | Plasmacytoid DCs | 4.83 | 323172989 | 0.998 |
| 4 | Effector CD8+ T cells | Plasmacytoid DCs | 0.07 | -47716385 | 0.813 |
| 4 | Effector memory CD4+ T cells | Plasmacytoid DCs | 0.17 | -47124801 | 0.963 |
| 4 | Effector memory CD8+ T cells | Plasmacytoid DCs | 0.09 | -47196517 | 0.994 |
| 4 | Exhausted CD4+ T cells | Plasmacytoid DCs | 1.83 | 42862708 | 0.994 |
| 4 | Exhausted CD8+ T cells | Plasmacytoid DCs | 0.33 | -34161811 | 0.951 |
| 4 | Immature NK cells | Plasmacytoid DCs | 1.89 | 62501808 | 0.998 |
| 4 | M1-like macrophages | Plasmacytoid DCs | 2.88 | 104775543 | 0.998 |
| 4 | M2-like macrophages | Plasmacytoid DCs | 4.04 | 134863144 | 0.998 |
| 4 | Macrophages | Plasmacytoid DCs | 2.60 | 67325219 | 0.998 |
| 4 | Mast Cells | Plasmacytoid DCs | 4.80 | 195539813 | 0.998 |
| 4 | Memory Treg cells | Plasmacytoid DCs | 3.41 | 125535188 | 0.990 |
| 4 | Myeloid cells | Plasmacytoid DCs | 3.32 | 120772921 | 0.998 |
| 4 | Naive B cells | Plasmacytoid DCs | 1.43 | 20858795 | 0.893 |
| 4 | Naive CD4+ T cells | Plasmacytoid DCs | 2.35 | 78060624 | 0.992 |
| 4 | Naive CD8+ T cells | Plasmacytoid DCs | 1.86 | 51460674 | 0.992 |
| 4 | Naive Treg cells | Plasmacytoid DCs | 4.59 | 181501650 | 0.973 |
| 4 | Neutrophils | Plasmacytoid DCs | 1.86 | 15477059 | 0.778 |
| 4 | NKT cells | Plasmacytoid DCs | 0.88 | -3377726 | 0.803 |
| 1 | Basophils | NKT cells | 1.57 | 63874173 | 0.994 |
| 1 | CD4+ T cells | NKT cells | 1.63 | 74296319 | 0.998 |
| 1 | Central memory CD4+ T cells | NKT cells | 1.81 | 86454972 | 0.998 |
| 1 | Central memory CD8+ T cells | NKT cells | 1.81 | 50289160 | 0.998 |
| 1 | Cyctotoxic NK cells | NKT cells | 2.51 | 98817376 | 0.996 |
| 1 | Cytotoxic CD4+ T cells | NKT cells | 1.35 | 46200173 | 0.977 |
| 1 | Cytotoxic CD8+ T cells | NKT cells | 1.31 | 22285064 | 0.998 |
| 1 | Effector memory CD4+ T cells | NKT cells | 1.58 | 70228019 | 0.998 |
| 1 | Effector memory CD8+ T cells | NKT cells | 0.79 | -9349632 | 0.986 |
| 1 | Exhausted CD4+ T cells | NKT cells | 2.76 | 170445608 | 0.998 |
| 1 | Exhausted CD8+ T cells | NKT cells | 2.38 | 87912093 | 0.998 |
| 1 | Immature NK cells | NKT cells | 1.15 | 39419313 | 0.998 |
| 1 | M2-like macrophages | NKT cells | 1.44 | 14636454 | 0.698 |
| 1 | Macrophages | NKT cells | 1.42 | 57808175 | 0.986 |
| 1 | Memory Treg cells | NKT cells | 2.30 | 82808763 | 0.998 |
| 1 | Myeloid cells | NKT cells | 1.49 | 56633984 | 0.994 |
| 1 | Naive B cells | NKT cells | 1.07 | 36509656 | 0.998 |
| 1 | Naive Treg cells | NKT cells | 2.33 | 104992787 | 0.998 |
| 2 | CD4+ T cells | NKT cells | 3.76 | 132304738 | 0.906 |
| 2 | CD8+ T cells | NKT cells | 1.46 | 23073437 | 0.992 |
| 2 | Central memory CD4+ T cells | NKT cells | 2.34 | 55316417 | 0.450 |
| 2 | Central memory CD8+ T cells | NKT cells | 1.01 | -2963311 | 0.205 |
| 2 | Cyctotoxic NK cells | NKT cells | 3.68 | 123872665 | 0.797 |
| 2 | Cytotoxic CD4+ T cells | NKT cells | 3.53 | 124613265 | 0.893 |
| 2 | Cytotoxic CD8+ T cells | NKT cells | 1.28 | 15064823 | 0.869 |
| 2 | Dendritic cells | NKT cells | 3.15 | 52780019 | 0.098 |
| 2 | Effector CD4+ T cells | NKT cells | 4.45 | 397259650 | 0.758 |
| 2 | Effector CD8+ T cells | NKT cells | 2.55 | 163511799 | 0.166 |
| 2 | Effector memory CD4+ T cells | NKT cells | 3.99 | 144404077 | 0.881 |
| 2 | Effector memory CD8+ T cells | NKT cells | 1.54 | 27569720 | 0.984 |
| 2 | Exhausted CD4+ T cells | NKT cells | 3.63 | 140547823 | 0.764 |
| 2 | Exhausted CD8+ T cells | NKT cells | 2.51 | 84224805 | 0.994 |
| 2 | Immature NK cells | NKT cells | 1.60 | 50529211 | 0.166 |
| 2 | M2-like macrophages | NKT cells | 3.29 | 89770431 | 0.550 |
| 2 | Macrophages | NKT cells | 2.47 | 77192480 | 0.830 |
| 2 | Mast Cells | NKT cells | 3.24 | 83439764 | 0.751 |
| 2 | Memory Treg cells | NKT cells | 2.06 | 49901509 | 0.729 |
| 2 | Myeloid cells | NKT cells | 2.24 | 65399146 | 0.908 |
| 2 | Naive B cells | NKT cells | 2.29 | 91206832 | 0.595 |
| 2 | Naive Treg cells | NKT cells | 1.90 | 47590167 | 0.805 |
| 3 | Basophils | NKT cells | 1.77 | 38130344 | 0.992 |
| 3 | CD4+ T cells | NKT cells | 2.07 | 55516525 | 0.998 |
| 3 | CD8+ T cells | NKT cells | 2.01 | 50070484 | 0.998 |
| 3 | Central memory CD4+ T cells | NKT cells | 2.03 | 52572306 | 0.998 |
| 3 | Central memory CD8+ T cells | NKT cells | 2.21 | 59564255 | 0.998 |
| 3 | Cyctotoxic NK cells | NKT cells | 2.41 | 64138939 | 0.996 |
| 3 | Cytotoxic CD4+ T cells | NKT cells | 2.10 | 57118095 | 0.998 |
| 3 | Cytotoxic CD8+ T cells | NKT cells | 2.06 | 52199695 | 0.998 |
| 3 | Effector CD4+ T cells | NKT cells | 2.03 | 52572306 | 0.998 |
| 3 | Effector CD8+ T cells | NKT cells | 0.74 | -9930576 | 0.961 |
| 3 | Effector memory CD4+ T cells | NKT cells | 2.00 | 53492239 | 0.998 |
| 3 | Effector memory CD8+ T cells | NKT cells | 0.92 | -5992675 | 0.932 |
| 3 | Exhausted CD4+ T cells | NKT cells | 1.78 | 39046538 | 0.998 |
| 3 | Exhausted CD8+ T cells | NKT cells | 2.07 | 51379895 | 0.998 |
| 3 | gdT cells | NKT cells | 3.50 | 174221108 | 0.998 |
| 3 | Immature NK cells | NKT cells | 2.15 | 56198703 | 0.994 |
| 3 | M2-like macrophages | NKT cells | 1.27 | 14287645 | 0.854 |
| 3 | Macrophages | NKT cells | 1.27 | 14287645 | 0.893 |
| 3 | Memory Treg cells | NKT cells | 2.34 | 92823374 | 0.998 |
| 3 | Myeloid cells | NKT cells | 1.67 | 34098540 | 0.975 |
| 3 | Naive B cells | NKT cells | 1.01 | -2541720 | 0.942 |
| 3 | Naive CD4+ T cells | NKT cells | 2.13 | 58117392 | 0.998 |
| 3 | Naive CD8+ T cells | NKT cells | 1.98 | 48070169 | 0.998 |
| 3 | Neutrophils | NKT cells | 2.11 | 51850625 | 0.986 |
| 4 | Basophils | NKT cells | 0.68 | -15232960 | 0.986 |
| 4 | CD4+ T cells | NKT cells | 1.43 | 26941964 | 0.998 |
| 4 | CD8+ T cells | NKT cells | 1.93 | 58304053 | 0.998 |
| 4 | Central memory CD4+ T cells | NKT cells | 1.34 | 23302602 | 0.998 |
| 4 | Central memory CD8+ T cells | NKT cells | 2.11 | 72756612 | 0.998 |
| 4 | Cyctotoxic NK cells | NKT cells | 1.45 | 19754301 | 0.990 |
| 4 | Cyctotoxic NK cells_2 | NKT cells | 1.45 | 19754301 | 0.994 |
| 4 | Cytotoxic CD4+ T cells | NKT cells | 1.40 | 25550163 | 0.998 |
| 4 | Cytotoxic CD8+ T cells | NKT cells | 1.58 | 41917920 | 0.998 |
| 4 | Dendritic cells | NKT cells | 0.79 | -9361056 | 0.979 |
| 4 | Effector CD8+ T cells | NKT cells | 2.39 | 95346111 | 0.998 |
| 4 | Effector memory CD4+ T cells | NKT cells | 1.66 | 30331885 | 0.998 |
| 4 | Effector memory CD8+ T cells | NKT cells | 1.70 | 38819115 | 0.998 |
| 4 | Exhausted CD4+ T cells | NKT cells | 1.55 | 32591820 | 0.998 |
| 4 | Exhausted CD8+ T cells | NKT cells | 1.96 | 59638222 | 0.998 |
| 4 | Immature NK cells | NKT cells | 1.40 | 28299251 | 0.994 |
| 4 | M1-like macrophages | NKT cells | 0.79 | 914861 | 0.953 |
| 4 | M2-like macrophages | NKT cells | 0.59 | -12331108 | 0.971 |
| 4 | Macrophages | NKT cells | 0.75 | -5287862 | 0.979 |
| 4 | Mast Cells | NKT cells | 0.58 | -18830039 | 0.986 |
| 4 | Memory Treg cells | NKT cells | 1.91 | 74072872 | 0.998 |
| 4 | Myeloid cells | NKT cells | 0.72 | -11930152 | 0.992 |
| 4 | Naive B cells | NKT cells | 1.94 | 60085190 | 0.994 |
| 4 | Naive CD4+ T cells | NKT cells | 1.53 | 31698919 | 0.998 |
| 4 | Naive CD8+ T cells | NKT cells | 1.77 | 48091529 | 0.998 |
| 4 | Naive Treg cells | NKT cells | 1.99 | 69489386 | 0.996 |
| 4 | Neutrophils | NKT cells | 1.09 | 7735035 | 0.994 |
| 3 | Basophils | Neutrophils | 3.11 | 233119446 | 0.998 |
| 3 | CD4+ T cells | Neutrophils | 3.74 | 318231071 | 0.994 |
| 3 | CD8+ T cells | Neutrophils | 2.20 | 43865508 | 0.793 |
| 3 | Central memory CD4+ T cells | Neutrophils | 3.75 | 347429013 | 0.996 |
| 3 | Central memory CD8+ T cells | Neutrophils | 1.85 | 10092110 | 0.957 |
| 3 | Cyctotoxic NK cells | Neutrophils | 3.27 | 136563204 | 0.998 |
| 3 | Cytotoxic CD4+ T cells | Neutrophils | 3.78 | 319998133 | 0.994 |
| 3 | Cytotoxic CD8+ T cells | Neutrophils | 2.19 | 43748054 | 0.912 |
| 3 | Effector CD4+ T cells | Neutrophils | 3.75 | 347429013 | 0.994 |
| 3 | Effector CD8+ T cells | Neutrophils | 2.05 | 60346546 | 0.953 |
| 3 | Effector memory CD4+ T cells | Neutrophils | 3.47 | 307879853 | 0.963 |
| 3 | Effector memory CD8+ T cells | Neutrophils | 1.83 | 45899827 | 0.912 |
| 3 | Exhausted CD4+ T cells | Neutrophils | 3.27 | 213802140 | 0.992 |
| 3 | Exhausted CD8+ T cells | Neutrophils | 2.42 | 54313241 | 0.760 |
| 3 | gdT cells | Neutrophils | 0.18 | -42441393 | 0.951 |
| 3 | Immature NK cells | Neutrophils | 2.58 | 69579587 | 0.971 |
| 3 | M2-like macrophages | Neutrophils | 2.29 | 83269097 | 0.998 |
| 3 | Macrophages | Neutrophils | 2.29 | 83269097 | 0.998 |
| 3 | Memory Treg cells | Neutrophils | 2.10 | 49654757 | 0.869 |
| 3 | Myeloid cells | Neutrophils | 2.02 | 112582209 | 0.998 |
| 3 | Naive B cells | Neutrophils | 0.64 | -19706567 | 0.848 |
| 3 | Naive CD4+ T cells | Neutrophils | 3.86 | 320070904 | 0.992 |
| 3 | Naive CD8+ T cells | Neutrophils | 2.98 | 109458514 | 0.983 |
| 4 | Basophils | Neutrophils | 2.39 | 97841972 | 0.998 |
| 4 | CD4+ T cells | Neutrophils | 1.51 | 13719695 | 0.934 |
| 4 | CD8+ T cells | Neutrophils | 0.98 | 2679032 | 0.803 |
| 4 | Central memory CD4+ T cells | Neutrophils | 1.66 | 35846866 | 0.994 |
| 4 | Central memory CD8+ T cells | Neutrophils | 1.02 | 8794622 | 0.977 |
| 4 | Cyctotoxic NK cells | Neutrophils | 1.60 | 23566465 | 0.967 |
| 4 | Cyctotoxic NK cells_2 | Neutrophils | 1.60 | 23566465 | 0.955 |
| 4 | Cytotoxic CD4+ T cells | Neutrophils | 2.65 | 53517894 | 0.932 |
| 4 | Cytotoxic CD8+ T cells | Neutrophils | 1.52 | 22533327 | 0.992 |
| 4 | Dendritic cells | Neutrophils | 2.50 | 74914018 | 0.998 |
| 4 | Effector CD8+ T cells | Neutrophils | 0.85 | -13975856 | 0.380 |
| 4 | Effector memory CD4+ T cells | Neutrophils | 2.20 | 1945890 | 0.688 |
| 4 | Effector memory CD8+ T cells | Neutrophils | 0.86 | -4558379 | 0.856 |
| 4 | Exhausted CD4+ T cells | Neutrophils | 1.84 | 29353088 | 0.912 |
| 4 | Exhausted CD8+ T cells | Neutrophils | 1.00 | 4261804 | 0.875 |
| 4 | Immature NK cells | Neutrophils | 2.24 | 68139854 | 0.990 |
| 4 | M1-like macrophages | Neutrophils | 0.55 | -14473912 | 0.712 |
| 4 | M2-like macrophages | Neutrophils | 2.08 | 108821719 | 0.998 |
| 4 | Macrophages | Neutrophils | 1.69 | 82212902 | 0.998 |
| 4 | Mast Cells | Neutrophils | 1.51 | 16960466 | 0.998 |
| 4 | Memory Treg cells | Neutrophils | 0.95 | 5020639 | 0.575 |
| 4 | Myeloid cells | Neutrophils | 2.21 | 81392797 | 0.998 |
| 4 | Naive B cells | Neutrophils | 1.38 | 5650652 | 0.942 |
| 4 | Naive CD4+ T cells | Neutrophils | 1.18 | -13789737 | 0.949 |
| 4 | Naive CD8+ T cells | Neutrophils | 1.30 | -7277801 | 0.936 |
| 4 | Naive Treg cells | Neutrophils | 0.69 | -13063234 | 0.059 |
| 1 | Basophils | Naive Treg cells | 0.68 | 1060712 | 0.704 |
| 1 | CD4+ T cells | Naive Treg cells | 0.03 | -47833343 | 0.899 |
| 1 | Central memory CD4+ T cells | Naive Treg cells | 0.11 | -40887436 | 0.836 |
| 1 | Central memory CD8+ T cells | Naive Treg cells | 3.84 | 197925996 | 0.998 |
| 1 | Cyctotoxic NK cells | Naive Treg cells | 3.83 | 187397096 | 0.981 |
| 1 | Cytotoxic CD4+ T cells | Naive Treg cells | 0.00 | -50421325 | 0.242 |
| 1 | Cytotoxic CD8+ T cells | Naive Treg cells | 2.37 | 98557561 | 0.984 |
| 1 | Effector memory CD4+ T cells | Naive Treg cells | 0.00 | -50421325 | 0.930 |
| 1 | Effector memory CD8+ T cells | Naive Treg cells | 1.10 | -831150 | 0.577 |
| 1 | Exhausted CD4+ T cells | Naive Treg cells | 4.58 | 363963870 | 0.998 |
| 1 | Exhausted CD8+ T cells | Naive Treg cells | 4.96 | 294461828 | 0.998 |
| 1 | Immature NK cells | Naive Treg cells | 0.16 | -44128810 | 0.955 |
| 1 | M2-like macrophages | Naive Treg cells | 0.65 | -33489717 | 0.277 |
| 1 | Macrophages | Naive Treg cells | 0.19 | -39105725 | 0.825 |
| 1 | Memory Treg cells | Naive Treg cells | 4.71 | 329758197 | 0.994 |
| 1 | Myeloid cells | Naive Treg cells | 0.62 | -8145465 | 0.700 |
| 1 | Naive B cells | Naive Treg cells | 0.00 | -50421325 | 0.951 |
| 2 | CD4+ T cells | Naive Treg cells | 1.69 | 9107443 | 0.774 |
| 2 | CD8+ T cells | Naive Treg cells | 2.21 | 72071579 | 0.998 |
| 2 | Central memory CD4+ T cells | Naive Treg cells | 3.81 | 106893213 | 0.951 |
| 2 | Central memory CD8+ T cells | Naive Treg cells | 2.16 | 52460686 | 0.955 |
| 2 | Cyctotoxic NK cells | Naive Treg cells | 2.27 | 63839901 | 0.979 |
| 2 | Cytotoxic CD4+ T cells | Naive Treg cells | 1.82 | 17602208 | 0.951 |
| 2 | Cytotoxic CD8+ T cells | Naive Treg cells | 2.11 | 63536319 | 0.998 |
| 2 | Dendritic cells | Naive Treg cells | 4.29 | 126912791 | 0.864 |
| 2 | Effector CD4+ T cells | Naive Treg cells | 0.23 | -46462715 | 0.583 |
| 2 | Effector CD8+ T cells | Naive Treg cells | 1.97 | 49358112 | 0.544 |
| 2 | Effector memory CD4+ T cells | Naive Treg cells | 1.34 | -6903149 | 0.953 |
| 2 | Effector memory CD8+ T cells | Naive Treg cells | 2.22 | 75552521 | 0.998 |
| 2 | Exhausted CD4+ T cells | Naive Treg cells | 2.32 | 46322819 | 0.963 |
| 2 | Exhausted CD8+ T cells | Naive Treg cells | 4.00 | 181843208 | 0.998 |
| 2 | Immature NK cells | Naive Treg cells | 0.42 | -38536150 | 0.060 |
| 2 | M2-like macrophages | Naive Treg cells | 1.99 | 37250637 | 0.949 |
| 2 | Macrophages | Naive Treg cells | 2.50 | 91227988 | 0.990 |
| 2 | Mast Cells | Naive Treg cells | 4.73 | 230229082 | 0.988 |
| 2 | Memory Treg cells | Naive Treg cells | 4.46 | 245210958 | 0.994 |
| 2 | Myeloid cells | Naive Treg cells | 2.33 | 85938185 | 0.990 |
| 2 | Naive B cells | Naive Treg cells | 1.42 | 30753160 | 0.735 |
| 4 | Basophils | Naive Treg cells | 2.10 | 71830250 | 0.986 |
| 4 | CD4+ T cells | Naive Treg cells | 1.69 | 51042955 | 0.994 |
| 4 | CD8+ T cells | Naive Treg cells | 1.51 | 29870600 | 0.984 |
| 4 | Central memory CD4+ T cells | Naive Treg cells | 2.08 | 105958628 | 0.998 |
| 4 | Central memory CD8+ T cells | Naive Treg cells | 2.06 | 69555965 | 0.998 |
| 4 | Cyctotoxic NK cells | Naive Treg cells | 1.40 | -5640162 | 0.437 |
| 4 | Cyctotoxic NK cells_2 | Naive Treg cells | 1.40 | -5640162 | 0.483 |
| 4 | Cytotoxic CD4+ T cells | Naive Treg cells | 1.04 | -1278445 | 0.002 |
| 4 | Cytotoxic CD8+ T cells | Naive Treg cells | 1.03 | 11624510 | 0.482 |
| 4 | Dendritic cells | Naive Treg cells | 2.66 | 90576783 | 0.981 |
| 4 | Effector CD8+ T cells | Naive Treg cells | 0.00 | -50518096 | 0.000 |
| 4 | Effector memory CD4+ T cells | Naive Treg cells | 0.06 | -49530288 | 0.423 |
| 4 | Effector memory CD8+ T cells | Naive Treg cells | 0.71 | -23584805 | 0.805 |
| 4 | Exhausted CD4+ T cells | Naive Treg cells | 1.68 | 61076758 | 0.988 |
| 4 | Exhausted CD8+ T cells | Naive Treg cells | 1.47 | 25399948 | 0.823 |
| 4 | Immature NK cells | Naive Treg cells | 1.25 | 29756773 | 0.743 |
| 4 | M1-like macrophages | Naive Treg cells | 2.23 | 193961347 | 0.801 |
| 4 | M2-like macrophages | Naive Treg cells | 2.22 | 154684627 | 0.990 |
| 4 | Macrophages | Naive Treg cells | 1.62 | 92654533 | 0.986 |
| 4 | Mast Cells | Naive Treg cells | 3.51 | 165712678 | 0.986 |
| 4 | Memory Treg cells | Naive Treg cells | 3.27 | 284907859 | 0.971 |
| 4 | Myeloid cells | Naive Treg cells | 2.29 | 84673968 | 0.988 |
| 4 | Naive B cells | Naive Treg cells | 1.75 | 49195002 | 0.530 |
| 4 | Naive CD4+ T cells | Naive Treg cells | 1.48 | -4334912 | 0.474 |
| 4 | Naive CD8+ T cells | Naive Treg cells | 1.56 | 2987155 | 0.425 |
| 3 | Basophils | Naive CD8+ T cells | 2.33 | 83129634 | 0.992 |
| 3 | CD4+ T cells | Naive CD8+ T cells | 2.77 | 111221000 | 0.998 |
| 3 | CD8+ T cells | Naive CD8+ T cells | 2.12 | 55738312 | 0.998 |
| 3 | Central memory CD4+ T cells | Naive CD8+ T cells | 2.81 | 107610075 | 0.992 |
| 3 | Central memory CD8+ T cells | Naive CD8+ T cells | 2.08 | 39686881 | 0.994 |
| 3 | Cyctotoxic NK cells | Naive CD8+ T cells | 2.57 | 78285431 | 0.992 |
| 3 | Cytotoxic CD4+ T cells | Naive CD8+ T cells | 2.79 | 113349096 | 0.998 |
| 3 | Cytotoxic CD8+ T cells | Naive CD8+ T cells | 2.16 | 56217883 | 0.998 |
| 3 | Effector CD4+ T cells | Naive CD8+ T cells | 2.81 | 107610075 | 0.992 |
| 3 | Effector CD8+ T cells | Naive CD8+ T cells | 1.33 | 30144342 | 0.994 |
| 3 | Effector memory CD4+ T cells | Naive CD8+ T cells | 2.58 | 92932667 | 0.955 |
| 3 | Effector memory CD8+ T cells | Naive CD8+ T cells | 1.12 | 1319399 | 0.507 |
| 3 | Exhausted CD4+ T cells | Naive CD8+ T cells | 2.48 | 97338251 | 0.998 |
| 3 | Exhausted CD8+ T cells | Naive CD8+ T cells | 2.30 | 64871912 | 0.998 |
| 3 | gdT cells | Naive CD8+ T cells | 1.29 | -1288956 | 0.942 |
| 3 | Immature NK cells | Naive CD8+ T cells | 1.87 | 48444427 | 0.957 |
| 3 | M2-like macrophages | Naive CD8+ T cells | 1.59 | 38870540 | 0.981 |
| 3 | Macrophages | Naive CD8+ T cells | 1.59 | 38870540 | 0.981 |
| 3 | Memory Treg cells | Naive CD8+ T cells | 1.76 | 43661184 | 0.998 |
| 3 | Myeloid cells | Naive CD8+ T cells | 2.66 | 114220215 | 0.986 |
| 3 | Naive B cells | Naive CD8+ T cells | 2.23 | 90680140 | 0.990 |
| 3 | Naive CD4+ T cells | Naive CD8+ T cells | 2.83 | 118411766 | 0.998 |
| 4 | Basophils | Naive CD8+ T cells | 0.78 | -22426073 | 0.992 |
| 4 | CD4+ T cells | Naive CD8+ T cells | 2.07 | 91209625 | 0.998 |
| 4 | CD8+ T cells | Naive CD8+ T cells | 1.06 | 2271269 | 0.895 |
| 4 | Central memory CD4+ T cells | Naive CD8+ T cells | 1.19 | 21998489 | 0.992 |
| 4 | Central memory CD8+ T cells | Naive CD8+ T cells | 1.11 | 818346 | 0.846 |
| 4 | Cyctotoxic NK cells | Naive CD8+ T cells | 2.81 | 116746962 | 0.992 |
| 4 | Cyctotoxic NK cells_2 | Naive CD8+ T cells | 2.81 | 116746962 | 0.992 |
| 4 | Cytotoxic CD4+ T cells | Naive CD8+ T cells | 2.32 | 105821147 | 0.998 |
| 4 | Cytotoxic CD8+ T cells | Naive CD8+ T cells | 1.60 | 41955694 | 0.998 |
| 4 | Dendritic cells | Naive CD8+ T cells | 1.70 | 35139274 | 0.992 |
| 4 | Effector CD8+ T cells | Naive CD8+ T cells | 3.04 | 145155324 | 0.992 |
| 4 | Effector memory CD4+ T cells | Naive CD8+ T cells | 0.08 | -47483203 | 0.971 |
| 4 | Effector memory CD8+ T cells | Naive CD8+ T cells | 0.60 | -25598038 | 0.996 |
| 4 | Exhausted CD4+ T cells | Naive CD8+ T cells | 1.66 | 57763991 | 0.998 |
| 4 | Exhausted CD8+ T cells | Naive CD8+ T cells | 0.98 | -3440437 | 0.979 |
| 4 | Immature NK cells | Naive CD8+ T cells | 2.18 | 72184481 | 0.992 |
| 4 | M1-like macrophages | Naive CD8+ T cells | 0.23 | -43669379 | 0.942 |
| 4 | M2-like macrophages | Naive CD8+ T cells | 0.52 | -31450317 | 0.984 |
| 4 | Macrophages | Naive CD8+ T cells | 0.60 | -29329267 | 0.994 |
| 4 | Mast Cells | Naive CD8+ T cells | 0.47 | -32654559 | 0.994 |
| 4 | Memory Treg cells | Naive CD8+ T cells | 1.39 | 18870394 | 0.998 |
| 4 | Myeloid cells | Naive CD8+ T cells | 0.80 | -19992999 | 0.996 |
| 4 | Naive B cells | Naive CD8+ T cells | 3.59 | 185923689 | 0.996 |
| 4 | Naive CD4+ T cells | Naive CD8+ T cells | 3.60 | 208154185 | 0.998 |
| 3 | Basophils | Naive CD4+ T cells | 3.25 | 242021540 | 0.992 |
| 3 | CD4+ T cells | Naive CD4+ T cells | 4.36 | 355312374 | 0.998 |
| 3 | CD8+ T cells | Naive CD4+ T cells | 2.35 | 53178332 | 0.998 |
| 3 | Central memory CD4+ T cells | Naive CD4+ T cells | 4.41 | 376075741 | 0.994 |
| 3 | Central memory CD8+ T cells | Naive CD4+ T cells | 2.05 | 17008383 | 0.942 |
| 3 | Cyctotoxic NK cells | Naive CD4+ T cells | 3.61 | 137015106 | 0.992 |
| 3 | Cytotoxic CD4+ T cells | Naive CD4+ T cells | 4.42 | 358943131 | 0.998 |
| 3 | Cytotoxic CD8+ T cells | Naive CD4+ T cells | 2.40 | 54774353 | 0.998 |
| 3 | Effector CD4+ T cells | Naive CD4+ T cells | 4.41 | 376075741 | 0.996 |
| 3 | Effector CD8+ T cells | Naive CD4+ T cells | 2.03 | 72071530 | 0.949 |
| 3 | Effector memory CD4+ T cells | Naive CD4+ T cells | 3.98 | 334407792 | 0.990 |
| 3 | Effector memory CD8+ T cells | Naive CD4+ T cells | 1.94 | 51378937 | 0.887 |
| 3 | Exhausted CD4+ T cells | Naive CD4+ T cells | 3.59 | 243564833 | 0.998 |
| 3 | Exhausted CD8+ T cells | Naive CD4+ T cells | 2.68 | 66089035 | 0.998 |
| 3 | gdT cells | Naive CD4+ T cells | 0.15 | -43291523 | 0.981 |
| 3 | Immature NK cells | Naive CD4+ T cells | 2.25 | 62244069 | 0.975 |
| 3 | M2-like macrophages | Naive CD4+ T cells | 1.72 | 71607290 | 0.990 |
| 3 | Macrophages | Naive CD4+ T cells | 1.72 | 71607290 | 0.990 |
| 3 | Memory Treg cells | Naive CD4+ T cells | 1.93 | 69697039 | 0.998 |
| 3 | Myeloid cells | Naive CD4+ T cells | 2.65 | 138750091 | 0.992 |
| 3 | Naive B cells | Naive CD4+ T cells | 1.20 | -13058758 | 0.963 |
| 4 | Basophils | Naive CD4+ T cells | 0.82 | -19758892 | 0.963 |
| 4 | CD4+ T cells | Naive CD4+ T cells | 2.16 | 105238100 | 0.998 |
| 4 | CD8+ T cells | Naive CD4+ T cells | 0.80 | -10789603 | 0.988 |
| 4 | Central memory CD4+ T cells | Naive CD4+ T cells | 1.15 | 25266676 | 0.992 |
| 4 | Central memory CD8+ T cells | Naive CD4+ T cells | 0.83 | -14460527 | 0.994 |
| 4 | Cyctotoxic NK cells | Naive CD4+ T cells | 2.98 | 133948689 | 0.994 |
| 4 | Cyctotoxic NK cells_2 | Naive CD4+ T cells | 2.98 | 133948689 | 0.994 |
| 4 | Cytotoxic CD4+ T cells | Naive CD4+ T cells | 2.12 | 101277647 | 0.998 |
| 4 | Cytotoxic CD8+ T cells | Naive CD4+ T cells | 1.34 | 26247174 | 0.998 |
| 4 | Dendritic cells | Naive CD4+ T cells | 2.09 | 56053806 | 0.992 |
| 4 | Effector CD8+ T cells | Naive CD4+ T cells | 2.14 | 75603482 | 0.754 |
| 4 | Effector memory CD4+ T cells | Naive CD4+ T cells | 0.03 | -48998529 | 0.975 |
| 4 | Effector memory CD8+ T cells | Naive CD4+ T cells | 0.34 | -39039721 | 0.996 |
| 4 | Exhausted CD4+ T cells | Naive CD4+ T cells | 1.54 | 55130709 | 0.998 |
| 4 | Exhausted CD8+ T cells | Naive CD4+ T cells | 0.70 | -19111114 | 0.992 |
| 4 | Immature NK cells | Naive CD4+ T cells | 2.12 | 68319858 | 0.990 |
| 4 | M1-like macrophages | Naive CD4+ T cells | 0.17 | -46123939 | 0.945 |
| 4 | M2-like macrophages | Naive CD4+ T cells | 0.54 | -29812835 | 0.973 |
| 4 | Macrophages | Naive CD4+ T cells | 0.56 | -30896454 | 0.994 |
| 4 | Mast Cells | Naive CD4+ T cells | 0.59 | -28013428 | 0.949 |
| 4 | Memory Treg cells | Naive CD4+ T cells | 1.14 | -1720511 | 0.916 |
| 4 | Myeloid cells | Naive CD4+ T cells | 0.86 | -16823611 | 0.914 |
| 4 | Naive B cells | Naive CD4+ T cells | 3.58 | 192961791 | 0.994 |
| 1 | Basophils | Naive B cells | 0.01 | -50334320 | 0.977 |
| 1 | CD4+ T cells | Naive B cells | 0.00 | -50421325 | 0.984 |
| 1 | Central memory CD4+ T cells | Naive B cells | 0.00 | -50421325 | 0.983 |
| 1 | Central memory CD8+ T cells | Naive B cells | 0.00 | -50356159 | 0.996 |
| 1 | Cyctotoxic NK cells | Naive B cells | 0.00 | -50401061 | 0.994 |
| 1 | Cytotoxic CD4+ T cells | Naive B cells | 0.00 | -50421325 | 0.799 |
| 1 | Cytotoxic CD8+ T cells | Naive B cells | 0.03 | -49901723 | 0.992 |
| 1 | Effector memory CD4+ T cells | Naive B cells | 0.00 | -50421325 | 0.986 |
| 1 | Effector memory CD8+ T cells | Naive B cells | 0.11 | -45713148 | 0.994 |
| 1 | Exhausted CD4+ T cells | Naive B cells | 0.00 | -50421325 | 0.823 |
| 1 | Exhausted CD8+ T cells | Naive B cells | 0.00 | -50421325 | 0.994 |
| 1 | Immature NK cells | Naive B cells | 5.81 | 578214096 | 0.998 |
| 1 | M2-like macrophages | Naive B cells | 0.00 | -50421325 | 0.885 |
| 1 | Macrophages | Naive B cells | 0.05 | -49324641 | 0.981 |
| 1 | Memory Treg cells | Naive B cells | 0.00 | -50421325 | 0.975 |
| 1 | Myeloid cells | Naive B cells | 0.04 | -49575386 | 0.988 |
| 2 | CD4+ T cells | Naive B cells | 3.05 | 122183655 | 0.990 |
| 2 | CD8+ T cells | Naive B cells | 1.48 | 26638436 | 0.979 |
| 2 | Central memory CD4+ T cells | Naive B cells | 2.90 | 71480903 | 0.940 |
| 2 | Central memory CD8+ T cells | Naive B cells | 1.66 | 20009766 | 0.587 |
| 2 | Cyctotoxic NK cells | Naive B cells | 2.52 | 104261480 | 0.973 |
| 2 | Cytotoxic CD4+ T cells | Naive B cells | 3.01 | 115954189 | 0.981 |
| 2 | Cytotoxic CD8+ T cells | Naive B cells | 1.47 | 24725543 | 0.961 |
| 2 | Dendritic cells | Naive B cells | 3.31 | 97186180 | 0.404 |
| 2 | Effector CD4+ T cells | Naive B cells | 3.87 | 238232708 | 0.439 |
| 2 | Effector CD8+ T cells | Naive B cells | 2.28 | 111375614 | 0.577 |
| 2 | Effector memory CD4+ T cells | Naive B cells | 3.08 | 130248201 | 0.988 |
| 2 | Effector memory CD8+ T cells | Naive B cells | 1.45 | 27795955 | 0.979 |
| 2 | Exhausted CD4+ T cells | Naive B cells | 3.10 | 114266953 | 0.975 |
| 2 | Exhausted CD8+ T cells | Naive B cells | 2.41 | 74218546 | 0.977 |
| 2 | Immature NK cells | Naive B cells | 1.65 | 63814308 | 0.179 |
| 2 | M2-like macrophages | Naive B cells | 2.61 | 92295351 | 0.891 |
| 2 | Macrophages | Naive B cells | 2.06 | 71962140 | 0.994 |
| 2 | Mast Cells | Naive B cells | 2.06 | 88984706 | 0.934 |
| 2 | Memory Treg cells | Naive B cells | 1.63 | 44590349 | 0.801 |
| 2 | Myeloid cells | Naive B cells | 1.94 | 64485286 | 0.994 |
| 3 | Basophils | Naive B cells | 0.89 | 4848795 | 0.782 |
| 3 | CD4+ T cells | Naive B cells | 0.74 | -23422640 | 0.986 |
| 3 | CD8+ T cells | Naive B cells | 1.52 | 24530719 | 0.981 |
| 3 | Central memory CD4+ T cells | Naive B cells | 0.37 | -33138637 | 0.973 |
| 3 | Central memory CD8+ T cells | Naive B cells | 1.52 | 6864902 | 0.977 |
| 3 | Cyctotoxic NK cells | Naive B cells | 0.77 | -17863552 | 0.990 |
| 3 | Cytotoxic CD4+ T cells | Naive B cells | 0.75 | -23440487 | 0.988 |
| 3 | Cytotoxic CD8+ T cells | Naive B cells | 1.51 | 20320758 | 0.961 |
| 3 | Effector CD4+ T cells | Naive B cells | 0.37 | -33138637 | 0.975 |
| 3 | Effector CD8+ T cells | Naive B cells | 1.18 | 32396985 | 0.986 |
| 3 | Effector memory CD4+ T cells | Naive B cells | 0.40 | -34080370 | 0.932 |
| 3 | Effector memory CD8+ T cells | Naive B cells | 0.28 | -37883208 | 0.979 |
| 3 | Exhausted CD4+ T cells | Naive B cells | 1.12 | -12400301 | 0.940 |
| 3 | Exhausted CD8+ T cells | Naive B cells | 1.58 | 27347780 | 0.971 |
| 3 | gdT cells | Naive B cells | 0.66 | -20507256 | 0.936 |
| 3 | Immature NK cells | Naive B cells | 0.54 | -22071389 | 0.975 |
| 3 | M2-like macrophages | Naive B cells | 1.21 | 35289183 | 0.957 |
| 3 | Macrophages | Naive B cells | 1.21 | 35289183 | 0.957 |
| 3 | Memory Treg cells | Naive B cells | 1.01 | -18389159 | 0.869 |
| 3 | Myeloid cells | Naive B cells | 3.34 | 175139664 | 0.893 |
| 4 | Basophils | Naive B cells | 0.77 | -19273090 | 0.994 |
| 4 | CD4+ T cells | Naive B cells | 2.07 | 85265318 | 0.994 |
| 4 | CD8+ T cells | Naive B cells | 1.21 | 10430418 | 0.992 |
| 4 | Central memory CD4+ T cells | Naive B cells | 1.10 | 19502697 | 0.990 |
| 4 | Central memory CD8+ T cells | Naive B cells | 1.33 | 15985552 | 0.979 |
| 4 | Cyctotoxic NK cells | Naive B cells | 3.19 | 157210830 | 0.994 |
| 4 | Cyctotoxic NK cells_2 | Naive B cells | 3.19 | 157210830 | 0.996 |
| 4 | Cytotoxic CD4+ T cells | Naive B cells | 2.03 | 85361490 | 0.994 |
| 4 | Cytotoxic CD8+ T cells | Naive B cells | 1.57 | 40568531 | 0.994 |
| 4 | Dendritic cells | Naive B cells | 1.43 | 15262472 | 0.899 |
| 4 | Effector CD8+ T cells | Naive B cells | 2.62 | 64139184 | 0.731 |
| 4 | Effector memory CD4+ T cells | Naive B cells | 0.13 | -45407111 | 0.973 |
| 4 | Effector memory CD8+ T cells | Naive B cells | 0.66 | -24905621 | 0.994 |
| 4 | Exhausted CD4+ T cells | Naive B cells | 1.61 | 57870094 | 0.994 |
| 4 | Exhausted CD8+ T cells | Naive B cells | 1.13 | 5068177 | 0.992 |
| 4 | Immature NK cells | Naive B cells | 2.20 | 74652168 | 0.998 |
| 4 | M1-like macrophages | Naive B cells | 0.44 | -34081811 | 0.895 |
| 4 | M2-like macrophages | Naive B cells | 0.58 | -26679596 | 0.984 |
| 4 | Macrophages | Naive B cells | 0.68 | -21651057 | 0.992 |
| 4 | Mast Cells | Naive B cells | 0.53 | -29399649 | 0.994 |
| 4 | Memory Treg cells | Naive B cells | 1.52 | 30412674 | 0.967 |
| 4 | Myeloid cells | Naive B cells | 0.81 | -17123898 | 0.996 |
| 1 | Basophils | Myeloid cells | 4.41 | 407415002 | 0.998 |
| 1 | CD4+ T cells | Myeloid cells | 7.01 | 606705164 | 0.998 |
| 1 | Central memory CD4+ T cells | Myeloid cells | 7.96 | 630572329 | 0.994 |
| 1 | Central memory CD8+ T cells | Myeloid cells | 0.66 | -9773574 | 0.996 |
| 1 | Cyctotoxic NK cells | Myeloid cells | 1.90 | 32960763 | 0.998 |
| 1 | Cytotoxic CD4+ T cells | Myeloid cells | 5.72 | 544082942 | 0.986 |
| 1 | Cytotoxic CD8+ T cells | Myeloid cells | 0.39 | -31454729 | 0.992 |
| 1 | Effector memory CD4+ T cells | Myeloid cells | 6.70 | 599237362 | 0.998 |
| 1 | Effector memory CD8+ T cells | Myeloid cells | 0.49 | -19018662 | 0.994 |
| 1 | Exhausted CD4+ T cells | Myeloid cells | 0.69 | 19095080 | 0.708 |
| 1 | Exhausted CD8+ T cells | Myeloid cells | 0.69 | -11180058 | 0.992 |
| 1 | Immature NK cells | Myeloid cells | 0.43 | -31195880 | 0.975 |
| 1 | M2-like macrophages | Myeloid cells | 3.71 | 209311361 | 0.994 |
| 1 | Macrophages | Myeloid cells | 4.82 | 478664530 | 0.998 |
| 1 | Memory Treg cells | Myeloid cells | 0.61 | -15784785 | 0.754 |
| 2 | CD4+ T cells | Myeloid cells | 3.46 | 148741638 | 0.996 |
| 2 | CD8+ T cells | Myeloid cells | 1.75 | 43170685 | 0.996 |
| 2 | Central memory CD4+ T cells | Myeloid cells | 4.00 | 167667065 | 0.992 |
| 2 | Central memory CD8+ T cells | Myeloid cells | 2.11 | 58200222 | 0.990 |
| 2 | Cyctotoxic NK cells | Myeloid cells | 3.17 | 134984497 | 0.998 |
| 2 | Cytotoxic CD4+ T cells | Myeloid cells | 3.48 | 150585855 | 0.994 |
| 2 | Cytotoxic CD8+ T cells | Myeloid cells | 1.74 | 42409297 | 0.996 |
| 2 | Dendritic cells | Myeloid cells | 4.37 | 201097850 | 0.998 |
| 2 | Effector CD4+ T cells | Myeloid cells | 3.76 | 126607725 | 0.908 |
| 2 | Effector CD8+ T cells | Myeloid cells | 2.62 | 75984363 | 0.795 |
| 2 | Effector memory CD4+ T cells | Myeloid cells | 3.37 | 145591788 | 0.994 |
| 2 | Effector memory CD8+ T cells | Myeloid cells | 1.68 | 40619553 | 0.998 |
| 2 | Exhausted CD4+ T cells | Myeloid cells | 3.80 | 161052638 | 0.992 |
| 2 | Exhausted CD8+ T cells | Myeloid cells | 3.35 | 136049310 | 0.994 |
| 2 | Immature NK cells | Myeloid cells | 1.32 | 15491458 | 0.854 |
| 2 | M2-like macrophages | Myeloid cells | 3.11 | 125551793 | 0.998 |
| 2 | Macrophages | Myeloid cells | 2.57 | 107212120 | 0.998 |
| 2 | Mast Cells | Myeloid cells | 3.18 | 158433193 | 0.998 |
| 2 | Memory Treg cells | Myeloid cells | 2.39 | 96147325 | 0.988 |
| 3 | Basophils | Myeloid cells | 1.92 | 113957219 | 0.998 |
| 3 | CD4+ T cells | Myeloid cells | 2.15 | 118002242 | 0.994 |
| 3 | CD8+ T cells | Myeloid cells | 2.65 | 89615391 | 0.965 |
| 3 | Central memory CD4+ T cells | Myeloid cells | 1.80 | 103158073 | 0.994 |
| 3 | Central memory CD8+ T cells | Myeloid cells | 2.95 | 95295462 | 0.983 |
| 3 | Cyctotoxic NK cells | Myeloid cells | 1.78 | 39025563 | 0.998 |
| 3 | Cytotoxic CD4+ T cells | Myeloid cells | 2.19 | 119590702 | 0.994 |
| 3 | Cytotoxic CD8+ T cells | Myeloid cells | 2.73 | 90912336 | 0.969 |
| 3 | Effector CD4+ T cells | Myeloid cells | 1.80 | 103158073 | 0.994 |
| 3 | Effector CD8+ T cells | Myeloid cells | 1.30 | 6781887 | 0.579 |
| 3 | Effector memory CD4+ T cells | Myeloid cells | 1.95 | 99640045 | 0.953 |
| 3 | Effector memory CD8+ T cells | Myeloid cells | 1.12 | -6072297 | 0.908 |
| 3 | Exhausted CD4+ T cells | Myeloid cells | 2.12 | 100708191 | 0.992 |
| 3 | Exhausted CD8+ T cells | Myeloid cells | 2.81 | 98734940 | 0.965 |
| 3 | gdT cells | Myeloid cells | 0.23 | -35378045 | 0.973 |
| 3 | Immature NK cells | Myeloid cells | 1.40 | 16705778 | 0.953 |
| 3 | M2-like macrophages | Myeloid cells | 1.90 | 105117302 | 0.998 |
| 3 | Macrophages | Myeloid cells | 1.90 | 105117302 | 0.998 |
| 3 | Memory Treg cells | Myeloid cells | 1.34 | 21781965 | 0.953 |
| 4 | Basophils | Myeloid cells | 3.09 | 134191872 | 0.998 |
| 4 | CD4+ T cells | Myeloid cells | 1.60 | 34453954 | 0.994 |
| 4 | CD8+ T cells | Myeloid cells | 0.50 | -23513109 | 0.998 |
| 4 | Central memory CD4+ T cells | Myeloid cells | 2.22 | 79694667 | 0.994 |
| 4 | Central memory CD8+ T cells | Myeloid cells | 0.63 | -12908918 | 0.990 |
| 4 | Cyctotoxic NK cells | Myeloid cells | 1.54 | 13852978 | 0.992 |
| 4 | Cyctotoxic NK cells_2 | Myeloid cells | 1.54 | 13852978 | 0.992 |
| 4 | Cytotoxic CD4+ T cells | Myeloid cells | 1.67 | 26841323 | 0.988 |
| 4 | Cytotoxic CD8+ T cells | Myeloid cells | 0.89 | -8556315 | 0.988 |
| 4 | Dendritic cells | Myeloid cells | 3.00 | 113170389 | 0.998 |
| 4 | Effector CD8+ T cells | Myeloid cells | 0.30 | -46213616 | 0.961 |
| 4 | Effector memory CD4+ T cells | Myeloid cells | 1.11 | -16426212 | 0.973 |
| 4 | Effector memory CD8+ T cells | Myeloid cells | 0.26 | -39380095 | 0.998 |
| 4 | Exhausted CD4+ T cells | Myeloid cells | 1.77 | 45565271 | 0.994 |
| 4 | Exhausted CD8+ T cells | Myeloid cells | 0.45 | -25862272 | 0.998 |
| 4 | Immature NK cells | Myeloid cells | 1.57 | 37265591 | 0.998 |
| 4 | M1-like macrophages | Myeloid cells | 2.24 | 99670433 | 0.998 |
| 4 | M2-like macrophages | Myeloid cells | 3.48 | 168927311 | 0.998 |
| 4 | Macrophages | Myeloid cells | 2.53 | 109269200 | 0.998 |
| 4 | Mast Cells | Myeloid cells | 3.88 | 176212369 | 0.998 |
| 4 | Memory Treg cells | Myeloid cells | 2.40 | 98808142 | 0.994 |
| 1 | Basophils | Memory Treg cells | 0.83 | -5231656 | 0.636 |
| 1 | CD4+ T cells | Memory Treg cells | 0.04 | -48112059 | 0.973 |
| 1 | Central memory CD4+ T cells | Memory Treg cells | 0.14 | -41914202 | 0.866 |
| 1 | Central memory CD8+ T cells | Memory Treg cells | 4.12 | 235515903 | 0.998 |
| 1 | Cyctotoxic NK cells | Memory Treg cells | 3.81 | 151316993 | 0.969 |
| 1 | Cytotoxic CD4+ T cells | Memory Treg cells | 0.00 | -50421325 | 0.659 |
| 1 | Cytotoxic CD8+ T cells | Memory Treg cells | 2.67 | 119902790 | 0.998 |
| 1 | Effector memory CD4+ T cells | Memory Treg cells | 0.00 | -50421325 | 0.977 |
| 1 | Effector memory CD8+ T cells | Memory Treg cells | 1.28 | 3267320 | 0.961 |
| 1 | Exhausted CD4+ T cells | Memory Treg cells | 6.09 | 319432156 | 0.981 |
| 1 | Exhausted CD8+ T cells | Memory Treg cells | 5.04 | 347831304 | 0.998 |
| 1 | Immature NK cells | Memory Treg cells | 0.12 | -46234356 | 0.983 |
| 1 | M2-like macrophages | Memory Treg cells | 0.71 | -28809239 | 0.577 |
| 1 | Macrophages | Memory Treg cells | 0.23 | -39850121 | 0.971 |
| 2 | CD4+ T cells | Memory Treg cells | 2.15 | 30324153 | 0.998 |
| 2 | CD8+ T cells | Memory Treg cells | 2.28 | 72830163 | 0.998 |
| 2 | Central memory CD4+ T cells | Memory Treg cells | 4.57 | 135005503 | 0.893 |
| 2 | Central memory CD8+ T cells | Memory Treg cells | 2.34 | 62208017 | 0.961 |
| 2 | Cyctotoxic NK cells | Memory Treg cells | 2.71 | 85278313 | 0.986 |
| 2 | Cytotoxic CD4+ T cells | Memory Treg cells | 2.33 | 39818174 | 0.856 |
| 2 | Cytotoxic CD8+ T cells | Memory Treg cells | 2.17 | 64158604 | 0.998 |
| 2 | Dendritic cells | Memory Treg cells | 4.90 | 160492682 | 0.899 |
| 2 | Effector CD4+ T cells | Memory Treg cells | 0.70 | -41905186 | 0.419 |
| 2 | Effector CD8+ T cells | Memory Treg cells | 2.10 | 50528368 | 0.591 |
| 2 | Effector memory CD4+ T cells | Memory Treg cells | 1.76 | 13164110 | 0.832 |
| 2 | Effector memory CD8+ T cells | Memory Treg cells | 2.27 | 74761684 | 0.998 |
| 2 | Exhausted CD4+ T cells | Memory Treg cells | 2.95 | 74476795 | 0.891 |
| 2 | Exhausted CD8+ T cells | Memory Treg cells | 4.34 | 202876380 | 0.998 |
| 2 | Immature NK cells | Memory Treg cells | 0.40 | -42581553 | 0.637 |
| 2 | M2-like macrophages | Memory Treg cells | 2.32 | 58279877 | 0.977 |
| 2 | Macrophages | Memory Treg cells | 2.73 | 105320860 | 0.990 |
| 2 | Mast Cells | Memory Treg cells | 5.18 | 250215103 | 0.992 |
| 3 | Basophils | Memory Treg cells | 1.88 | 34900971 | 0.754 |
| 3 | CD4+ T cells | Memory Treg cells | 1.97 | 69683548 | 0.998 |
| 3 | CD8+ T cells | Memory Treg cells | 1.49 | 26430594 | 0.998 |
| 3 | Central memory CD4+ T cells | Memory Treg cells | 2.18 | 73039668 | 0.908 |
| 3 | Central memory CD8+ T cells | Memory Treg cells | 1.49 | 21100153 | 0.961 |
| 3 | Cyctotoxic NK cells | Memory Treg cells | 1.55 | 13313603 | 0.895 |
| 3 | Cytotoxic CD4+ T cells | Memory Treg cells | 1.95 | 69648786 | 0.998 |
| 3 | Cytotoxic CD8+ T cells | Memory Treg cells | 1.46 | 23392565 | 0.998 |
| 3 | Effector CD4+ T cells | Memory Treg cells | 2.18 | 73039668 | 0.912 |
| 3 | Effector CD8+ T cells | Memory Treg cells | 1.04 | 34750403 | 0.973 |
| 3 | Effector memory CD4+ T cells | Memory Treg cells | 2.01 | 71541712 | 0.992 |
| 3 | Effector memory CD8+ T cells | Memory Treg cells | 1.14 | 24682023 | 0.998 |
| 3 | Exhausted CD4+ T cells | Memory Treg cells | 1.86 | 89340849 | 0.998 |
| 3 | Exhausted CD8+ T cells | Memory Treg cells | 1.48 | 21947559 | 0.998 |
| 3 | gdT cells | Memory Treg cells | 2.36 | 274559213 | 0.998 |
| 3 | Immature NK cells | Memory Treg cells | 1.50 | 15717490 | 0.821 |
| 3 | M2-like macrophages | Memory Treg cells | 1.49 | 6172197 | 0.604 |
| 3 | Macrophages | Memory Treg cells | 1.49 | 6172197 | 0.575 |
| 4 | Basophils | Memory Treg cells | 1.92 | 59528635 | 0.994 |
| 4 | CD4+ T cells | Memory Treg cells | 1.61 | 51750906 | 0.998 |
| 4 | CD8+ T cells | Memory Treg cells | 1.56 | 41354320 | 0.998 |
| 4 | Central memory CD4+ T cells | Memory Treg cells | 2.12 | 101327212 | 0.998 |
| 4 | Central memory CD8+ T cells | Memory Treg cells | 1.83 | 65193859 | 0.998 |
| 4 | Cyctotoxic NK cells | Memory Treg cells | 1.39 | -6037888 | 0.938 |
| 4 | Cyctotoxic NK cells_2 | Memory Treg cells | 1.39 | -6037888 | 0.942 |
| 4 | Cytotoxic CD4+ T cells | Memory Treg cells | 1.42 | 25021108 | 0.998 |
| 4 | Cytotoxic CD8+ T cells | Memory Treg cells | 1.28 | 25305660 | 0.998 |
| 4 | Dendritic cells | Memory Treg cells | 1.98 | 52988700 | 0.990 |
| 4 | Effector CD8+ T cells | Memory Treg cells | 1.86 | 75216826 | 0.669 |
| 4 | Effector memory CD4+ T cells | Memory Treg cells | 0.96 | -11388090 | 0.694 |
| 4 | Effector memory CD8+ T cells | Memory Treg cells | 1.20 | 10339336 | 0.889 |
| 4 | Exhausted CD4+ T cells | Memory Treg cells | 1.85 | 72752082 | 0.998 |
| 4 | Exhausted CD8+ T cells | Memory Treg cells | 1.56 | 40478949 | 0.998 |
| 4 | Immature NK cells | Memory Treg cells | 1.36 | 30163183 | 0.994 |
| 4 | M1-like macrophages | Memory Treg cells | 1.87 | 147876332 | 0.994 |
| 4 | M2-like macrophages | Memory Treg cells | 1.92 | 130255648 | 0.994 |
| 4 | Macrophages | Memory Treg cells | 1.43 | 73257045 | 0.994 |
| 4 | Mast Cells | Memory Treg cells | 3.04 | 137313524 | 0.994 |
| 2 | CD4+ T cells | Mast Cells | 3.52 | 145083715 | 0.994 |
| 2 | CD8+ T cells | Mast Cells | 2.04 | 76926303 | 0.986 |
| 2 | Central memory CD4+ T cells | Mast Cells | 6.00 | 340963005 | 0.992 |
| 2 | Central memory CD8+ T cells | Mast Cells | 2.67 | 117753003 | 0.834 |
| 2 | Cyctotoxic NK cells | Mast Cells | 3.46 | 190554000 | 0.998 |
| 2 | Cytotoxic CD4+ T cells | Mast Cells | 3.70 | 162982493 | 0.990 |
| 2 | Cytotoxic CD8+ T cells | Mast Cells | 1.80 | 55342415 | 0.963 |
| 2 | Dendritic cells | Mast Cells | 7.63 | 428281348 | 0.998 |
| 2 | Effector CD4+ T cells | Mast Cells | 2.81 | 19912206 | 0.242 |
| 2 | Effector CD8+ T cells | Mast Cells | 2.43 | 26262144 | 0.162 |
| 2 | Effector memory CD4+ T cells | Mast Cells | 3.11 | 112734030 | 0.994 |
| 2 | Effector memory CD8+ T cells | Mast Cells | 1.93 | 69993832 | 0.981 |
| 2 | Exhausted CD4+ T cells | Mast Cells | 4.43 | 241886433 | 0.992 |
| 2 | Exhausted CD8+ T cells | Mast Cells | 5.03 | 285358043 | 0.963 |
| 2 | Immature NK cells | Mast Cells | 0.55 | -39386298 | 0.708 |
| 2 | M2-like macrophages | Mast Cells | 3.85 | 142804008 | 0.998 |
| 2 | Macrophages | Mast Cells | 3.09 | 167355478 | 0.998 |
| 4 | Basophils | Mast Cells | 3.69 | 166885436 | 0.998 |
| 4 | CD4+ T cells | Mast Cells | 1.71 | 53680183 | 0.994 |
| 4 | CD8+ T cells | Mast Cells | 0.36 | -29011282 | 0.998 |
| 4 | Central memory CD4+ T cells | Mast Cells | 2.71 | 126435642 | 0.994 |
| 4 | Central memory CD8+ T cells | Mast Cells | 0.54 | -16055390 | 0.988 |
| 4 | Cyctotoxic NK cells | Mast Cells | 1.85 | 27165840 | 0.986 |
| 4 | Cyctotoxic NK cells_2 | Mast Cells | 1.85 | 27165840 | 0.986 |
| 4 | Cytotoxic CD4+ T cells | Mast Cells | 1.03 | 4537005 | 0.990 |
| 4 | Cytotoxic CD8+ T cells | Mast Cells | 0.49 | -27225065 | 0.996 |
| 4 | Dendritic cells | Mast Cells | 3.65 | 147397542 | 0.998 |
| 4 | Effector CD8+ T cells | Mast Cells | 0.00 | -50191314 | 0.944 |
| 4 | Effector memory CD4+ T cells | Mast Cells | 0.55 | -31974803 | 0.988 |
| 4 | Effector memory CD8+ T cells | Mast Cells | 0.09 | -47261319 | 0.998 |
| 4 | Exhausted CD4+ T cells | Mast Cells | 1.84 | 67448285 | 0.994 |
| 4 | Exhausted CD8+ T cells | Mast Cells | 0.29 | -32676957 | 0.998 |
| 4 | Immature NK cells | Mast Cells | 1.26 | 16710151 | 0.998 |
| 4 | M1-like macrophages | Mast Cells | 3.55 | 202061629 | 0.998 |
| 4 | M2-like macrophages | Mast Cells | 4.50 | 244121665 | 0.998 |
| 4 | Macrophages | Mast Cells | 2.95 | 142966158 | 0.998 |
| 1 | Basophils | Macrophages | 5.02 | 503828105 | 0.998 |
| 1 | CD4+ T cells | Macrophages | 8.36 | 779580095 | 0.998 |
| 1 | Central memory CD4+ T cells | Macrophages | 9.65 | 813408910 | 0.992 |
| 1 | Central memory CD8+ T cells | Macrophages | 0.37 | -24106644 | 0.994 |
| 1 | Cyctotoxic NK cells | Macrophages | 1.55 | 5626429 | 0.955 |
| 1 | Cytotoxic CD4+ T cells | Macrophages | 6.71 | 705516256 | 0.981 |
| 1 | Cytotoxic CD8+ T cells | Macrophages | 0.10 | -45204883 | 0.984 |
| 1 | Effector memory CD4+ T cells | Macrophages | 7.94 | 768926759 | 0.998 |
| 1 | Effector memory CD8+ T cells | Macrophages | 0.42 | -18255132 | 0.990 |
| 1 | Exhausted CD4+ T cells | Macrophages | 0.30 | -29545594 | 0.423 |
| 1 | Exhausted CD8+ T cells | Macrophages | 0.22 | -38909731 | 0.992 |
| 1 | Immature NK cells | Macrophages | 0.45 | -30395559 | 0.984 |
| 1 | M2-like macrophages | Macrophages | 4.15 | 242561990 | 0.955 |
| 2 | CD4+ T cells | Macrophages | 3.84 | 175820210 | 0.994 |
| 2 | CD8+ T cells | Macrophages | 1.81 | 48113897 | 0.996 |
| 2 | Central memory CD4+ T cells | Macrophages | 4.41 | 195336622 | 0.992 |
| 2 | Central memory CD8+ T cells | Macrophages | 2.19 | 63663929 | 0.992 |
| 2 | Cyctotoxic NK cells | Macrophages | 3.46 | 157233925 | 0.998 |
| 2 | Cytotoxic CD4+ T cells | Macrophages | 3.85 | 178118334 | 0.994 |
| 2 | Cytotoxic CD8+ T cells | Macrophages | 1.76 | 44430489 | 0.996 |
| 2 | Dendritic cells | Macrophages | 4.89 | 229593053 | 0.998 |
| 2 | Effector CD4+ T cells | Macrophages | 4.26 | 146259902 | 0.885 |
| 2 | Effector CD8+ T cells | Macrophages | 2.86 | 86336911 | 0.581 |
| 2 | Effector memory CD4+ T cells | Macrophages | 3.74 | 172576404 | 0.994 |
| 2 | Effector memory CD8+ T cells | Macrophages | 1.75 | 45480257 | 0.996 |
| 2 | Exhausted CD4+ T cells | Macrophages | 4.23 | 189927346 | 0.992 |
| 2 | Exhausted CD8+ T cells | Macrophages | 3.65 | 154174523 | 0.994 |
| 2 | Immature NK cells | Macrophages | 1.31 | 12296376 | 0.840 |
| 2 | M2-like macrophages | Macrophages | 3.44 | 145792906 | 0.998 |
| 3 | Basophils | Macrophages | 1.98 | 145244681 | 0.998 |
| 3 | CD4+ T cells | Macrophages | 1.58 | 64884632 | 0.990 |
| 3 | CD8+ T cells | Macrophages | 1.80 | 44280588 | 0.977 |
| 3 | Central memory CD4+ T cells | Macrophages | 1.51 | 64660656 | 0.975 |
| 3 | Central memory CD8+ T cells | Macrophages | 2.02 | 51774688 | 0.971 |
| 3 | Cyctotoxic NK cells | Macrophages | 1.44 | 22750745 | 0.998 |
| 3 | Cytotoxic CD4+ T cells | Macrophages | 1.55 | 64085523 | 0.990 |
| 3 | Cytotoxic CD8+ T cells | Macrophages | 1.78 | 40097828 | 0.963 |
| 3 | Effector CD4+ T cells | Macrophages | 1.51 | 64660656 | 0.963 |
| 3 | Effector CD8+ T cells | Macrophages | 0.97 | -3316554 | 0.298 |
| 3 | Effector memory CD4+ T cells | Macrophages | 1.76 | 57492807 | 0.922 |
| 3 | Effector memory CD8+ T cells | Macrophages | 1.25 | 30520942 | 0.756 |
| 3 | Exhausted CD4+ T cells | Macrophages | 1.71 | 57995587 | 0.988 |
| 3 | Exhausted CD8+ T cells | Macrophages | 1.81 | 47337551 | 0.981 |
| 3 | gdT cells | Macrophages | 0.29 | -32388304 | 0.928 |
| 3 | Immature NK cells | Macrophages | 1.77 | 38212706 | 0.984 |
| 3 | M2-like macrophages | Macrophages | 2.58 | 242150573 | 0.998 |
| 4 | Basophils | Macrophages | 2.50 | 106125481 | 0.998 |
| 4 | CD4+ T cells | Macrophages | 1.19 | 22627932 | 0.994 |
| 4 | CD8+ T cells | Macrophages | 0.61 | -14515249 | 0.990 |
| 4 | Central memory CD4+ T cells | Macrophages | 1.69 | 67808111 | 0.994 |
| 4 | Central memory CD8+ T cells | Macrophages | 0.72 | -2760606 | 0.981 |
| 4 | Cyctotoxic NK cells | Macrophages | 1.27 | -2010799 | 0.947 |
| 4 | Cyctotoxic NK cells_2 | Macrophages | 1.27 | -2010799 | 0.951 |
| 4 | Cytotoxic CD4+ T cells | Macrophages | 1.34 | 14679378 | 0.984 |
| 4 | Cytotoxic CD8+ T cells | Macrophages | 0.95 | -3804266 | 0.981 |
| 4 | Dendritic cells | Macrophages | 2.25 | 63537212 | 0.998 |
| 4 | Effector CD8+ T cells | Macrophages | 0.38 | -43202382 | 0.891 |
| 4 | Effector memory CD4+ T cells | Macrophages | 1.12 | -12670458 | 0.893 |
| 4 | Effector memory CD8+ T cells | Macrophages | 0.47 | -29178686 | 0.996 |
| 4 | Exhausted CD4+ T cells | Macrophages | 1.40 | 38024306 | 0.994 |
| 4 | Exhausted CD8+ T cells | Macrophages | 0.59 | -16347430 | 0.988 |
| 4 | Immature NK cells | Macrophages | 1.32 | 21820193 | 0.998 |
| 4 | M1-like macrophages | Macrophages | 1.46 | 86418028 | 0.998 |
| 4 | M2-like macrophages | Macrophages | 2.41 | 140761436 | 0.998 |
| 1 | Basophils | M2-like macrophages | 6.31 | 386694479 | 0.998 |
| 1 | CD4+ T cells | M2-like macrophages | 10.07 | 465067780 | 0.782 |
| 1 | Central memory CD4+ T cells | M2-like macrophages | 11.37 | 455419866 | 0.756 |
| 1 | Central memory CD8+ T cells | M2-like macrophages | 0.75 | -20666300 | 0.955 |
| 1 | Cyctotoxic NK cells | M2-like macrophages | 3.68 | 103874141 | 0.875 |
| 1 | Cytotoxic CD4+ T cells | M2-like macrophages | 7.55 | 432933663 | 0.450 |
| 1 | Cytotoxic CD8+ T cells | M2-like macrophages | 0.32 | -39974266 | 0.920 |
| 1 | Effector memory CD4+ T cells | M2-like macrophages | 9.66 | 469128805 | 0.823 |
| 1 | Effector memory CD8+ T cells | M2-like macrophages | 0.44 | -35050278 | 0.957 |
| 1 | Exhausted CD4+ T cells | M2-like macrophages | 1.44 | -17629239 | 0.000 |
| 1 | Exhausted CD8+ T cells | M2-like macrophages | 0.87 | -14806641 | 0.680 |
| 1 | Immature NK cells | M2-like macrophages | 0.46 | -27994129 | 0.867 |
| 2 | CD4+ T cells | M2-like macrophages | 4.78 | 271555787 | 0.994 |
| 2 | CD8+ T cells | M2-like macrophages | 1.69 | 43331779 | 0.990 |
| 2 | Central memory CD4+ T cells | M2-like macrophages | 5.49 | 275973464 | 0.990 |
| 2 | Central memory CD8+ T cells | M2-like macrophages | 2.22 | 77019450 | 0.975 |
| 2 | Cyctotoxic NK cells | M2-like macrophages | 4.34 | 226214881 | 0.998 |
| 2 | Cytotoxic CD4+ T cells | M2-like macrophages | 4.89 | 272423481 | 0.990 |
| 2 | Cytotoxic CD8+ T cells | M2-like macrophages | 1.63 | 38584102 | 0.981 |
| 2 | Dendritic cells | M2-like macrophages | 6.14 | 331917328 | 0.981 |
| 2 | Effector CD4+ T cells | M2-like macrophages | 7.11 | 169857280 | 0.563 |
| 2 | Effector CD8+ T cells | M2-like macrophages | 3.42 | 83447614 | 0.548 |
| 2 | Effector memory CD4+ T cells | M2-like macrophages | 4.66 | 270713319 | 0.994 |
| 2 | Effector memory CD8+ T cells | M2-like macrophages | 1.60 | 37569465 | 0.992 |
| 2 | Exhausted CD4+ T cells | M2-like macrophages | 5.50 | 301226784 | 0.990 |
| 2 | Exhausted CD8+ T cells | M2-like macrophages | 3.89 | 183889003 | 0.988 |
| 2 | Immature NK cells | M2-like macrophages | 1.88 | 11895078 | 0.109 |
| 3 | Basophils | M2-like macrophages | 1.98 | 145244681 | 0.998 |
| 3 | CD4+ T cells | M2-like macrophages | 1.58 | 64884632 | 0.986 |
| 3 | CD8+ T cells | M2-like macrophages | 1.80 | 44280588 | 0.979 |
| 3 | Central memory CD4+ T cells | M2-like macrophages | 1.51 | 64660656 | 0.965 |
| 3 | Central memory CD8+ T cells | M2-like macrophages | 2.02 | 51774688 | 0.955 |
| 3 | Cyctotoxic NK cells | M2-like macrophages | 1.44 | 22750745 | 0.998 |
| 3 | Cytotoxic CD4+ T cells | M2-like macrophages | 1.55 | 64085523 | 0.990 |
| 3 | Cytotoxic CD8+ T cells | M2-like macrophages | 1.78 | 40097828 | 0.963 |
| 3 | Effector CD4+ T cells | M2-like macrophages | 1.51 | 64660656 | 0.965 |
| 3 | Effector CD8+ T cells | M2-like macrophages | 0.97 | -3316554 | 0.316 |
| 3 | Effector memory CD4+ T cells | M2-like macrophages | 1.76 | 57492807 | 0.924 |
| 3 | Effector memory CD8+ T cells | M2-like macrophages | 1.25 | 30520942 | 0.786 |
| 3 | Exhausted CD4+ T cells | M2-like macrophages | 1.71 | 57995587 | 0.988 |
| 3 | Exhausted CD8+ T cells | M2-like macrophages | 1.81 | 47337551 | 0.981 |
| 3 | gdT cells | M2-like macrophages | 0.29 | -32388304 | 0.899 |
| 3 | Immature NK cells | M2-like macrophages | 1.77 | 38212706 | 0.963 |
| 4 | Basophils | M2-like macrophages | 3.40 | 163474681 | 0.998 |
| 4 | CD4+ T cells | M2-like macrophages | 1.45 | 48909834 | 0.994 |
| 4 | CD8+ T cells | M2-like macrophages | 0.38 | -26537094 | 0.988 |
| 4 | Central memory CD4+ T cells | M2-like macrophages | 2.18 | 115575397 | 0.994 |
| 4 | Central memory CD8+ T cells | M2-like macrophages | 0.51 | -13920227 | 0.938 |
| 4 | Cyctotoxic NK cells | M2-like macrophages | 1.68 | 10505802 | 0.984 |
| 4 | Cyctotoxic NK cells_2 | M2-like macrophages | 1.68 | 10505802 | 0.986 |
| 4 | Cytotoxic CD4+ T cells | M2-like macrophages | 1.66 | 33529134 | 0.986 |
| 4 | Cytotoxic CD8+ T cells | M2-like macrophages | 0.81 | -9443690 | 0.969 |
| 4 | Dendritic cells | M2-like macrophages | 3.22 | 115499946 | 0.998 |
| 4 | Effector CD8+ T cells | M2-like macrophages | 0.24 | -47774871 | 0.901 |
| 4 | Effector memory CD4+ T cells | M2-like macrophages | 1.22 | -6669896 | 0.893 |
| 4 | Effector memory CD8+ T cells | M2-like macrophages | 0.19 | -44040926 | 0.996 |
| 4 | Exhausted CD4+ T cells | M2-like macrophages | 1.69 | 68714404 | 0.994 |
| 4 | Exhausted CD8+ T cells | M2-like macrophages | 0.35 | -29155283 | 0.986 |
| 4 | Immature NK cells | M2-like macrophages | 1.54 | 36408270 | 0.998 |
| 4 | M1-like macrophages | M2-like macrophages | 2.07 | 160835145 | 0.998 |
| 4 | Basophils | M1-like macrophages | 1.97 | 80337155 | 0.998 |
| 4 | CD4+ T cells | M1-like macrophages | 0.98 | 27086294 | 0.914 |
| 4 | CD8+ T cells | M1-like macrophages | 1.31 | 3146404 | 0.637 |
| 4 | Central memory CD4+ T cells | M1-like macrophages | 1.64 | 89582105 | 0.994 |
| 4 | Central memory CD8+ T cells | M1-like macrophages | 1.26 | 15908185 | 0.990 |
| 4 | Cyctotoxic NK cells | M1-like macrophages | 1.18 | -17674701 | 0.891 |
| 4 | Cyctotoxic NK cells_2 | M1-like macrophages | 1.18 | -17674701 | 0.912 |
| 4 | Cytotoxic CD4+ T cells | M1-like macrophages | 0.46 | -21909914 | 0.686 |
| 4 | Cytotoxic CD8+ T cells | M1-like macrophages | 0.58 | -24285289 | 0.932 |
| 4 | Dendritic cells | M1-like macrophages | 1.81 | 54492456 | 0.998 |
| 4 | Effector CD8+ T cells | M1-like macrophages | 0.00 | -50518096 | 0.429 |
| 4 | Effector memory CD4+ T cells | M1-like macrophages | 0.69 | -28633992 | 0.827 |
| 4 | Effector memory CD8+ T cells | M1-like macrophages | 1.55 | -8007265 | 0.945 |
| 4 | Exhausted CD4+ T cells | M1-like macrophages | 1.12 | 43551837 | 0.990 |
| 4 | Exhausted CD8+ T cells | M1-like macrophages | 1.34 | 1974497 | 0.764 |
| 4 | Immature NK cells | M1-like macrophages | 0.70 | -10129074 | 0.945 |
| 1 | Basophils | Immature NK cells | 0.38 | -33691495 | 0.938 |
| 1 | CD4+ T cells | Immature NK cells | 0.42 | -28958863 | 0.940 |
| 1 | Central memory CD4+ T cells | Immature NK cells | 0.45 | -24554482 | 0.975 |
| 1 | Central memory CD8+ T cells | Immature NK cells | 0.11 | -45657751 | 0.996 |
| 1 | Cyctotoxic NK cells | Immature NK cells | 0.27 | -36008485 | 0.996 |
| 1 | Cytotoxic CD4+ T cells | Immature NK cells | 0.26 | -39981569 | 0.827 |
| 1 | Cytotoxic CD8+ T cells | Immature NK cells | 0.16 | -42669567 | 0.992 |
| 1 | Effector memory CD4+ T cells | Immature NK cells | 0.40 | -30420451 | 0.938 |
| 1 | Effector memory CD8+ T cells | Immature NK cells | 0.20 | -40180032 | 0.996 |
| 1 | Exhausted CD4+ T cells | Immature NK cells | 0.17 | -41198504 | 0.829 |
| 1 | Exhausted CD8+ T cells | Immature NK cells | 0.14 | -44893916 | 0.994 |
| 2 | CD4+ T cells | Immature NK cells | 1.58 | 13269536 | 0.657 |
| 2 | CD8+ T cells | Immature NK cells | 0.90 | -7869099 | 0.448 |
| 2 | Central memory CD4+ T cells | Immature NK cells | 0.56 | -36114352 | 0.790 |
| 2 | Central memory CD8+ T cells | Immature NK cells | 0.42 | -38084490 | 0.905 |
| 2 | Cyctotoxic NK cells | Immature NK cells | 1.23 | -1781440 | 0.232 |
| 2 | Cytotoxic CD4+ T cells | Immature NK cells | 1.50 | 11126306 | 0.511 |
| 2 | Cytotoxic CD8+ T cells | Immature NK cells | 1.04 | 3084 | 0.328 |
| 2 | Dendritic cells | Immature NK cells | 0.00 | -50496251 | 0.000 |
| 2 | Effector CD4+ T cells | Immature NK cells | 3.20 | 201836850 | 0.018 |
| 2 | Effector CD8+ T cells | Immature NK cells | 2.35 | 56061877 | 0.000 |
| 2 | Effector memory CD4+ T cells | Immature NK cells | 1.74 | 21098274 | 0.805 |
| 2 | Effector memory CD8+ T cells | Immature NK cells | 0.98 | -2679412 | 0.285 |
| 2 | Exhausted CD4+ T cells | Immature NK cells | 1.22 | 6260358 | 0.540 |
| 2 | Exhausted CD8+ T cells | Immature NK cells | 0.52 | -28883673 | 0.897 |
| 3 | Basophils | Immature NK cells | 1.88 | 46495400 | 0.869 |
| 3 | CD4+ T cells | Immature NK cells | 1.88 | 52859347 | 0.969 |
| 3 | CD8+ T cells | Immature NK cells | 1.79 | 41518190 | 0.977 |
| 3 | Central memory CD4+ T cells | Immature NK cells | 1.92 | 52431850 | 0.955 |
| 3 | Central memory CD8+ T cells | Immature NK cells | 1.93 | 47167310 | 0.981 |
| 3 | Cyctotoxic NK cells | Immature NK cells | 2.38 | 73819449 | 0.990 |
| 3 | Cytotoxic CD4+ T cells | Immature NK cells | 1.89 | 53721322 | 0.973 |
| 3 | Cytotoxic CD8+ T cells | Immature NK cells | 1.82 | 43365411 | 0.975 |
| 3 | Effector CD4+ T cells | Immature NK cells | 1.92 | 52431850 | 0.959 |
| 3 | Effector CD8+ T cells | Immature NK cells | 0.81 | -11954480 | 0.977 |
| 3 | Effector memory CD4+ T cells | Immature NK cells | 1.80 | 57149938 | 0.967 |
| 3 | Effector memory CD8+ T cells | Immature NK cells | 1.09 | -1041507 | 0.772 |
| 3 | Exhausted CD4+ T cells | Immature NK cells | 1.77 | 41907759 | 0.864 |
| 3 | Exhausted CD8+ T cells | Immature NK cells | 1.87 | 45689747 | 0.973 |
| 3 | gdT cells | Immature NK cells | 2.72 | 52851814 | 0.848 |
| 4 | Basophils | Immature NK cells | 1.70 | 41121347 | 0.998 |
| 4 | CD4+ T cells | Immature NK cells | 1.71 | 42351010 | 0.992 |
| 4 | CD8+ T cells | Immature NK cells | 0.97 | -1285417 | 0.908 |
| 4 | Central memory CD4+ T cells | Immature NK cells | 1.54 | 32237846 | 0.990 |
| 4 | Central memory CD8+ T cells | Immature NK cells | 1.07 | 4705678 | 0.988 |
| 4 | Cyctotoxic NK cells | Immature NK cells | 1.84 | 39984271 | 0.994 |
| 4 | Cyctotoxic NK cells_2 | Immature NK cells | 1.84 | 39984271 | 0.994 |
| 4 | Cytotoxic CD4+ T cells | Immature NK cells | 2.23 | 76034492 | 0.983 |
| 4 | Cytotoxic CD8+ T cells | Immature NK cells | 1.48 | 31968182 | 0.996 |
| 4 | Dendritic cells | Immature NK cells | 2.02 | 63967423 | 0.998 |
| 4 | Effector CD8+ T cells | Immature NK cells | 2.26 | 68956640 | 0.992 |
| 4 | Effector memory CD4+ T cells | Immature NK cells | 0.63 | -29478255 | 0.990 |
| 4 | Effector memory CD8+ T cells | Immature NK cells | 0.65 | -21231421 | 0.998 |
| 4 | Exhausted CD4+ T cells | Immature NK cells | 1.70 | 41621559 | 0.988 |
| 4 | Exhausted CD8+ T cells | Immature NK cells | 0.94 | -2956819 | 0.998 |
| 3 | Basophils | gdT cells | 0.21 | -37197888 | 0.928 |
| 3 | CD4+ T cells | gdT cells | 0.14 | -42853372 | 0.981 |
| 3 | CD8+ T cells | gdT cells | 1.07 | 17782305 | 0.992 |
| 3 | Central memory CD4+ T cells | gdT cells | 0.13 | -43109651 | 0.955 |
| 3 | Central memory CD8+ T cells | gdT cells | 1.39 | 46283351 | 0.992 |
| 3 | Cyctotoxic NK cells | gdT cells | 0.16 | -45060470 | 0.992 |
| 3 | Cytotoxic CD4+ T cells | gdT cells | 0.14 | -43143148 | 0.983 |
| 3 | Cytotoxic CD8+ T cells | gdT cells | 1.03 | 14729828 | 0.957 |
| 3 | Effector CD4+ T cells | gdT cells | 0.13 | -43109651 | 0.959 |
| 3 | Effector CD8+ T cells | gdT cells | 0.11 | -44433731 | 0.899 |
| 3 | Effector memory CD4+ T cells | gdT cells | 0.17 | -40690487 | 0.938 |
| 3 | Effector memory CD8+ T cells | gdT cells | 0.31 | -26536471 | 0.725 |
| 3 | Exhausted CD4+ T cells | gdT cells | 0.18 | -40416727 | 0.961 |
| 3 | Exhausted CD8+ T cells | gdT cells | 0.78 | -5339863 | 0.945 |
| 1 | Basophils | Exhausted CD8+ T cells | 0.84 | -3546672 | 0.830 |
| 1 | CD4+ T cells | Exhausted CD8+ T cells | 0.04 | -48158698 | 0.994 |
| 1 | Central memory CD4+ T cells | Exhausted CD8+ T cells | 0.15 | -42086014 | 0.984 |
| 1 | Central memory CD8+ T cells | Exhausted CD8+ T cells | 3.99 | 208922428 | 0.998 |
| 1 | Cyctotoxic NK cells | Exhausted CD8+ T cells | 3.85 | 150202535 | 0.996 |
| 1 | Cytotoxic CD4+ T cells | Exhausted CD8+ T cells | 0.00 | -50421325 | 0.942 |
| 1 | Cytotoxic CD8+ T cells | Exhausted CD8+ T cells | 2.50 | 104239652 | 0.998 |
| 1 | Effector memory CD4+ T cells | Exhausted CD8+ T cells | 0.00 | -50421325 | 0.994 |
| 1 | Effector memory CD8+ T cells | Exhausted CD8+ T cells | 1.10 | -2463473 | 0.963 |
| 1 | Exhausted CD4+ T cells | Exhausted CD8+ T cells | 6.37 | 311450584 | 0.988 |
| 2 | CD4+ T cells | Exhausted CD8+ T cells | 4.62 | 191971317 | 0.998 |
| 2 | CD8+ T cells | Exhausted CD8+ T cells | 2.27 | 63365021 | 0.998 |
| 2 | Central memory CD4+ T cells | Exhausted CD8+ T cells | 5.92 | 259003691 | 0.998 |
| 2 | Central memory CD8+ T cells | Exhausted CD8+ T cells | 2.57 | 90372618 | 0.998 |
| 2 | Cyctotoxic NK cells | Exhausted CD8+ T cells | 4.60 | 208267648 | 0.992 |
| 2 | Cytotoxic CD4+ T cells | Exhausted CD8+ T cells | 4.75 | 195967252 | 0.998 |
| 2 | Cytotoxic CD8+ T cells | Exhausted CD8+ T cells | 2.09 | 54999353 | 0.998 |
| 2 | Dendritic cells | Exhausted CD8+ T cells | 6.54 | 336021329 | 0.975 |
| 2 | Effector CD4+ T cells | Exhausted CD8+ T cells | 2.50 | 75618744 | 0.712 |
| 2 | Effector CD8+ T cells | Exhausted CD8+ T cells | 2.30 | 64721630 | 0.669 |
| 2 | Effector memory CD4+ T cells | Exhausted CD8+ T cells | 4.41 | 180969822 | 0.998 |
| 2 | Effector memory CD8+ T cells | Exhausted CD8+ T cells | 2.22 | 58787608 | 0.998 |
| 2 | Exhausted CD4+ T cells | Exhausted CD8+ T cells | 5.57 | 222386720 | 0.998 |
| 3 | Basophils | Exhausted CD8+ T cells | 1.88 | 48041479 | 0.990 |
| 3 | CD4+ T cells | Exhausted CD8+ T cells | 2.08 | 51108515 | 0.998 |
| 3 | CD8+ T cells | Exhausted CD8+ T cells | 2.11 | 66472740 | 0.998 |
| 3 | Central memory CD4+ T cells | Exhausted CD8+ T cells | 1.97 | 45256138 | 0.955 |
| 3 | Central memory CD8+ T cells | Exhausted CD8+ T cells | 2.36 | 82626717 | 0.998 |
| 3 | Cyctotoxic NK cells | Exhausted CD8+ T cells | 2.30 | 70023925 | 0.994 |
| 3 | Cytotoxic CD4+ T cells | Exhausted CD8+ T cells | 2.10 | 52226652 | 0.998 |
| 3 | Cytotoxic CD8+ T cells | Exhausted CD8+ T cells | 2.18 | 70135990 | 0.998 |
| 3 | Effector CD4+ T cells | Exhausted CD8+ T cells | 1.97 | 45256138 | 0.945 |
| 3 | Effector CD8+ T cells | Exhausted CD8+ T cells | 0.80 | -7997293 | 0.930 |
| 3 | Effector memory CD4+ T cells | Exhausted CD8+ T cells | 2.00 | 42905971 | 0.862 |
| 3 | Effector memory CD8+ T cells | Exhausted CD8+ T cells | 1.12 | 12079698 | 0.998 |
| 3 | Exhausted CD4+ T cells | Exhausted CD8+ T cells | 1.94 | 47008251 | 0.998 |
| 4 | Basophils | Exhausted CD8+ T cells | 0.38 | -18698160 | 0.998 |
| 4 | CD4+ T cells | Exhausted CD8+ T cells | 1.19 | 9628200 | 0.998 |
| 4 | CD8+ T cells | Exhausted CD8+ T cells | 2.72 | 65761662 | 0.998 |
| 4 | Central memory CD4+ T cells | Exhausted CD8+ T cells | 1.44 | 19736528 | 0.998 |
| 4 | Central memory CD8+ T cells | Exhausted CD8+ T cells | 2.77 | 68237859 | 0.998 |
| 4 | Cyctotoxic NK cells | Exhausted CD8+ T cells | 0.93 | -3032926 | 0.998 |
| 4 | Cyctotoxic NK cells_2 | Exhausted CD8+ T cells | 0.93 | -3032926 | 0.998 |
| 4 | Cytotoxic CD4+ T cells | Exhausted CD8+ T cells | 0.75 | -4329658 | 0.983 |
| 4 | Cytotoxic CD8+ T cells | Exhausted CD8+ T cells | 1.48 | 20365169 | 0.998 |
| 4 | Dendritic cells | Exhausted CD8+ T cells | 0.30 | -21228449 | 0.957 |
| 4 | Effector CD8+ T cells | Exhausted CD8+ T cells | 1.93 | 27480578 | 0.947 |
| 4 | Effector memory CD4+ T cells | Exhausted CD8+ T cells | 2.84 | 65190415 | 0.998 |
| 4 | Effector memory CD8+ T cells | Exhausted CD8+ T cells | 2.94 | 72755737 | 0.998 |
| 4 | Exhausted CD4+ T cells | Exhausted CD8+ T cells | 1.47 | 19710645 | 0.998 |
| 1 | Basophils | Exhausted CD4+ T cells | 0.73 | 35650890 | 0.630 |
| 1 | CD4+ T cells | Exhausted CD4+ T cells | 0.03 | -45749408 | 0.829 |
| 1 | Central memory CD4+ T cells | Exhausted CD4+ T cells | 0.12 | -33210413 | 0.275 |
| 1 | Central memory CD8+ T cells | Exhausted CD4+ T cells | 2.98 | 133956514 | 0.986 |
| 1 | Cyctotoxic NK cells | Exhausted CD4+ T cells | 5.48 | 331108764 | 0.953 |
| 1 | Cytotoxic CD4+ T cells | Exhausted CD4+ T cells | 0.00 | -50421325 | 0.000 |
| 1 | Cytotoxic CD8+ T cells | Exhausted CD4+ T cells | 1.55 | 33409330 | 0.743 |
| 1 | Effector memory CD4+ T cells | Exhausted CD4+ T cells | 0.00 | -50421325 | 0.856 |
| 1 | Effector memory CD8+ T cells | Exhausted CD4+ T cells | 0.44 | -28083916 | 0.926 |
| 2 | CD4+ T cells | Exhausted CD4+ T cells | 6.28 | 341688532 | 0.998 |
| 2 | CD8+ T cells | Exhausted CD4+ T cells | 1.83 | 40792777 | 0.998 |
| 2 | Central memory CD4+ T cells | Exhausted CD4+ T cells | 6.11 | 312461263 | 0.998 |
| 2 | Central memory CD8+ T cells | Exhausted CD4+ T cells | 2.41 | 81874101 | 0.998 |
| 2 | Cyctotoxic NK cells | Exhausted CD4+ T cells | 5.50 | 289465908 | 0.992 |
| 2 | Cytotoxic CD4+ T cells | Exhausted CD4+ T cells | 6.30 | 333506504 | 0.998 |
| 2 | Cytotoxic CD8+ T cells | Exhausted CD4+ T cells | 1.69 | 35333909 | 0.998 |
| 2 | Dendritic cells | Exhausted CD4+ T cells | 6.99 | 433514480 | 0.984 |
| 2 | Effector CD4+ T cells | Exhausted CD4+ T cells | 6.80 | 346831013 | 0.762 |
| 2 | Effector CD8+ T cells | Exhausted CD4+ T cells | 2.82 | 120503797 | 0.739 |
| 2 | Effector memory CD4+ T cells | Exhausted CD4+ T cells | 6.31 | 346074474 | 0.998 |
| 2 | Effector memory CD8+ T cells | Exhausted CD4+ T cells | 1.73 | 33740765 | 0.998 |
| 3 | Basophils | Exhausted CD4+ T cells | 2.87 | 165268312 | 0.992 |
| 3 | CD4+ T cells | Exhausted CD4+ T cells | 3.50 | 239939233 | 0.998 |
| 3 | CD8+ T cells | Exhausted CD4+ T cells | 2.17 | 46932018 | 0.998 |
| 3 | Central memory CD4+ T cells | Exhausted CD4+ T cells | 3.62 | 252080210 | 0.998 |
| 3 | Central memory CD8+ T cells | Exhausted CD4+ T cells | 1.96 | 18171042 | 0.850 |
| 3 | Cyctotoxic NK cells | Exhausted CD4+ T cells | 3.09 | 109223012 | 0.990 |
| 3 | Cytotoxic CD4+ T cells | Exhausted CD4+ T cells | 3.54 | 241165947 | 0.998 |
| 3 | Cytotoxic CD8+ T cells | Exhausted CD4+ T cells | 2.19 | 46466003 | 0.998 |
| 3 | Effector CD4+ T cells | Exhausted CD4+ T cells | 3.62 | 252080210 | 0.998 |
| 3 | Effector CD8+ T cells | Exhausted CD4+ T cells | 1.84 | 70856794 | 0.998 |
| 3 | Effector memory CD4+ T cells | Exhausted CD4+ T cells | 3.42 | 234119215 | 0.998 |
| 3 | Effector memory CD8+ T cells | Exhausted CD4+ T cells | 1.86 | 45326242 | 0.817 |
| 4 | Basophils | Exhausted CD4+ T cells | 1.65 | 37673094 | 0.992 |
| 4 | CD4+ T cells | Exhausted CD4+ T cells | 1.50 | 69781142 | 0.998 |
| 4 | CD8+ T cells | Exhausted CD4+ T cells | 1.25 | 22018328 | 0.998 |
| 4 | Central memory CD4+ T cells | Exhausted CD4+ T cells | 1.51 | 81894787 | 0.998 |
| 4 | Central memory CD8+ T cells | Exhausted CD4+ T cells | 1.26 | 22201052 | 0.998 |
| 4 | Cyctotoxic NK cells | Exhausted CD4+ T cells | 1.76 | 47400317 | 0.992 |
| 4 | Cyctotoxic NK cells_2 | Exhausted CD4+ T cells | 1.76 | 47400317 | 0.992 |
| 4 | Cytotoxic CD4+ T cells | Exhausted CD4+ T cells | 1.61 | 82701193 | 0.998 |
| 4 | Cytotoxic CD8+ T cells | Exhausted CD4+ T cells | 1.31 | 30146498 | 0.998 |
| 4 | Dendritic cells | Exhausted CD4+ T cells | 1.55 | 28752846 | 0.992 |
| 4 | Effector CD8+ T cells | Exhausted CD4+ T cells | 1.95 | 58074228 | 0.772 |
| 4 | Effector memory CD4+ T cells | Exhausted CD4+ T cells | 1.10 | 60997204 | 0.998 |
| 4 | Effector memory CD8+ T cells | Exhausted CD4+ T cells | 1.18 | 16318425 | 0.998 |
| 1 | Basophils | Effector memory CD8+ T cells | 0.28 | -31996413 | 0.992 |
| 1 | CD4+ T cells | Effector memory CD8+ T cells | 0.25 | -26872327 | 0.990 |
| 1 | Central memory CD4+ T cells | Effector memory CD8+ T cells | 0.25 | -24564347 | 0.988 |
| 1 | Central memory CD8+ T cells | Effector memory CD8+ T cells | 1.36 | 15859907 | 0.996 |
| 1 | Cyctotoxic NK cells | Effector memory CD8+ T cells | 0.61 | -25153687 | 0.998 |
| 1 | Cytotoxic CD4+ T cells | Effector memory CD8+ T cells | 0.27 | -33501541 | 0.930 |
| 1 | Cytotoxic CD8+ T cells | Effector memory CD8+ T cells | 1.54 | 34298709 | 0.998 |
| 1 | Effector memory CD4+ T cells | Effector memory CD8+ T cells | 0.25 | -27623331 | 0.984 |
| 2 | CD4+ T cells | Effector memory CD8+ T cells | 1.83 | 31907772 | 0.998 |
| 2 | CD8+ T cells | Effector memory CD8+ T cells | 1.67 | 27819259 | 0.998 |
| 2 | Central memory CD4+ T cells | Effector memory CD8+ T cells | 2.04 | 21917110 | 0.992 |
| 2 | Central memory CD8+ T cells | Effector memory CD8+ T cells | 1.48 | 12469137 | 0.992 |
| 2 | Cyctotoxic NK cells | Effector memory CD8+ T cells | 1.82 | 34290782 | 0.984 |
| 2 | Cytotoxic CD4+ T cells | Effector memory CD8+ T cells | 1.84 | 30815898 | 0.998 |
| 2 | Cytotoxic CD8+ T cells | Effector memory CD8+ T cells | 1.62 | 24320711 | 0.998 |
| 2 | Dendritic cells | Effector memory CD8+ T cells | 2.03 | 28568579 | 0.752 |
| 2 | Effector CD4+ T cells | Effector memory CD8+ T cells | 1.63 | 8798383 | 0.813 |
| 2 | Effector CD8+ T cells | Effector memory CD8+ T cells | 1.95 | 24652789 | 0.772 |
| 2 | Effector memory CD4+ T cells | Effector memory CD8+ T cells | 1.80 | 33563537 | 0.998 |
| 3 | Basophils | Effector memory CD8+ T cells | 1.64 | 46927299 | 0.813 |
| 3 | CD4+ T cells | Effector memory CD8+ T cells | 1.88 | 49299438 | 0.877 |
| 3 | CD8+ T cells | Effector memory CD8+ T cells | 1.02 | 4673461 | 0.797 |
| 3 | Central memory CD4+ T cells | Effector memory CD8+ T cells | 2.06 | 66170056 | 0.866 |
| 3 | Central memory CD8+ T cells | Effector memory CD8+ T cells | 0.94 | 127780 | 0.598 |
| 3 | Cyctotoxic NK cells | Effector memory CD8+ T cells | 1.36 | 19871059 | 0.920 |
| 3 | Cytotoxic CD4+ T cells | Effector memory CD8+ T cells | 1.89 | 48159852 | 0.840 |
| 3 | Cytotoxic CD8+ T cells | Effector memory CD8+ T cells | 1.02 | 4336586 | 0.780 |
| 3 | Effector CD4+ T cells | Effector memory CD8+ T cells | 2.06 | 66170056 | 0.877 |
| 3 | Effector CD8+ T cells | Effector memory CD8+ T cells | 1.23 | 22810176 | 0.992 |
| 3 | Effector memory CD4+ T cells | Effector memory CD8+ T cells | 1.96 | 34229286 | 0.423 |
| 4 | Basophils | Effector memory CD8+ T cells | 0.18 | -42975075 | 0.998 |
| 4 | CD4+ T cells | Effector memory CD8+ T cells | 1.01 | 5842393 | 0.998 |
| 4 | CD8+ T cells | Effector memory CD8+ T cells | 2.58 | 98283883 | 0.998 |
| 4 | Central memory CD4+ T cells | Effector memory CD8+ T cells | 1.27 | 22784702 | 0.994 |
| 4 | Central memory CD8+ T cells | Effector memory CD8+ T cells | 2.43 | 82441781 | 0.996 |
| 4 | Cyctotoxic NK cells | Effector memory CD8+ T cells | 0.68 | -13639037 | 0.998 |
| 4 | Cyctotoxic NK cells_2 | Effector memory CD8+ T cells | 0.68 | -13639037 | 0.998 |
| 4 | Cytotoxic CD4+ T cells | Effector memory CD8+ T cells | 0.57 | -24008863 | 0.988 |
| 4 | Cytotoxic CD8+ T cells | Effector memory CD8+ T cells | 1.29 | 20023704 | 0.998 |
| 4 | Dendritic cells | Effector memory CD8+ T cells | 0.13 | -45165521 | 0.996 |
| 4 | Effector CD8+ T cells | Effector memory CD8+ T cells | 1.69 | 39637087 | 0.856 |
| 4 | Effector memory CD4+ T cells | Effector memory CD8+ T cells | 3.05 | 141301830 | 0.998 |
| 1 | Basophils | Effector memory CD4+ T cells | 5.68 | 514395735 | 0.998 |
| 1 | CD4+ T cells | Effector memory CD4+ T cells | 8.72 | 800500653 | 0.998 |
| 1 | Central memory CD4+ T cells | Effector memory CD4+ T cells | 9.59 | 836615343 | 0.998 |
| 1 | Central memory CD8+ T cells | Effector memory CD4+ T cells | 0.23 | -29978334 | 0.965 |
| 1 | Cyctotoxic NK cells | Effector memory CD4+ T cells | 1.27 | -10768420 | 0.975 |
| 1 | Cytotoxic CD4+ T cells | Effector memory CD4+ T cells | 7.41 | 667458242 | 0.998 |
| 1 | Cytotoxic CD8+ T cells | Effector memory CD4+ T cells | 0.00 | -50421325 | 0.990 |
| 2 | CD4+ T cells | Effector memory CD4+ T cells | 5.94 | 376483590 | 0.998 |
| 2 | CD8+ T cells | Effector memory CD4+ T cells | 1.67 | 44344110 | 0.998 |
| 2 | Central memory CD4+ T cells | Effector memory CD4+ T cells | 6.45 | 286984476 | 0.998 |
| 2 | Central memory CD8+ T cells | Effector memory CD4+ T cells | 2.49 | 69723789 | 0.977 |
| 2 | Cyctotoxic NK cells | Effector memory CD4+ T cells | 4.81 | 285996716 | 0.994 |
| 2 | Cytotoxic CD4+ T cells | Effector memory CD4+ T cells | 6.02 | 368836188 | 0.998 |
| 2 | Cytotoxic CD8+ T cells | Effector memory CD4+ T cells | 1.63 | 39837296 | 0.998 |
| 2 | Dendritic cells | Effector memory CD4+ T cells | 7.08 | 328791725 | 0.983 |
| 2 | Effector CD4+ T cells | Effector memory CD4+ T cells | 10.23 | 387010858 | 0.885 |
| 2 | Effector CD8+ T cells | Effector memory CD4+ T cells | 4.68 | 163699571 | 0.690 |
| 3 | Basophils | Effector memory CD4+ T cells | 3.16 | 218900263 | 0.949 |
| 3 | CD4+ T cells | Effector memory CD4+ T cells | 3.86 | 341108375 | 0.998 |
| 3 | CD8+ T cells | Effector memory CD4+ T cells | 2.14 | 40314464 | 0.981 |
| 3 | Central memory CD4+ T cells | Effector memory CD4+ T cells | 3.75 | 359731475 | 0.988 |
| 3 | Central memory CD8+ T cells | Effector memory CD4+ T cells | 1.85 | 11682669 | 0.825 |
| 3 | Cyctotoxic NK cells | Effector memory CD4+ T cells | 3.70 | 130541982 | 0.992 |
| 3 | Cytotoxic CD4+ T cells | Effector memory CD4+ T cells | 3.92 | 343262388 | 0.998 |
| 3 | Cytotoxic CD8+ T cells | Effector memory CD4+ T cells | 2.19 | 41473846 | 0.998 |
| 3 | Effector CD4+ T cells | Effector memory CD4+ T cells | 3.75 | 359731475 | 0.992 |
| 3 | Effector CD8+ T cells | Effector memory CD4+ T cells | 1.94 | 65382104 | 0.791 |
| 4 | Basophils | Effector memory CD4+ T cells | 0.99 | -17051697 | 0.936 |
| 4 | CD4+ T cells | Effector memory CD4+ T cells | 0.81 | 33418316 | 0.953 |
| 4 | CD8+ T cells | Effector memory CD4+ T cells | 2.06 | 73894653 | 0.998 |
| 4 | Central memory CD4+ T cells | Effector memory CD4+ T cells | 1.10 | 56334901 | 0.996 |
| 4 | Central memory CD8+ T cells | Effector memory CD4+ T cells | 1.86 | 53587744 | 0.992 |
| 4 | Cyctotoxic NK cells | Effector memory CD4+ T cells | 0.86 | 2437174 | 0.715 |
| 4 | Cyctotoxic NK cells_2 | Effector memory CD4+ T cells | 0.86 | 2437174 | 0.756 |
| 4 | Cytotoxic CD4+ T cells | Effector memory CD4+ T cells | 0.64 | 16112114 | 0.951 |
| 4 | Cytotoxic CD8+ T cells | Effector memory CD4+ T cells | 1.14 | 18624176 | 0.998 |
| 4 | Dendritic cells | Effector memory CD4+ T cells | 0.62 | -29016257 | 0.944 |
| 4 | Effector CD8+ T cells | Effector memory CD4+ T cells | 0.10 | -41348997 | 0.513 |
| 2 | CD4+ T cells | Effector CD8+ T cells | 3.18 | 95692541 | 0.745 |
| 2 | CD8+ T cells | Effector CD8+ T cells | 1.88 | 34404206 | 0.809 |
| 2 | Central memory CD4+ T cells | Effector CD8+ T cells | 1.04 | 15094946 | 0.528 |
| 2 | Central memory CD8+ T cells | Effector CD8+ T cells | 1.44 | 7805825 | 0.174 |
| 2 | Cyctotoxic NK cells | Effector CD8+ T cells | 2.79 | 79620390 | 0.497 |
| 2 | Cytotoxic CD4+ T cells | Effector CD8+ T cells | 2.79 | 86629955 | 0.636 |
| 2 | Cytotoxic CD8+ T cells | Effector CD8+ T cells | 1.78 | 34707912 | 0.708 |
| 2 | Dendritic cells | Effector CD8+ T cells | 1.56 | -25991965 | 0.000 |
| 2 | Effector CD4+ T cells | Effector CD8+ T cells | 1.71 | 207771438 | 0.220 |
| 3 | Basophils | Effector CD8+ T cells | 1.75 | 41487129 | 0.641 |
| 3 | CD4+ T cells | Effector CD8+ T cells | 2.03 | 71346266 | 0.998 |
| 3 | CD8+ T cells | Effector CD8+ T cells | 0.90 | -4349581 | 0.891 |
| 3 | Central memory CD4+ T cells | Effector CD8+ T cells | 2.20 | 81266811 | 0.998 |
| 3 | Central memory CD8+ T cells | Effector CD8+ T cells | 0.52 | -33437231 | 0.971 |
| 3 | Cyctotoxic NK cells | Effector CD8+ T cells | 1.31 | 12741093 | 0.846 |
| 3 | Cytotoxic CD4+ T cells | Effector CD8+ T cells | 2.02 | 71435968 | 0.951 |
| 3 | Cytotoxic CD8+ T cells | Effector CD8+ T cells | 0.87 | -6526982 | 0.848 |
| 3 | Effector CD4+ T cells | Effector CD8+ T cells | 2.20 | 81266811 | 0.998 |
| 4 | Basophils | Effector CD8+ T cells | 0.31 | -47000602 | 0.932 |
| 4 | CD4+ T cells | Effector CD8+ T cells | 1.52 | 33794342 | 0.842 |
| 4 | CD8+ T cells | Effector CD8+ T cells | 1.85 | 41018363 | 0.930 |
| 4 | Central memory CD4+ T cells | Effector CD8+ T cells | 1.21 | 9517002 | 0.834 |
| 4 | Central memory CD8+ T cells | Effector CD8+ T cells | 1.72 | 28727706 | 0.899 |
| 4 | Cyctotoxic NK cells | Effector CD8+ T cells | 1.13 | -5773685 | 0.427 |
| 4 | Cyctotoxic NK cells_2 | Effector CD8+ T cells | 1.13 | -5773685 | 0.386 |
| 4 | Cytotoxic CD4+ T cells | Effector CD8+ T cells | 3.18 | 140081880 | 0.809 |
| 4 | Cytotoxic CD8+ T cells | Effector CD8+ T cells | 2.53 | 115036139 | 0.998 |
| 4 | Dendritic cells | Effector CD8+ T cells | 0.10 | -46439294 | 0.901 |
| 2 | CD4+ T cells | Effector CD4+ T cells | 4.77 | 161874477 | 0.864 |
| 2 | CD8+ T cells | Effector CD4+ T cells | 0.80 | -17096255 | 0.645 |
| 2 | Central memory CD4+ T cells | Effector CD4+ T cells | 1.00 | -31012722 | 0.694 |
| 2 | Central memory CD8+ T cells | Effector CD4+ T cells | 0.16 | -47746477 | 0.774 |
| 2 | Cyctotoxic NK cells | Effector CD4+ T cells | 4.20 | 144951373 | 0.606 |
| 2 | Cytotoxic CD4+ T cells | Effector CD4+ T cells | 4.24 | 133634843 | 0.698 |
| 2 | Cytotoxic CD8+ T cells | Effector CD4+ T cells | 0.72 | -20879259 | 0.647 |
| 2 | Dendritic cells | Effector CD4+ T cells | 2.42 | -19105001 | 0.000 |
| 3 | Basophils | Effector CD4+ T cells | 3.16 | 256014324 | 0.994 |
| 3 | CD4+ T cells | Effector CD4+ T cells | 4.26 | 374250993 | 0.998 |
| 3 | CD8+ T cells | Effector CD4+ T cells | 2.02 | 39427070 | 0.981 |
| 3 | Central memory CD4+ T cells | Effector CD4+ T cells | 4.27 | 411104612 | 0.998 |
| 3 | Central memory CD8+ T cells | Effector CD4+ T cells | 1.51 | -1147637 | 0.944 |
| 3 | Cyctotoxic NK cells | Effector CD4+ T cells | 3.46 | 141161128 | 0.994 |
| 3 | Cytotoxic CD4+ T cells | Effector CD4+ T cells | 4.33 | 376165917 | 0.998 |
| 3 | Cytotoxic CD8+ T cells | Effector CD4+ T cells | 2.03 | 39962377 | 0.945 |
| 2 | CD4+ T cells | Dendritic cells | 5.85 | 281626938 | 0.984 |
| 2 | CD8+ T cells | Dendritic cells | 1.94 | 48457603 | 0.963 |
| 2 | Central memory CD4+ T cells | Dendritic cells | 5.47 | 443038220 | 0.986 |
| 2 | Central memory CD8+ T cells | Dendritic cells | 2.32 | 126108293 | 0.979 |
| 2 | Cyctotoxic NK cells | Dendritic cells | 5.93 | 281607696 | 0.998 |
| 2 | Cytotoxic CD4+ T cells | Dendritic cells | 5.72 | 290740399 | 0.984 |
| 2 | Cytotoxic CD8+ T cells | Dendritic cells | 1.69 | 38608699 | 0.975 |
| 4 | Basophils | Dendritic cells | 3.09 | 108644652 | 0.998 |
| 4 | CD4+ T cells | Dendritic cells | 2.10 | 58323965 | 0.994 |
| 4 | CD8+ T cells | Dendritic cells | 0.48 | -26470423 | 0.979 |
| 4 | Central memory CD4+ T cells | Dendritic cells | 2.36 | 73092295 | 0.994 |
| 4 | Central memory CD8+ T cells | Dendritic cells | 0.58 | -20229004 | 0.981 |
| 4 | Cyctotoxic NK cells | Dendritic cells | 1.75 | 10640375 | 0.953 |
| 4 | Cyctotoxic NK cells_2 | Dendritic cells | 1.75 | 10640376 | 0.959 |
| 4 | Cytotoxic CD4+ T cells | Dendritic cells | 1.62 | 33994357 | 0.992 |
| 4 | Cytotoxic CD8+ T cells | Dendritic cells | 0.74 | -13671610 | 0.971 |
| 1 | Basophils | Cytotoxic CD8+ T cells | 0.31 | -36447910 | 0.988 |
| 1 | CD4+ T cells | Cytotoxic CD8+ T cells | 0.01 | -49809978 | 0.992 |
| 1 | Central memory CD4+ T cells | Cytotoxic CD8+ T cells | 0.05 | -48169177 | 0.984 |
| 1 | Central memory CD8+ T cells | Cytotoxic CD8+ T cells | 2.27 | 78473777 | 0.998 |
| 1 | Cyctotoxic NK cells | Cytotoxic CD8+ T cells | 1.47 | 20800307 | 0.981 |
| 1 | Cytotoxic CD4+ T cells | Cytotoxic CD8+ T cells | 0.00 | -50421325 | 0.875 |
| 2 | CD4+ T cells | Cytotoxic CD8+ T cells | 1.85 | 46377798 | 0.998 |
| 2 | CD8+ T cells | Cytotoxic CD8+ T cells | 1.68 | 36848616 | 0.998 |
| 2 | Central memory CD4+ T cells | Cytotoxic CD8+ T cells | 2.13 | 56992714 | 0.998 |
| 2 | Central memory CD8+ T cells | Cytotoxic CD8+ T cells | 1.77 | 41313501 | 0.998 |
| 2 | Cyctotoxic NK cells | Cytotoxic CD8+ T cells | 1.81 | 44113303 | 0.986 |
| 2 | Cytotoxic CD4+ T cells | Cytotoxic CD8+ T cells | 1.87 | 46917212 | 0.998 |
| 3 | Basophils | Cytotoxic CD8+ T cells | 1.71 | 38199613 | 0.981 |
| 3 | CD4+ T cells | Cytotoxic CD8+ T cells | 1.84 | 41057593 | 0.998 |
| 3 | CD8+ T cells | Cytotoxic CD8+ T cells | 2.03 | 64582794 | 0.998 |
| 3 | Central memory CD4+ T cells | Cytotoxic CD8+ T cells | 1.71 | 34628721 | 0.936 |
| 3 | Central memory CD8+ T cells | Cytotoxic CD8+ T cells | 2.31 | 84344110 | 0.998 |
| 3 | Cyctotoxic NK cells | Cytotoxic CD8+ T cells | 2.10 | 58090011 | 0.992 |
| 3 | Cytotoxic CD4+ T cells | Cytotoxic CD8+ T cells | 1.86 | 42207736 | 0.998 |
| 4 | Basophils | Cytotoxic CD8+ T cells | 0.91 | -7426033 | 0.986 |
| 4 | CD4+ T cells | Cytotoxic CD8+ T cells | 1.18 | 19645156 | 0.998 |
| 4 | CD8+ T cells | Cytotoxic CD8+ T cells | 1.38 | 25607291 | 0.998 |
| 4 | Central memory CD4+ T cells | Cytotoxic CD8+ T cells | 1.06 | 14288089 | 0.998 |
| 4 | Central memory CD8+ T cells | Cytotoxic CD8+ T cells | 1.43 | 28692191 | 0.998 |
| 4 | Cyctotoxic NK cells | Cytotoxic CD8+ T cells | 1.23 | 10126962 | 0.986 |
| 4 | Cyctotoxic NK cells_2 | Cytotoxic CD8+ T cells | 1.23 | 10126961 | 0.990 |
| 4 | Cytotoxic CD4+ T cells | Cytotoxic CD8+ T cells | 1.60 | 55435276 | 0.998 |
| 1 | Basophils | Cytotoxic CD4+ T cells | 5.76 | 538129337 | 0.973 |
| 1 | CD4+ T cells | Cytotoxic CD4+ T cells | 10.06 | 806411302 | 0.998 |
| 1 | Central memory CD4+ T cells | Cytotoxic CD4+ T cells | 12.17 | 804043396 | 0.967 |
| 1 | Central memory CD8+ T cells | Cytotoxic CD4+ T cells | 0.29 | -32417520 | 0.906 |
| 1 | Cyctotoxic NK cells | Cytotoxic CD4+ T cells | 0.70 | -28634293 | 0.604 |
| 2 | CD4+ T cells | Cytotoxic CD4+ T cells | 6.20 | 363189889 | 0.998 |
| 2 | CD8+ T cells | Cytotoxic CD4+ T cells | 1.81 | 46895916 | 0.998 |
| 2 | Central memory CD4+ T cells | Cytotoxic CD4+ T cells | 6.16 | 309259959 | 0.998 |
| 2 | Central memory CD8+ T cells | Cytotoxic CD4+ T cells | 2.50 | 81242461 | 0.998 |
| 2 | Cyctotoxic NK cells | Cytotoxic CD4+ T cells | 5.32 | 286437476 | 0.992 |
| 3 | Basophils | Cytotoxic CD4+ T cells | 3.22 | 239293768 | 0.992 |
| 3 | CD4+ T cells | Cytotoxic CD4+ T cells | 4.28 | 354356601 | 0.998 |
| 3 | CD8+ T cells | Cytotoxic CD4+ T cells | 2.29 | 50478655 | 0.998 |
| 3 | Central memory CD4+ T cells | Cytotoxic CD4+ T cells | 4.30 | 375741358 | 0.998 |
| 3 | Central memory CD8+ T cells | Cytotoxic CD4+ T cells | 1.97 | 15556392 | 0.945 |
| 3 | Cyctotoxic NK cells | Cytotoxic CD4+ T cells | 3.61 | 135672521 | 0.992 |
| 4 | Basophils | Cytotoxic CD4+ T cells | 1.77 | 32158093 | 0.988 |
| 4 | CD4+ T cells | Cytotoxic CD4+ T cells | 1.58 | 73437296 | 0.998 |
| 4 | CD8+ T cells | Cytotoxic CD4+ T cells | 0.80 | -8485081 | 0.975 |
| 4 | Central memory CD4+ T cells | Cytotoxic CD4+ T cells | 1.27 | 58371529 | 0.998 |
| 4 | Central memory CD8+ T cells | Cytotoxic CD4+ T cells | 0.80 | -9486825 | 0.981 |
| 4 | Cyctotoxic NK cells | Cytotoxic CD4+ T cells | 1.91 | 52862049 | 0.986 |
| 4 | Cyctotoxic NK cells_2 | Cytotoxic CD4+ T cells | 1.91 | 52862049 | 0.986 |
| 4 | Basophils | Cyctotoxic NK cells_2 | 1.38 | 9279815 | 0.988 |
| 4 | CD4+ T cells | Cyctotoxic NK cells_2 | 2.10 | 68827771 | 0.992 |
| 4 | CD8+ T cells | Cyctotoxic NK cells_2 | 1.00 | -748758 | 0.903 |
| 4 | Central memory CD4+ T cells | Cyctotoxic NK cells_2 | 1.47 | 25863253 | 0.990 |
| 4 | Central memory CD8+ T cells | Cyctotoxic NK cells_2 | 1.04 | -3541949 | 0.975 |
| 4 | Cyctotoxic NK cells | Cyctotoxic NK cells_2 | 2.91 | 155053005 | 0.998 |
| 1 | Basophils | Cyctotoxic NK cells | 2.55 | 58686566 | 0.998 |
| 1 | CD4+ T cells | Cyctotoxic NK cells | 2.15 | 16770698 | 0.990 |
| 1 | Central memory CD4+ T cells | Cyctotoxic NK cells | 2.63 | 35198897 | 0.988 |
| 1 | Central memory CD8+ T cells | Cyctotoxic NK cells | 2.45 | 78121200 | 0.992 |
| 2 | CD4+ T cells | Cyctotoxic NK cells | 4.78 | 277237880 | 0.996 |
| 2 | CD8+ T cells | Cyctotoxic NK cells | 1.67 | 47432068 | 0.988 |
| 2 | Central memory CD4+ T cells | Cyctotoxic NK cells | 6.21 | 284964303 | 0.992 |
| 2 | Central memory CD8+ T cells | Cyctotoxic NK cells | 2.39 | 77738011 | 0.965 |
| 3 | Basophils | Cyctotoxic NK cells | 2.31 | 87940640 | 0.998 |
| 3 | CD4+ T cells | Cyctotoxic NK cells | 3.01 | 124268681 | 0.992 |
| 3 | CD8+ T cells | Cyctotoxic NK cells | 2.18 | 57746061 | 0.996 |
| 3 | Central memory CD4+ T cells | Cyctotoxic NK cells | 3.01 | 133653165 | 0.994 |
| 3 | Central memory CD8+ T cells | Cyctotoxic NK cells | 2.20 | 56882324 | 0.992 |
| 4 | Basophils | Cyctotoxic NK cells | 1.38 | 9279815 | 0.986 |
| 4 | CD4+ T cells | Cyctotoxic NK cells | 2.10 | 68827771 | 0.992 |
| 4 | CD8+ T cells | Cyctotoxic NK cells | 1.00 | -748758 | 0.842 |
| 4 | Central memory CD4+ T cells | Cyctotoxic NK cells | 1.47 | 25863253 | 0.988 |
| 4 | Central memory CD8+ T cells | Cyctotoxic NK cells | 1.04 | -3541949 | 0.973 |
| 1 | Basophils | Central memory CD8+ T cells | 0.68 | -9489191 | 0.994 |
| 1 | CD4+ T cells | Central memory CD8+ T cells | 0.21 | -27993890 | 0.988 |
| 1 | Central memory CD4+ T cells | Central memory CD8+ T cells | 0.27 | -23330339 | 0.986 |
| 2 | CD4+ T cells | Central memory CD8+ T cells | 2.75 | 82740293 | 0.998 |
| 2 | CD8+ T cells | Central memory CD8+ T cells | 1.72 | 35896169 | 0.998 |
| 2 | Central memory CD4+ T cells | Central memory CD8+ T cells | 2.70 | 144534547 | 0.998 |
| 3 | Basophils | Central memory CD8+ T cells | 1.42 | 17236179 | 0.883 |
| 3 | CD4+ T cells | Central memory CD8+ T cells | 1.33 | 1710073 | 0.953 |
| 3 | CD8+ T cells | Central memory CD8+ T cells | 2.09 | 73763419 | 0.998 |
| 3 | Central memory CD4+ T cells | Central memory CD8+ T cells | 1.11 | -8228780 | 0.949 |
| 4 | Basophils | Central memory CD8+ T cells | 0.51 | -20626034 | 0.984 |
| 4 | CD4+ T cells | Central memory CD8+ T cells | 1.13 | 11840002 | 0.998 |
| 4 | CD8+ T cells | Central memory CD8+ T cells | 2.34 | 88631664 | 0.998 |
| 4 | Central memory CD4+ T cells | Central memory CD8+ T cells | 1.26 | 26090899 | 0.998 |
| 1 | Basophils | Central memory CD4+ T cells | 5.84 | 467469227 | 0.996 |
| 1 | CD4+ T cells | Central memory CD4+ T cells | 7.91 | 727329296 | 0.998 |
| 2 | CD4+ T cells | Central memory CD4+ T cells | 6.37 | 291265465 | 0.998 |
| 2 | CD8+ T cells | Central memory CD4+ T cells | 2.21 | 53421996 | 0.998 |
| 3 | Basophils | Central memory CD4+ T cells | 3.16 | 256014324 | 0.992 |
| 3 | CD4+ T cells | Central memory CD4+ T cells | 4.26 | 374250993 | 0.998 |
| 3 | CD8+ T cells | Central memory CD4+ T cells | 2.02 | 39427070 | 0.983 |
| 4 | Basophils | Central memory CD4+ T cells | 2.01 | 65828048 | 0.994 |
| 4 | CD4+ T cells | Central memory CD4+ T cells | 1.44 | 68558267 | 0.998 |
| 4 | CD8+ T cells | Central memory CD4+ T cells | 1.17 | 20800212 | 0.998 |
| 2 | CD4+ T cells | CD8+ T cells | 1.98 | 30276131 | 0.998 |
| 3 | Basophils | CD8+ T cells | 1.72 | 39820504 | 0.983 |
| 3 | CD4+ T cells | CD8+ T cells | 1.82 | 39802990 | 0.998 |
| 4 | Basophils | CD8+ T cells | 0.41 | -16372634 | 0.998 |
| 4 | CD4+ T cells | CD8+ T cells | 1.25 | 11757156 | 0.998 |
| 1 | Basophils | CD4+ T cells | 5.72 | 502616547 | 0.998 |
| 3 | Basophils | CD4+ T cells | 3.20 | 238331962 | 0.992 |
| 4 | Basophils | CD4+ T cells | 1.47 | 25974256 | 0.994 |

**Table S1. Complete results of pairwise spatial analysis.**

| **Dilutio n factor**  **1:x** | **Species** | **Antibody** | **Antigen** | **Clone** | **Short name** | **Order number** | **Fluorochrome** |
| --- | --- | --- | --- | --- | --- | --- | --- |
| **50** | human | CD45RA | CD45RA | REAL164 | CD45RA_6 | 130-112-  097 | APC |
| **50** | human | CD56 | CD56 | REAL114 2 | CD56 | 130-128-  467 | FITC |
| **50** | human | CD19 | CD19 | REAL106 | CD19 | 130-118-  057 | PE |
| **50** | human | TCRgd | TCRγ/δ | 11F2 | TCRγ/δ | 130-114-  026 | APC |
| **50** | human | IL17A | IL17A | Polyclona l | IL17A | NBP1- 76337F | FITC |
| **50** | human | CD193_CCR3 | CD193_CCR3 | 5E8-G9- B4 | CD193 | NBP2- 81046PE | PE |
| **50** | human | CD123 | CD123 | REA918 | REA918 | 130-115-  265 | APC |
| **40** | human | CD3 | CD3 | REA1151 | CD3 | 130-120-  267 | FITC |
| **50** | human | CD66b | CD66b | REA306 | CD66b | 130-122-  922 | PE |
| **50** | human | CD45RO | CD45RO | REA611 | CD45RO | 130-113-  559 | PE |
| **50** | human | CD196 | CD196 | REA190 | CD196 | 130-117-  375 | APC |
| **50** | human | FcepsilonRIalph a | FcεRIα | REA758 | FceRIa | 130-110-  726 | FITC |
| **50** | human | CD16 | CD16 | REA1324 | CD16 | 130-128-  774 | PE |
| **50** | human | Siglec_8 | Siglec-8 | REA1045 | Siglec-8 | 130-117-  975 | APC |
| **50** | human | MAF | MAF | Polyclona l | MAF | NBP2- 24551F | FITC |
| **50** | human | CD194_CCR4 | CD194_CCR4 | MM0064- 9G12 | CD194_CCR4 | NBP2- 12129PE | PE |
| **50** | human | CD197_CCR7 | CD197_CCR7 | Monoclon al | CD197 | 566763 | APC |
| **50** | human | IgD | IgD | IgD26 | IgD | 130-124-  213 | FITC |
| **50** | human | CD185_CXCR5 | CD185_CXCR5 | MU5UBE E | CD185_CXCR5 | 12-9185-  42 | PE |
| **50** | human | CD8a | CD8a | REA1024 | CD8a | 130-117-  200 | FITC |
| **50** | human | CD279_PD1 | CD279_PD1 | REA1165 | CD279_PD1 | 130-120-  382 | PE |
| **50** | human | CD127 | CD127 | MB15- 18C9 | CD127 | 130-113-  407 | APC |
| **25** | human | CD24 | CD24 | SN3 | CD24 | ab30350 | FITC |
| **50** | human | CD45 | CD45 | 5B1 | CD45 | 130-113-  118 | PE |
| **50** | human | p63 | p63 | D9L7L | p63 | 39692 | FITC |
| **50** | human | IRF-5 | IRF-5 | E9I4Z | IRF-5 | 68013 | PE |
| **50** | human | Mast_Cell_Trypt ase | Mast_Cell_Trypt ase | REAL798 | Mast_Cell_Trypt ase | 130-125-  278 | APC |
| **50** | human | CD183 | CD183 | REAL756 | CD183 | 130-125-  784 | FITC |
| **50** | human | CD38 | CD38 | REAL719 | CD38 | 130-126-  438 | PE |
| **50** | human | CD107a | CD107a | REAL653 | CD107a | 130-126-  203 | APC |
| **50** | human | HLA_DR | HLA-DR | REAL550 | HLA_DR | 130-123-  076 | FITC |

| **50** | human | CD47 | CD47 | REAL250 | CD47 | 130-125-  964 | PE |
| --- | --- | --- | --- | --- | --- | --- | --- |
| **50** | human | MUC1 | MUC1 | C595 | MUC1 | MA1-35942 | FITC |
| **50** | human | FoxP3 | FoxP3 | REA1253 | FoxP3 | 130-127-  808 | PE |
| **50** | human | CD27 | CD27 | REAL173 | CD27 | 130-116-  228 | APC |
| **50** | human | CD206_MRC1 | CD206_MRC1 | REAL518 | CD206_MRC1 | 130-122-  168 | APC |
| **50** | human | CD274_PDL1 | CD274_PDL1 | REA1308 | CD274_PDL1 | 130-127-  907 | PE |
| **50** | human | CD11b | CD11b | REA1321 | CD11b | 130-128-  773 | PE |
| **50** | human | CD11c | CD11c | REA1310 | CD11c | 130-128-  346 | PE |
| **50** | human | CD117 | CD117 | REA787 | CD117 | 130-111-  592 | PE |
| **50** | human | CD4 | CD4 | REA1307 | CD4 | 130-127-  906 | PE |
| **50** | human | CD68 | CD68 | REA130 | CD68 | 130-128-  345 | PE |

**Table S2. List of antibodies used in deep multiplex immune-fluorescence microscopy.**

**
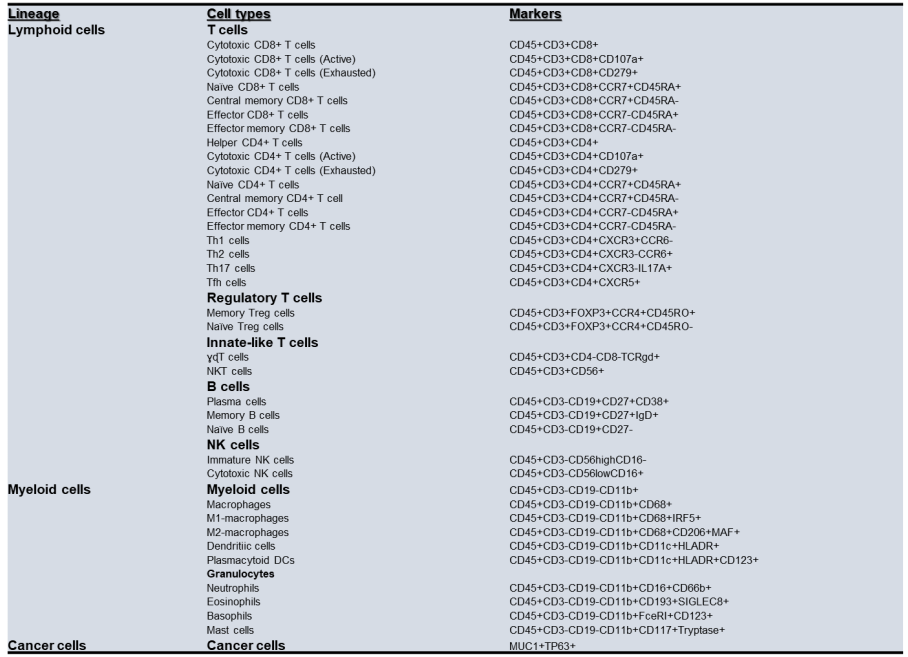
**

**Table S3. Hierarchy of cell phenotyping**
